# Implementing the World Health Organization - Framework Convention on Tobacco Control Article 5.3: A qualitative study in 17 Indian states

**DOI:** 10.1371/journal.pgph.0006522

**Published:** 2026-07-02

**Authors:** Upendra Bhojani, Shivam Kapoor, Amit Yadav, Puneet Chahar, Ashish K. Pandey, Rana J. Singh

**Affiliations:** 1 Centre for Commercial Determinants of Health, Institute of Public Health Bengaluru, Bengaluru, India; 2 Tobacco Control Department, Vital Strategies, New Delhi, India; Nepal Health Research Council, NEPAL

## Abstract

India experiences a high degree of tobacco industry interference. The World Health Organization Framework Convention on Tobacco Control, in its Article 5.3, requires members to protect their policies from tobacco industry influence. Despite the global guidance, the implementation of the Article 5.3 of the convention remains far from desired. We study implementation processes concerning the Article 5.3 policies in 17 of the 19 Indian states that had adopted such policies by 2023. We first conducted an online survey to understand the nature and enforcement of Article 5.3 policies. We then conducted focus group discussions engaging representatives from governments and civil society. The inquiry focused on understanding the prevailing policies, perceived challenges, and good practices in policy implementation as well as the perceived policy impacts. We used reflexive thematic analysis of the qualitative data. There were 17 respondents for the survey, and a total of 258 participants in 17 focus group discussions across study states. Major perceived challenges in the implementation of Article 5.3 policies included limited dissemination/awareness about these policies, suboptimal functioning of the committees overseeing implementation, low priority accorded to these policies, and interference by the tobacco industry. Several government departments were perceived vulnerable to industry interference, with the departments of education, health, and municipal administration perceived as the most vulnerable. A few states adopted practices that facilitated Article 5.3 policy implementation, including display of policy signage/declarations, issue of policy orders/notifications by non-health departments, and setting up a complaints mechanism and norms to avoid sponsorships by private entities. These policies were perceived to have reduced visible interference from the industry and enabled some of the government agencies to refuse funding/partnerships with the tobacco industry. The sub-national Article 5.3 policies in India have a promise to reduce tobacco industry interference. However, implementation of these policies ought to be strengthened.

## Introduction

The tobacco industry uses a range of tactics to influence tobacco-related public policies for commercial gains from the local to the global level. These tactics are manifold, including but not limited to funding of (and undermining) science, funding of political parties, lobbying, public relations, manipulation of media, use of litigation, and intimidation of tobacco control authorities and advocates [1]. In several countries, the high tobacco industry interference has been associated with suboptimal tobacco control measures and high adult smoking rates [2]. The World Health Organization Framework Convention on Tobacco Control (WHO-FCTC) recognizes tobacco industry interference as problematic and, as part of its general obligations (Article 5.3), requires member to protect their public health policies related to tobacco control from commercial and vested interests of the tobacco industry [3]. The WHO-FCTC also developed detailed guidelines about implementing Article 5.3 [4]. By the year 2025, 102 countries reported implementing at least one measure aligned with the WHO-FCTC Article 5.3 implementation guidelines [4,5].

The WHO-FCTC assessment, after a decade of its existence, demonstrated that the Article 5.3 remain one of the least implemented provisions of the treaty [6]. While there is no dearth of global guidance on implementing the WHO-FCTC Article 5.3 [4,7,8], the national and sub-national implementation of this policy remains far from desired. The Global Tobacco Industry Interference Index is an annual global survey wherein civil society actors use publicly available information to capture the nature and extent of tobacco industry interference in participating countries as well as the measures taken by these countries to protect tobacco control policies from the industry influence [9]. The Index assigns a score (0–100) to each country, wherein a bigger score implies a greater degree of tobacco industry interference in that country [9]. In its latest report in the year 2025, this Index revealed that none of the 100 countries surveyed were immune to tobacco industry interference [9]. In fact, among the 90 countries that reported for the year 2023 as well as the year 2025, more than half of these countries (n = 46) showed deterioration in implementation of the WHO-FCTC Article 5.3, while some (n = 34) showed improvement over time [9].

Given this suboptimal and worsening implementation of the WHO-FCTC Article 5.3, we turn the research gaze to the implementation processes for the WHO-FCTC Article 5.3. We focus on India, which we believe is an ideal case for studying implementation. India is the second largest tobacco producer and exporter of unmanufactured tobacco, with a huge domestic tobacco market [10]. It is also home to the second largest number of tobacco consumers in the world after China [10]. Further, reports show that India experiences a high degree of tobacco industry interference. With the Tobacco Industry Interference Index score of 59 out of 100 (the higher the score, the greater the interference), India was ranked at 48^th^ place among the 100 countries assessed in the year 2025 [9]. Given the overall paucity of evidence around implementing the WHO FCTC Article 5.3 and given that several countries, especially in South Asia but also others, share one or more features of the Indian context (federal democracy, heterogenous tobacco industry, diversity of tobacco products beyond cigarettes, large population of Indian states often similar to, or more than individual countries), we believe that lessons from this study will have relevance beyond India.

In the absence of a national policy in line with the WHO-FCTC Article 5.3, India championed a ‘bottom-up’ approach wherein, starting with the Punjab in 2015, several states and at times the districts within states adopted policies to prevent tobacco industry interference [11,12]. By the year 2022, a total of 14 states and union territories (out of 36 such sub-national jurisdictions) in India adopted a policy in line with the WHO-FCTC Article 5.3 (now onward referred to as ‘Article 5.3 policies’ in this paper) [13,14]. Over time, more jurisdictions adopted such a policy, with 23 states by 2025 [14]. Some improvements observed in the implementation of the WHO-FCTC Article 5.3 in India over the recent years (2018–2021) have been in areas of minimizing unnecessary interactions between public officials and the tobacco industry, avoidance of conflicts of interest for tobacco control among public agencies, and adoption of preventive measures. Arguably, at least in part, this improvement is linked to the incremental adoption of Article 5.3 policies at the sub-national level [15].

Despite a decade since the first such sub-national policy was adopted in India, there has been a dearth of research about these Article 5.3 policies, and the available studies have mainly focused on examining policy adoption, contents, and compliance [15–19]. In this paper, we aimed to study the implementation processes/perceptions concerning Article 5.3 policies at the sub-national level in India, challenges faced and good practices adopted by states enhancing the implementation of these policies, as well as the perceived impact of these policies. We believe that learnings from such a study will help enhance the implementation of Article 5.3 policies in these Indian states, safeguarding the prevailing effective tobacco control policy measures and allowing for the adoption and enforcement of stricter tobacco control measures in the future. Such lessons will also encourage and inform other Indian states that are considering adopting Article 5.3 policies, as well as other countries, in conceiving and enforcing sub-national measures to prevent tobacco industry interference.

## Methods

### Study approach

We conducted a qualitative study in 17 Indian states that have adopted Article 5.3 policies in some form, using an initial online self-administered survey followed by focus group discussions (FGD) with relevant stakeholders from governments and civil society. The survey contained questions intended at generating qualitative descriptions of the Article 5.3 policies and their functioning, as well as identifying relevant stakeholders in study states. The FGDs were meant to develop interpretivist accounts by the concerned governments and civil society representatives, and their experiences and perceptions with respect to implementing these policies at the district- and state-level.

### Context

Based on the earlier documentation and our knowledge, we identified 19 states and union territories in India having Article 5.3 policies in some form by the year 2023. The union territories are the administrative units governed directly by the national government. Barring two of the states, where we could not secure administrative approval in time, we conducted the study in 17 states and union territories: (1) Rajasthan, (2) Uttarakhand, (3) Maharashtra, (4) Uttar Pradesh, (5) Bihar, (6) Kerala, (7) Karnataka, (8) Chhattisgarh, (9) Jammu & Kashmir, (10) Assam, (11) Punjab, (12) Nagaland, (13) Puducherry, (14) Jharkhand, (15) Meghalaya, (16) Tamil Nadu, (17) Madhya Pradesh. For this paper, we will use ‘state’ as a generic term to refer to each of these sub-national units, even if a few of these (Puducherry, Jammu & Kashmir) were union territories at the time of the study. Taken together, the 17 states included in the study account for about 72.5% (about 102.4 million in the year 2025) of India’s population.

### Sampling, data collection, and analysis

While we describe each method separately, the tools (questionnaire, and the FGD guide) used for both were refined following (1) a validation exercise [20,21], wherein a panel of experts were asked to score/comment on face validity (importance of the questions) and content validity (relevance, clarity, and completeness of the questions); and (2) field pilots in two of the non-study states.

#### Survey.

We sent out an online self-administered questionnaire as a Google Form. We purposefully selected one adult respondent from each study state, who was actively working in tobacco control and had a close working relationship with the tobacco control cell (department of health) of the respective state governments. Respondents included a representative of the partner organizations of the Tobacco Control Department of Vital Strategies from 12 states, one of the project staff working locally with state tobacco control cells in four of the states (i.e., Karnataka, Chhattisgarh, Jammu & Kashmir, Assam), and an official from the state tobacco control cell of Nagaland. All 17 respondents consented and returned the completed questionnaires.

The questionnaire had two parts: one enquiring about the Article 5.3 policy (issuing authority, scope, formation and functioning of overseeing/implementing body, and reporting of violations) and the other about the complaints received and action taken (if any). Respondents filled in the questionnaire in close consultation with the officials from the respective state tobacco control cell with whom they had a close working relationship. This process was completed between May 23 (2023) and November 7 (2023). Data generated through questionnaire forms were downloaded in MS Excel format. We analyzed the data (with mostly yes/no or short descriptive responses), generating brief Article 5.3 policy descriptive profiles of each study state. These findings helped us understand the key features of Article 5.3 policies in states and identify important stakeholders for FGDs.

#### FGDs.

We conducted a total of 17 FGDs, one in each study state. The principal investigator (SK) and a few of the co-investigators (PC, GB, AY), along with the facilitators (representatives of the partner organizations of the Tobacco Control Department of Vital Strategies) in each state, conducted the FGDs. These facilitators, employees of the partner organizations, helped in recruiting potential participants. Facilitators were trained through an online workshop about the purpose of conducting FGDs, the FGD guide, and the process. Participants were purposefully chosen based on findings from the survey and ensuring representation from the state tobacco control cells of health departments, other health department divisions (e.g., food safety, mental health, non-communicable diseases, oral health) as well as representatives from the non-health departments of state governments (e.g., education, municipal corporations, tourism etc.) and select civil society entities including non-government organizations (NGOs) and academic institutions that were active in tobacco control in the state. The potential participants were invited to an in-person FGD.

Participants were briefed about the tobacco industry interference, WHO-FCTC Article 5.3, and summary of the survey findings (about Article 5.3 policy) for that state, inviting feedback on any updates/corrections. The investigators and facilitators then initiated and moderated the discussion by bringing in lead questions from the FGD guide that were related to understanding awareness among stakeholders about the policy, challenges faced in implementing the policy, implementation mechanisms, perceived compliance with the policy and its impact, and participants’ suggestions for enhancing the implementation of these policies.

The FGDs lasted for an average of 90 minutes (40–130 minutes), and 10 of the 17 were audio recorded. For the rest, we took detailed notes as we either did not get the consent for audio-recording (n = 2) or the venue was too large, rendering the recording ineffective (n = 5). These hand-written notes were made by assistants to the facilitators and were later reviewed/edited by the facilitator for any missing/misinterpreted inputs. The audio recordings and handwritten notes were transcribed and translated into English (where needed). These FGDs were completed between June 12 (2024) and October 13 (2024).

For FGDs, MS Excel was used to organize the transcripts. The data was coded based on pre-determined inquiry probes (i.e., awareness, challenges in implementation, implementation mechanisms, and suggestions for improvements) by UB, who has training in qualitative data analysis and has published qualitative studies. We could not use independent coding by more than one researcher, mainly due to resource constraints. We used reflexive thematic analysis following the six-step process described by Braun and Clarke [22]: familiarization with data, generating initial codes, searching for themes, reviewing themes, defining and naming themes, and writing the report. Group dynamics and corroborated data were considered, where possible, from responses of participants across sectors (governments, civil society) and across positions within and across the government departments.

### Ethics

The study was formally reviewed and approved by the Ethics Advisory Group at The Union (Ref: 07/2022) and the Institutional Ethics Committee at the Institute of Public Health Bengaluru (Ref: IPH/23-24/E/2). For both the survey and the FGDs, written informed consents were sought from participants after briefing them about the study and the rights of the participants.

## Results

### Characteristics of the survey respondents

17 individuals (12 men, 5 women), one from each study state, filled the survey. They included 12 civil society representatives working closely with state governments and five respondents working with state tobacco control cells within health departments.

### Characteristics of the FGD participants

**Fig 1:**
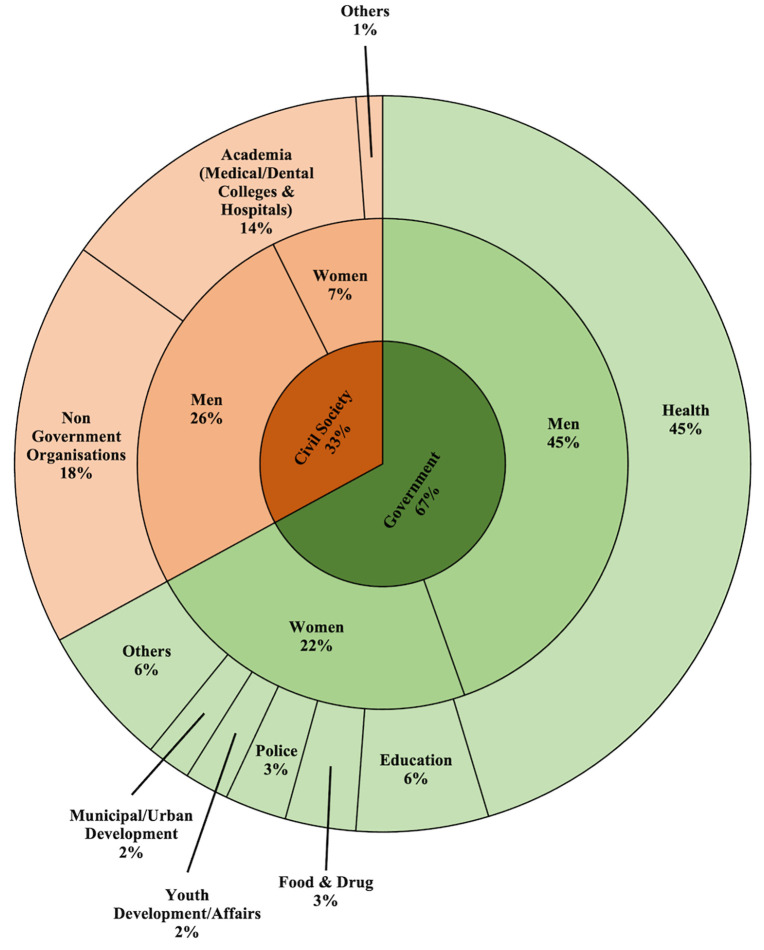
Characteristics of FGD participants. Fig 1 provides the distribution of FGD participants as per their gender and the sector (government, civil society) they worked in.

A total of 258 participants (181 men, 77 women) participated in 17 FGDs. On average, there were 15 participants per FGD, ranging from a minimum of 12 and a maximum of 20 participants. Government representatives accounted for 67% of participants and came from 16 departments with a majority from health department. Civil society representatives accounted for 33% of participants. See Fig 1 for sector- and gender-based distribution of FGD participants.

We have anonymized the states replacing their names with unique numbers while providing FGD excerpts. This is to prevent the identification of participants because each state had a very few staff members manning the state tobacco control cell/committee with a single nodal officer for tobacco control.

### About article 5.3 Policies in Indian states

According to survey participants, Punjab was the first state to adopt an Article 5.3 policy in July 2015, and Nagaland was the latest within the study period, notifying the Article 5.3 policy in June 2023. In all the states, the policy was issued by a senior officer from the health department, except in Chhattisgarh, where it was done by the state nodal officer for tobacco control. See Table 1 for the details on attributes of the Article 5.3 policies issued by the states as gathered from the survey.

**Table 1:** Features of the Article 5.3 policies issued by the states in India.

States	Type of the (Article 5.3 policy) document	Policy issue date (DD/MM/YYYY)	Policy issuing office	Is the policy applicable to non-health departments?	Is there a provision in the policy for constituting a body to oversee implementation?	Was such a body overseeing the policy implementation constituted?	Was such a body overseeing the policy implementation active or operational?
Punjab	Notification	13/07/2015	Principal Secretary (Health Department)	Yes	Yes	Yes	Yes
Bihar	Notification	23/06/2017	Special Secretary (Health Department)	Yes	Yes	Yes	No
Jammu & Kashmir	Circular	27/07/2017	Additional Secretary (Health and Medical Education Department)	Yes	Yes	No response	No response
Tamil Nadu	Order	10/11/2017	Principal Secretary (Health Department)	Yes	Yes	Yes	No
Jharkhand	Notification	01/10/2018	Principal Secretary (Health, Medical Education & Family Welfare Department)	Yes	Yes	Yes	Yes
Karnataka	Notification	25/01/2019	Under Secretary (Health & Family Welfare Department)	Yes	Yes	Yes	Yes
Kerala	Order	30/04/2019	Additional Chief Secretary (Health & Family Welfare Department)	Yes	Yes	Yes	Yes
Meghalaya	Order	26/06/2019	Secretary (Health & Family Welfare Department)	Yes	Yes	Yes	Yes
Uttar Pradesh	Notification	16/09/2019	Secretary (Health & Family Welfare Department)	Yes	Yes	Yes	Yes
Chhattisgarh	Letter	02/02/2021	State Nodal Officer (National Tobacco Control Program)	No	No	Not Applicable	Not Applicable
Rajasthan	Circular	28/10/2021	Secretary (Medical Health & Family Welfare Department)	Yes	No	Not Applicable	Not Applicable
Puducherry	Order	27/12/2021	Chief Secretariat (Health Department)	Yes	Yes	Yes	No
Madhya Pradesh	Order	13/01/2022	Director (National Health Mission)	No	No	Not Applicable	Not Applicable
Uttarakhand	Order	05/05/2022	Secretary (Health & Medical Education Department)	Yes	Yes	Yes	No
Maharashtra	Notification	18/07/2022	Additional Secretary (Public Health Department)	Yes	No	Not Applicable	Not Applicable
Assam	Order	09/03/2023	Principal Secretary (Health & Family Welfare Department)	Yes	Yes	Yes	No
Nagaland	Notification	16/06/2023	Commissioner & Secretary (Health and Family Welfare Department)	Yes	Yes	Yes	Yes

Some design features within the Article 5.3 policies limited their implementation. Six of the 17 states had no provision for creating a body that oversees policy implementation (Table 1). While 10 out of the remaining 11 states had constituted an overseeing body (routinely called an ‘empowered committee’), only six such bodies had convened at least one meeting, deliberated about Article 5.3 policy issues, and/or taken actions. Furthermore, in two of the 17 states studied (Chhattisgarh, Madhya Pradesh), the Article 5.3 policies issued by health departments were applicable within health departments excluding non-health departments in their scope.

Some FGD participants argued that ideally, a policy at the state level ought to be issued through a cabinet approval and published in the state gazette. Such policy will be seen with a greater legitimacy compared to an order issued by an executive.

*“It (Article 5.3 policy) isn’t a policy document… It is not issued through cabinet approval…it is an executive order at the state level.”* (FGD-13)

In absence of a cabinet approved gazette policy, an order issued by a senior bureaucrat within the health department (typically a secretary, or a commissioner) evokes better legitimacy compared to the one issued by lower-level officer.

*“We faced challenges in convincing superiors of the need to issue a policy…So, we issued it, but we realized that at the district level, district collectors are not giving any importance to the policy as it is not issued by the department head”* (FGD-10)

### Challenges in policy implementation

We describe the major themes defining challenges as reported by the FGD participants in implementing Article 5.3 policies in states.

#### Low awareness regarding the Article 5.3 policy.

Participants from government agencies had limited awareness about Article 5.3 policies. In nine of 17 states, the awareness about the presence of Article 5.3 policies was mainly limited to health department officials, and at times, including officers from education and food safety (and drug control) departments. In one of the states, a few health officers also did not know about the policy in their state.

In the other six states, nearly all the participants representing various government departments were aware of the Article 5.3 policy in their state. However, even in these states, the detailed knowledge about various provisions in these policies was reported mainly by participants from health departments.

Limited dissemination of Article 5.3 policies was the main reason for low awareness about these policies among stakeholders. We understood from participants that, typically, Article 5.3 policies issued at the state level were to be sent to the office of district administration (referred variably as district magistrate, commissioner, or collector) with an expectation that the district administration would further disseminate the policy to various department heads in respective districts. However, this was not always the case and at times, the district-level dissemination was delayed or yet to happen.

*“For now, the (Article 5.3 policy) copy has been issued to all EC (Empowered Committee) members. After the first EC meeting, NTCP (national tobacco control program) will write letters to districts (regarding Article 5.3 policy).”* (FGD-17)

Even when Article 5.3 policies were sent to district administration, they did not necessarily reach relevant officers at sub-district level.

*“The order (Article 5.3 policy) was sent to all the district magistrates for further action. But only five district magistrates sent it to other departments in their respective districts. This gap needs to be solved by sending the order again to all district magistrates and directing them to send it further to various departments in their districts.”* (FGD-7)

Representatives of the NGOs that participated were aware of the Article 5.3 policy. However, many of the representatives from academia (mainly medical/dental colleges) were unaware of the Article 5.3 policy adoption by their respective states, even though some of them were aware of Article 5.3 in the global treaty (i.e., the WHO FCTC).

*“It (Article 5.3 policy) is not sent to us.” “I have no idea of such a policy (Article 5.3 policy) in my state. I have not received a copy of such policy…It (Article 5.3 policy) should be circulated to all the organizations, all the academic institutions.”* (FGD-14)

Participants reported that in four of the 17 states, the state-level coordination committee on tobacco control (a body under the national tobacco control program) had discussed Article 5.3 policy in its agenda for one or more times, focusing on its adoption, dissemination, and/or enforcement. In one of the states, the high-powered committee on tobacco control (chaired by the chief secretary) had discussed Article 5.3 policy. Based on participants’ inputs, none of the states studied had issued a public notice or undertaken measures (e.g., mass media use) to raise public awareness about the Article 5.3 policy.

#### Sub-optimal functioning of empowered committees.

In 12 of the 17 states, Article 5.3 policies prescribed the constitution of an empowered committee to oversee the implementation. Participants from some study states highlighted that these committees, while constituted in these states, have not met since their constitution. Participants suspected that such was likely a case for many more states.

*“The empowered committee under the policy has not met even once since its formation.”* (FGD-3)

Some participants believed that the lack of an active and functioning committee hindered state tobacco control cells from taking further steps needed for implementing Article 5.3 policies.

*“No specific meeting has happened of the WHO FCTC Article 5.3 state-level empowered committee. If such review meetings happen regularly, senior officials will get sensitized about this policy.”* (FGD-6)

#### Lack of clear guidance for implementation.

Participants from many states highlighted that Article 5.3 policies in their present shape lacked clear guidance for implementation: role of empowered committee, defining tobacco industry, complaint mechanism and penal actions.

*“There is a lack of clarity…among the government officials. There are no clear guidelines on what must be done. Also, we don’t know what constitutes the tobacco industry, whether the manufacturers, retailers, or wholesalers.”* (FGD-8)

#### Low priority accorded to the Article 5.3 policy implementation.

Participants highlighted that non-health departments often accorded low priority to implementing Article 5.3 policies. The turnover of staff within other departments was also perceived as a barrier, especially as there was no system for periodic sensitization/training about the Article 5.3 policy.

*“…they (officers from non-health departments) give low priority to tobacco control as they all have their own priorities…There has been much movement of staff in the police department, so those trained in tobacco control often moved to other departments.”* (FGD-12)

At times, the Article 5.3 policy implementation agenda got delayed in the face of the changes in high-level administrative and political leadership, and other pressing priorities for health departments.

*“The first EC (Empowered Committee) meeting was to be held right after the (issue of the) notification (Article 5.3 policy). It got delayed due to a change in the leadership at the Secretariat level.”* (FGD-17)

*“COVID-19 pandemic that started after adopting the policy seems to have sidelined the policy and its implementation agenda.”* (FGD-7)

#### Tobacco industry interference within the Article 5.3 policy implementation.

Participants from two states reported that while the adoption of the Article 5.3 policy in the state reduced and/or stopped the tobacco industry’s overt outreach to senior health officials, the industry was now engaging directly with lower-level officials, at times in the disguise of non-government organizations or the industry front groups. Front group is generally understood as an entity or organization that “purports to represent one agenda while in reality it serves another party or interest whose sponsorship is hidden or rarely mentioned.” [23]

*“…for example, the industry representative approached the municipal corporation officer who had recently taken charge (newly joined the position) and requested a meeting with enforcement officers to discuss the point-of-sale advertisements.”* (FGD-5)

Participants from one of the states flagged that tobacco industry representatives often approached political leaders to bypass senior health department officials.

*“Tobacco Industry representatives are often approaching elected political leaders directly (bypassing the senior bureaucrats) about tobacco regulations.”* (FGD-6)

**Fig 2:**
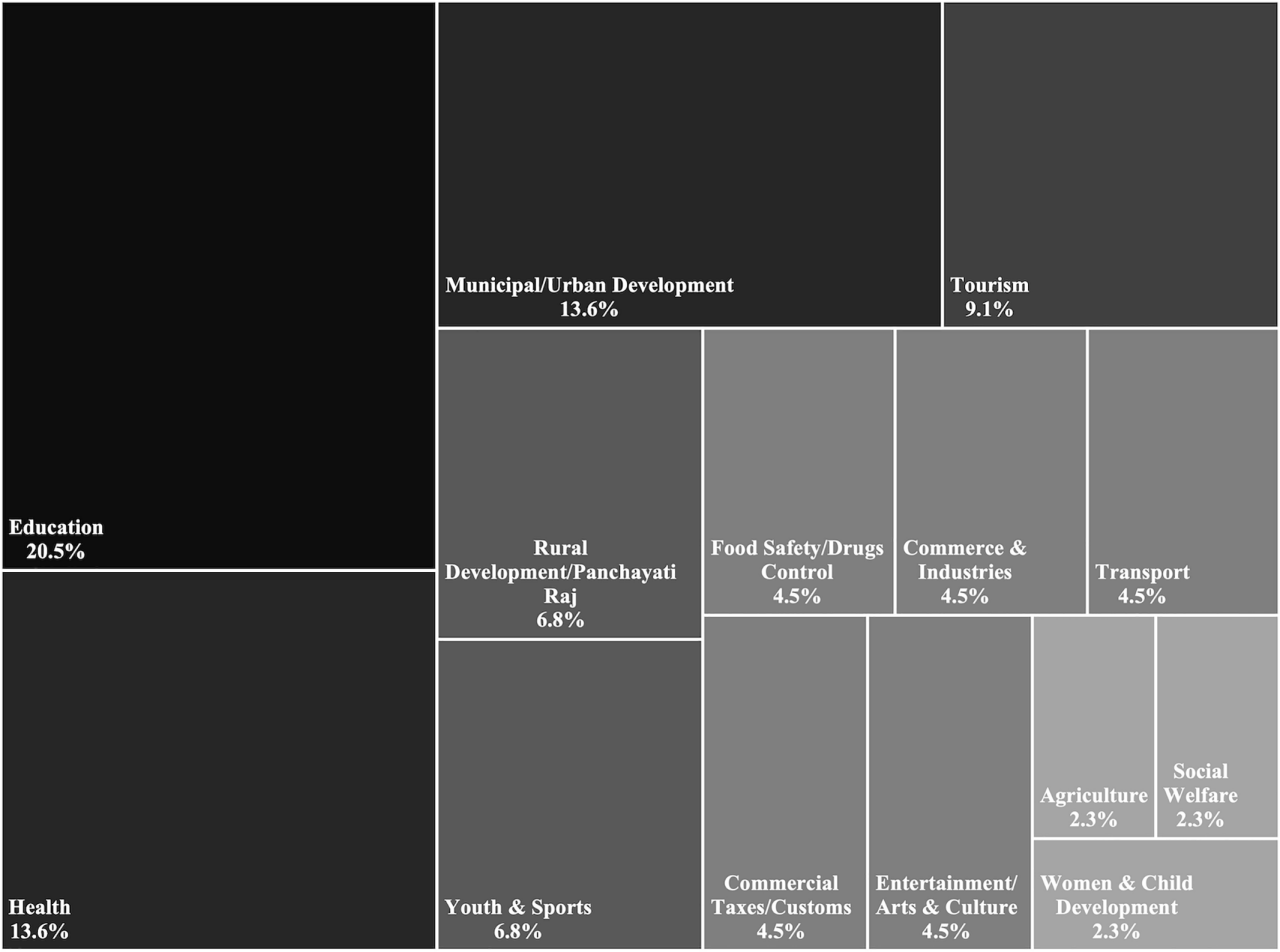
Perceived vulnerability of government agencies for tobacco industry interference. Percentages in Fig 2 refer to the frequency of times the given department was mentioned as being vulnerable to tobacco industry interference by FGD participants.

Participants from 13 out of the 17 states studied mentioned specific government departments that they felt were particularly vulnerable to industry interference. Such risk perception was based on the incidence of the industry interference in the past, as well as the potential that certain departments offered to the industry to reach children/youth as potential consumers through its so-called corporate social responsibility (CSR) activities/partnerships. The education department was perceived to be the most vulnerable, followed by the health and the municipal administration departments. Fig 2 depicts the perceived risk of the industry interference for the various government departments.

Participants from many states mentioned how the lack of real-time monitoring of tobacco industry interference in states makes it difficult to implement and evaluate Article 5.3 policies. Many within state tobacco control cells and civil society sense the tobacco industry interference, but the lack of systematic monitoring and documentation hinders corrective actions.

### Good practices in the implementation of the Article 5.3 policy

We highlight practices within Indian states that facilitated Article 5.3 policy implementation despite challenges described in the earlier section.

#### Display of Article 5.3 policy and related signage.

Participants from a few states reported that Article 5.3 policies in their states had the provision of displaying signage (information boards) at government offices.

*“The policy is disseminated through the advisory issued to all the district heads (of departments) for prominently displaying the FCTC (Article 5.3 policy) signages in all government offices…the display boards are prominently put up at government offices and institutions.”* (FGD-5)

*“Signage boards highlighting the (Article 5.3) policy are present in government offices, which are visible to all.”* (FGD-9)

This was perceived to be a useful practice in raising awareness about Article 5.3 policies within government departments and those visiting these offices. Participants from three of the 17 states (Kerala, Jharkhand, Uttar Pradesh) mentioned about display of such signage at government offices mentioning the need for the tobacco industry to seek prior permission or follow a specific procedure if it intended to meet with government officials.

Though not mandated by Article 5.3 policies, some of the government and civil society representatives from two of the states (Assam, Maharashtra) reported having displayed Article 5.3 policy and/or a declaration about them not partnering with or taking funds from the tobacco industry on their organizational websites.

*“To uphold transparency, some of the organizations in the state have displayed a conflict of interest declaration on their websites. This visual representation underlines the commitment to transparency… setting clear expectations regarding the processes required before any interaction between the tobacco industry and government agencies.”* (FGD-16)

*“No signage is being shared by any office, but it (Article 5.3 policy) is displayed on the government department website.” “It (Article 5.3 policy) is displayed on XXX (civil society organization) official website.”* (FGD-15)

#### Issue of Article 5.3 policy orders by non-health departments.

In a few states, the issue of Article 5.3 policies by the health department followed the issue of similar orders/notifications by select non-health departments, enhancing awareness about and compliance with these policies within these departments. Typically, departments in the education sector issued such orders/notifications as mentioned by participants from two of the states (Jharkhand, Madhya Pradesh).

*“A comprehensive order (about Article 5.3 policy) has been issued by the Department of School Education and Literacy Development as well as the Department of Higher and Technical Education, to check tobacco industry interference in educational institutions in any form.”* (FGD-5)

*“So, at the state-level, there have been two orders (about Article 5.3 policies). The Director NHM (National Health Mission) released an order… Secondly, XXX, who was an additional commissioner in higher education, released an order about the Article 5.3 policy.”* (FGD-13)

#### Attempts at monitoring the tobacco industry interference.

While there seems to be a lack of systemic monitoring of tobacco industry interference by governments, participants from three of the states reported some progress in this direction. One of the states (Maharashtra) designated state and district-level tobacco control committees to also serve as monitoring committees for the implementation of Article 5.3 policies. Participants from two of the states (Uttar Pradesh, Tamil Nadu) reported the use of WhatsApp groups among health department officials and a phone-based health helpline as sources of receiving complaints and/or intelligence about tobacco industry interference in these states.

*“Monitoring information (about tobacco industry interference) often comes from district-level officials and WhatsApp groups.”* (FGD-9)

*“Complaints have been received under Article 5.3 (policy) through the medical helpline number (104), and actions are being taken.”* (FGD-4)

#### Normative measures in line with the WHO FCTC Article 5.3.

The participants highlighted certain normative measures – beyond mandated provisions of Article 5.3 policies – taken by health and non-health departments in a few states that helped enhance compliance with Article 5.3 policy. In one of the states, health department officials set a norm that tobacco industry representatives wanting to engage with the health department need to first engage with the high-level offices (typically, the health secretary and/or the director of the national health mission). This arrangement was in anticipation of the focused and better implementation of Article 5.3 policies.

*“The PS (Principal Secretary) and MD (Managing Director) of NHM (National Health Mission) are the designated points of communication for the tobacco industry. The representatives of the tobacco industry cannot meet with just anyone and must adhere to these specific channels.”* (FGD-9)

In another state (Karnataka), in lieu of a long-pending meeting of the state-level coordination committee, the agenda related to the Article 5.3 policy was put to the high-powered committee on tobacco control, chaired by the chief secretary established in the state. This helped overcome the status quo on Article 5.3 policy implementation. The police department in one of the states seems to have adopted rules preventing sponsorships from any private organizations (and hence, also from the tobacco industry).

*“We (police department) do not have the Article 5.3 policy as such…However, a general awareness has been created among department officials not to use tobacco, tobacco smoking, or spitting. In the department, we have rules about not taking any sponsorship from private organizations (this response was in relation to the discussion about sponsorship to public agencies by the tobacco industry).”* (FGD-10)

### Perceived impact of Article 5.3 policies

Eight of the 19 states reported having received complaints about or taken Suo motu (on its own accord) cognizance of the violation of the Article 5.3 policy. Policy violations typically included tobacco industry entities partnering with, or providing funds to, government agencies, often as part of their so-called CSR activities. Typically, the response from the state governments took the form of the state-level coordination committee for tobacco control issuing a letter to the concerned government agency requesting it to dissociate itself from the tobacco industry. Such actions typically resulted in termination of, or at least no further expansion of, tobacco industry partnership/funding. Table 2 provides a brief qualitative account of the nature of the violations under the Article 5.3 policy and resolutions for each of these eight states.

**Table 2:** A brief account of Article 5.3 policy violations and their resolutions in select Indian states.

States	Policy violation and/or action
Jammu & Kashmir	In a few of the districts, district administration and police received sanitizers and protective gears from the tobacco industry during the COVID-19 pandemic. This was publicized in print media. The state tobacco control cell wrote a letter to the Director (health department), who in turn wrote to all the districts (administration) advising against any such future funding/partnerships.
Maharashtra	The state received complaints about a medical college, a general hospital, and some schools receiving funding through the so-called CSR activities by the tobacco industry. The state issued letters to respective authorities, including the medical education department and concerned district nodal officers. At times, the nodal officers and/or district committee member/s made visits to the government agency in question. This resulted in the termination of such funding/partnerships.
Assam	There was probably no formal complaint, but the state tobacco control cell took notice of the support being received by the social welfare department from the tobacco industry for a nutrition-related program. Subsequently, a letter was sent from the health department to the social welfare department highlighting the Article 5.3 policy. It was unclear what the impact of such a letter would be on this specific funding/partnership.
Karnataka	There were several complaint-based and/or Suo-motu actions about government agencies receiving funds/partnerships from the tobacco industry (e.g., tobacco industry partnering with municipal agencies for solid waste management; schools receiving funds from the tobacco industry for amenities; tobacco industry partnering with watershed development department). Actions – mainly in the form of letters/notices from health and/or education departments – resulted in the termination of funding/partnerships in most, but not all, such instances. The state tobacco control cell alerted higher officials in the health department that the tobacco industry was approaching with a proposal to build a hospital in a public-private-partnership mode. Subsequently, such a proposal did not materialize.
Tamil Nadu	There were complaints received through the telephone-based medical helpline, and the state reported having taken action. A specific example was of the tobacco industry supporting a ‘spell-bee competition’ among government schools. A letter to schools (by the education department) stopped such partnerships.
Meghalaya	There was probably not a formal complaint, but the state tobacco control cell came to know that the tobacco industry was approaching a university to provide research support/funding. The state tobacco control cell reached out to that university and was able to prevent the funding/partnership.
Uttarakhand	The state received a complaint about the tobacco industry supporting an educational institution in the state. The state tobacco control cell intervened and stopped the funding/partnership.
Uttar Pradesh	The state received eight to ten complaints, all of which were related to government agencies receiving funds/partnerships/benefits/unnecessary interactions under the so-called CSR initiatives of the tobacco industry. Except for two of these complaints, the rest were resolved. The actions were mainly in the form of letters/notices from the administration to the government agency in question and resulted in the ending/dilution (removal of industry logos) of funding/partnerships in most, but not all. The unresolved complaints included tobacco advertisements on public transport buses, as the prevailing regulations did not provide clarity on the removal of such advertisements.

Apart from complaint-based actions, participants articulated other forms of perceived impact of this policy. In one of the states, the civil society representative felt the major tobacco companies stopped publicizing their so-called CSR activities once the state adopted the Article 5.3 policy. However, this may not necessarily mean that they stopped doing these activities.

*“After the (Article 5.3) policy, major cigarette companies in the state (names a couple of them) stopped publicizing their CSR activities.”* (FGD-11)

In another state, a civil society representative mentioned how some of the high-level government officials across departments (especially, health and police) had refused an offer of partnership by the tobacco industry (as part of its so-called CSR initiative), indicating a positive impact of the Article 5.3 policy.

*“While it is difficult to say about the impact of the Article 5.3 orders in concrete terms but there have been instances where higher officials have refused CSR from tobacco companies, which is a positive indicator of the impact of the Article 5.3 orders.”* (FGD-13)

In yet another state, a civil society representative working closely with the state tobacco control cell indicated that a visible outreach by the tobacco industry to health officials in the state, which was a commonplace, stopped after the Article 5.3 policy adoption by the state. Importantly, many participants across states mentioned how difficult it is to comment on the impact of the policy, as there was no real-time monitoring of tobacco industry interference in states.

*“As there is no active monitoring and awareness (about tobacco industry interference), no complaints are registered/received under this (Article 5.3) policy. Other (non-health) departments are not implementing it, not prioritizing it.”* (FGD-12)

## Discussion

We studied the implementation of Article 5.3 policies in 17 Indian states through a self-administered survey and FGDs involving concerned government and civil society representatives. We identified several barriers impeding effective implementation of these policies, including limited dissemination and awareness about these policies (especially at the district-level and among non-health department officials), suboptimal functioning of the empowered committees constituted to oversee policy implementation, relatively low priority accorded to these policies (especially by non-health departments), and interference by the tobacco industry. The participants identified several government departments that they perceived as vulnerable to tobacco industry interference. We also identified practices by a few states that are facilitating Article 5.3 policy implementation including public display of physical signages and web-based declarations related to Article 5.3 policy, issuing of Article 5.3 policy orders by non-health departments, instituting channels (WhatsApp groups, telephone-based health helpline) to receive complaints about the Article 5.3 policy violations, gatekeeping of industry outreach to government agencies by senior officials, and adopting a norm to not take sponsorships by private entities in government departments.

### The promise of bottom-up regulatory initiatives

Overall, our findings imply that despite some years (2–10 years) of having Article 5.3 policies in place, there remain several challenges in their optimal implementation. However, despite these persistent challenges, the perceived positive impact of these policies in the form of a visible reduction in industry-government interaction and some instances of refusals by government agencies to CSR-based partnership offers of the tobacco industry indicate the importance of these policies. In fact, these bottom-up district- and state-level regulations, in the absence of a national (all-of-the-government) level Article 5.3 policy, are not just important but also promising in terms of the incremental increase in their coverage over time. Bassi et al. [18] had identified and analyzed 13 states that had adopted Article 5.3 policies by 2019. Our study could identify 19 states with state-level Article 5.3 policy in some form by 2023. More recently, this number has gone up to 23 by the year 2025 [24].

This phenomenon is not isolated in Indian context as many of the tobacco control regulations in India were initiated at state level (a few even at district level) before they were enacted at national level (e.g., prohibition of electronic nicotine delivery systems [25]) or adopted by most or all of the sub-national jurisdictions across India (e.g., prohibition on certain forms of smokeless tobacco [26]; prohibition on sale of single/loose cigarettes [27]). The Indian constitution divides legislative power across national and state governments.[28] While tobacco industry (especially the levy of excise) comes under the legislative power of national government, health (especially, “Public health and sanitation; hospitals and dispensaries”) comes under the legislative power of state governments [28]. It is this mandate of protecting and promoting health that has prompted state governments to champion tobacco control policies in India.

There are a very few other examples of sub-national level policies in line with the Article 5.3. For example, while the United States of America has not signed the WHO-FCTC, the California Tobacco Control Program endorses guiding principles of the WHO-FCTC Article 5.3 [29]. It prohibits the grantee to have any conflicts of interests with commercial tobacco industry. In a global tobacco control context, where the adoption of policies in line with the WHO FCTC Article 5.3 remains limited, the Indian experience indicates the promise of bottom-up regulations in federal democracies. We now discuss our findings in relation to a few relevant studies done in the past and highlight the major implications for enhancing the implementation of these policies in Indian states.

### Plugging design gaps hindering implementation

The limited number of studies that have analyzed Article 5.3 policies in Indian states have mainly focused on their contents, highlighting several gaps in these policies vis-à-vis the provisions in the implementation guidelines for Article 5.3 of the WHO FCTC [18,24]. While these gaps represent weaknesses in the design of these policies, our study highlights how some of these gaps – not issuing the policy through a higher office/state gazette, not clearly defining the tobacco industry, not defining the role of the empowered committee, not defining the process of how complaints/violations can be reported and resolved – hinder the implementation of the prevailing Article 5.3 policies. Some of these design gaps, such as the lack of specific role of empowered committees and the lack of penal provisions, are likely to result in reduced priority and accountability towards these policies among officials as well as reduced fear in minds of those who violate the policy provisions. These later mechanisms (i.e., feeling accountable, fear of penalty) are some of the important mechanisms that explained implementation outcomes of tobacco control policies in Indian states in a realist evaluation of tobacco control policies by Hebbar et al. [30] Hence, either through amendments to the existing policies or through the notification of rules under the existing policies, there is an imminent need to address these gaps and make these policies enforceable.

### Raising awareness about Article 5.3 policies

When it comes to implementing the existing Article 5.3 policies, the persistent challenge has been the very limited awareness of these policies among government officials and stakeholders, especially so among officials in non-health departments and at district/sub-district levels. Bassi et al. [18], in their qualitative study exploring Article 5.3 policy dynamics in 13 Indian states (2015–2019), noted very limited engagement from non-health department stakeholders on Article 5.3 policy. Kumar et al. [31], in their study exploring implementation challenges related to Article 5.3 policies in the four districts of the southern Indian state of Karnataka, revealed that awareness and understanding about tobacco industry interference and the Article 5.3 policy were low even among stakeholders directly engaged in tobacco control as members of district-level (tobacco control) committees.

The challenge of poor awareness about the industry interference and the Article 5.3 policy is not limited to India. Barry et al. [32] recognize these issues in their study across India, Bangladesh, Ethiopia, and Uganda. A study in Thailand revealed that those tobacco control stakeholders who were more aware of tobacco industry interference tactics also had more positive/favorable attitudes towards the Article 5.3 policy [33].

In our study context, the limited awareness was primarily the result of poor dissemination of policy from the state to district levels and to various heads of non-health departments through established administrative channels. There is a need to periodically issue circulars about Article 5.3 policies from the state health department to district offices, ideally complemented by the issue of such circulars by each non-health department, ensuring vertical dissemination within departments. What will especially help this endeavor is if such circulars require the display of the signage about Article 5.3 policies at conspicuous places within government offices, the display of Article 5.3 policy on departmental websites, and periodic sensitization events to raise awareness about these policies within government offices. Additional efforts at raising awareness among civil society organizations and the public at large are also crucial to build demand for the enforcement of these policies. This implies the need for public-facing awareness campaigns using mass media (including public notices in prominent news dailies) and sensitizing concerned civil society actors (especially academia, health-related NGOs, and journalists).

### Advocating for implementation processes

Our study points to the need to turn the gaze on implementation processes for Article 5.3 policies in Indian states. There is a need to push for clarity about the roles and functioning of the empowered committees, as in most states, these committees have not met once since their constitution. This finding corroborates the earlier observation by Bassi et al. [18] of poor regulatory capacity of such committees in Indian states. Barry et al. [32] also noted limited capacity for intersectoral convening mechanisms at state and national levels in countries in Asia and Africa (India, Bangladesh, Ethiopia, Uganda). In this context, some of the practices adopted by Indian states in implementing Article 5.3 policies indicate the importance of tailoring to and institutionalizing Article 5.3 policy implementation within prevailing norms/practices of specific government departments rather than one-size-fits all approach, e.g., issuing of notification/circulars by non-health departments about Article 5.3 policy; utilizing WhatsApp group of (tobacco control) program-specific staff to share intelligence about tobacco industry interference; or utilizing existing telephone-based health helpline or existing tobacco monitor apps on phones for receiving complaints regarding violations of Article 5.3 policies.

Furthermore, having a national policy in line with the WHO FCTC Article 5.3 and integrating it into processes of the National Tobacco Control Program is likely to enhance implementation and monitoring/review of Article 5.3 policy implementation. In fact, the Ministry of Health and Family Welfare (Government of India) adopted a code of conduct to prevent tobacco industry interference in the year 2021, but its scope has been limited to the health institutions that are directly under the control of the ministry [34]. A nation-wide opinion poll of tobacco control stakeholders conducted in December 2021 revealed an overwhelming support (65% “strongly agreed”, 22.6% “agreed”) for a nation-wide Article 5.3 policy in India [35]. There have been repeated appeals for adopting such a policy in India [36]. Hence, the Ministry of Health and Family Welfare (Government of India) shall catalyze the development of a nationwide Article 5.3 policy for India as well as encourage states to adopt/implement Article 5.3 policies.

### Monitoring tobacco industry interference

Many participants in our study pointed to the lack of any functional institutional system for real-time and/or periodic monitoring of tobacco industry interference. Civil society organizations in India have produced the tobacco industry interference index at the national level on an annual basis since 2018. While this exercise itself remains voluntary and ad hoc (the last index we could locate was for 2023), it indicates a high level of tobacco industry interference in tobacco control. In 2025, India was ranked at 40^th^ place in a survey of 90 countries in terms of implementation of the WHO FCTC Article 5.3, with an index score of 58 out of 100 (the higher the score, the greater the industry influence) [34]. Such exercise is rare at the state (sub-national) level. A few such attempts at the sub-national level indicate that it is feasible and important to monitor tobacco industry interference at the state level, as such influence remains high and variable across states [19,37]. More importantly, such exercise ought to be institutionalized to ensure its sustenance and linkage with policy/practice response. As attempted by one of the states under this study, such monitoring could be done through existing committees or their sub-groups (such as state- or district-level coordination committees for tobacco control constituted under the National Tobacco Control Program) and ideally include experts from academia and civil society. Such a forum shall bring out periodic reports and provide intelligence to the empowered committee under the Article 5.3 policy for action.

### Limitation

While the embeddedness of this study – wherein participants included government officers across departments with leadership and/or professionally mandated roles for tobacco control – is the core strength of this study, it affected the rigor of data collection process. It was difficult to get adequate participation from non-health departments, given their hectic work schedules and competing priorities. The hierarchical pattern of reporting and relationships within and across government departments impacted the free and candid expression by participants, especially when senior bureaucrats (secretaries, commissioners, directors) participated. This would have potentially created some degree of a social desirability bias [38]. The same could have been the case for a few of the civil society representatives, whose work demanded close working with and/or funding from state governments. Hence, there was a blurring of lines between a typical FGD generating in-depth engagement of a small closed group and a workshop engaging stakeholders without a pretense of non-hierarchical interaction within the group [39]. However, we believe that a relatively large participation across 17 FGDs in diverse state contexts would have ensured that we did not miss any major perceived challenges/facilitators in Article 5.3 policy implementation.

## Conclusions and the way forward

Our study demonstrates the promise of sub-national policies in line with the Article 5.3 of WHO-FCTC in visibly reducing tobacco industry interference in Indian states. However, there remain major challenges in the implementation of these policies.

Building on the perceived/recognized challenges and demonstrated good practices in implementing Article 5.3 policies so far, our study points to the way forward in optimising these policies for safeguarding effective tobacco control measures in Indian states. Developing clear implementation guidance, mobilising the empowered committees into functional planning and overseeing bodies, raising awareness about tobacco industry interference and the Article 5.3 policies among tobacco control stakeholders (especially, non-health agencies), and institutionalising complaint/redressal mechanisms for Article 5.3 policy violations as well as periodic active monitoring of tobacco industry interference incidents in states are crucial measures to strengthen implementation of Article 5.3 policies in Indian states. Correcting some of the design gaps in the existing state-level Article 5.3 policies and adopting a nationwide Article 5.3 policy would be crucial in preventing and/or reducing tobacco industry interference in tobacco-related public policies in India.

## Data and software availability

### Underlying data

This work draws on data from a brief questionnaire-based survey and focus group discussions. The survey data are all available in Tables 1 and 2. The FGD transcripts have not been made available in the public domain due to ethical concerns. FGD participants included nodal officers for tobacco control from health and other non-health departments from Indian states. Typically, each state had a very few staff manning the state tobacco control cell/committee with a single nodal officer for tobacco control at the state. Also, typically, non-health departments identify officers from their side who engage with the state tobacco control cell/committee. Hence, even mention of a designation or the name of the state carries the risk of identifying the participants. Tobacco control has been a very sensitive area riddled with conflicting interests within governments, commercial interests, and often tobacco control officers/advocates have faced repressive actions. Hence, we have not put the FGD transcripts in the public domain. We can make anonymised summaries from FGDs available to Editors/Reviewers if that helps in reviewing this paper. Others can write to the Ethics Committee at the Institute of Public Health Bengaluru (membersecretary@iphindia.org) requesting such access with justification.

### Extended data

The survey questionnaire and the FGD guide are provided under supporting information (S1 and S2 Files).

### Software availability

LibreOffice is a free software that provides similar functions to the proprietary software (MS Excel) used in this study.

## Supporting information

S1 File Questionnaire. (PDF)

S2 File Focus group discussion guide. (PDF)
