# Supplementary material for: Implementing the World Health Organization - Framework Convention on Tobacco Control Article 5.3: A qualitative study in 17 Indian states
Source: PLOS Glob Public Health. 2026 Jul 2;6(7):e0006522. doi: 10.1371/journal.pgph.0006522 (PMC13327259; doi:10.1371/journal.pgph.0006522)
Supplement: S1 File — (PDF) [file pgph.0006522.s001.pdf]

Supplementary File 1: Questionnaire

Self-reporting questionnaire

Part 1: Notification checklist assessment

S. No	Notification-Checklist	Response	Other Detail(s)
1	**Name of the state/UT**		
2	**Type/form of document**		
3	**Date of issue of the notification/order/gazette/circular/any other?**		
4	**Policy is issued by which department/authority?**		
5	**Is there a provision for constituting special/empowered Committee (EC) or any similar body**		
COMPLIANCE ASSESSMENT<br>If 5 is yes, then only proceed further:			
6	**As per the policy, is a Special/Empowered Committee (EC) or any similar body constituted?**		
6a.	**If 6 is yes, is the Empowered Committee (EC) functional?**		How many meetings/actions have been taken since its inception?
6b.	**If 6 is yes, is the Chairperson of EC appointed?**		If 6b is yes, please provide the designation and a date of appointment (or the time gap between policy adoption and appointment time)?
6c.	**If 6b is no, is there any person in-charge?**		If 6c is yes, then who and provide a date of appointment?
6d.	**If 6 is yes, are the other EC members appointed?**		If 6d is yes, please provide their approximate date of appointment?
8	**Is there an existing protocol for EC (fixing and conducting meetings with TI) to implement the Article 5.3 and to stop TII?**		
8a.	**If 8 is yes, has the protocol ever been followed since the policy is in place?**		If 8a is yes or no: Please describe achievement/challenges
9	**Is there any instance reported for violation of the Code of Conduct by the public servants (officials and employees)?**		
9a.	**If 6 is yes, was any action taken?**		If 9a is yes or no: Please describe achievement/challenges
10	**Is there any state/UT-level mandate (order) related to placing Article 5.3 display board in the government offices and/or institutions?**		
10a.	**If 10 is yes, then has it been implemented?**		If 10a is yes or no: Please describe achievement/challenges

Part 2: Policy Evaluation

A. Industry monitoring and other interference mitigation, after the state/UT-level policy adoption

IMPLEMENTATION ASSESSMENT (enclosed as a separate excel format)

Industry Monitoring and other Interference mitigation, after the state/UT-level policy inception																	
INCIDENT											OUTCOME						
SL. No.	Year	Month	Exact Date of	Description of what	Tobacco Industry (Name)	Type of Tobacco	Any Specific Brand	Any Front Group	MPOWER Category (if	TII Index Indicator	Was there any	Was it mitigated?	Exact Date of mitigation	Mitigated By?	Measures of Mitigation	Any other details	Evidence (Share link for media story/image)
1																	
2																	
3																	
4																	
5																	
6																	
7																	
8																	
9																	
10																	
11																	
12																	
