# Supplementary material for: Implementing the World Health Organization - Framework Convention on Tobacco Control Article 5.3: A qualitative study in 17 Indian states
Source: PLOS Glob Public Health. 2026 Jul 2;6(7):e0006522. doi: 10.1371/journal.pgph.0006522 (PMC13327259; doi:10.1371/journal.pgph.0006522)
Supplement: S2 File — (PDF) [file pgph.0006522.s002.pdf]

Supplementary File 2: FGD guide

Focus Group Discussion (FGD) guide

Setting: An in-person Focus Group Discussion (FGD) will take place in each of the identified state/UT, to be organised by the identified local person (Union state partner in most states). Prior day and time for the FGD is to be sought from the Focus Group (FG) and shared in advance with The Union in the 'FGD calendar'.

Focus group: Each FG will comprise of 6-8 members from:

- State Tobacco Control Cell (STCC):
  - ✓ State Nodal Officer (SNO) with at least 01 year of experience in tobacco control; if not, then former-SNO who worked in tobacco control,
  - ✓ Legal consultant,
  - ✓ State Consultant-NTCP / State program manager from NHM
  - ✓ A coalition member (if applicable)
  - ✓ Govt official from any department (other than health) etc –
- Academia (institute of excellence/medical/dental/social work/public health) involved in tobacco control within your state/UT
- Civil society organisation involved in tobacco control within your state/UT
- Any other government department involved in tobacco control within your state/UT

FGD will be facilitated by the identified local person (Union state partner in most states) in the presence (in-person or virtual) of staff from The Union.

Duration: 60-90 minutes.

Process: The staff member will moderate the discussion and provide some questions that link to areas we wish to cover in the focus group and serve to stimulate the discussion. And a note-taker will take notes, wherever necessary. The focus group session will be recorded over an audio device (phone recording or audio recorders if available) and an audio-video recording via the virtual platform employed (preferably zoom), which will facilitate later transcription.

Prior to the FGD, you may:

- Request the participants to carry along with them any government orders/letters/circulars documents/notifications/any document related to their state-level Article 5.3 policy guidelines. This might supplement the FGD with evidence
- discuss with the participants, what is 'tobacco industry', and 'tobacco industry interference' thereby spread a common understanding between participants
- FGD question themes: Approximately 22 questions under the following 5 sub-themes:

Sub-themes	Explanation
Stakeholders' Awareness Assessment	1. Please discuss in detail regarding the scope of the existing policy document: whole government/ select departments/ health only? Government/private stakeholders? Elected representatives or only employed staff?<br>2. Since the policy adoption, discuss the mechanism (if any) employed to share/disseminate it with other government departments (or individuals/agencies whom this protocol applies). (Clues: such as issuing D.O. letter etc)<br>3. Since the policy adoption, discuss the mechanism (if any) employed to share/disseminate it with all the districts. (Clues: such as any state advisory or any other directions to be followed by all the districts)<br>4. Since the policy adoption, please share (any evidence) if any public notice was issued to create any form of awareness among the general public.<br>5. Since the policy adoption, please explain (and share evidence) if there is any provision for display of a signage/board highlighting this policy on the government website and/or government offices or at offices of relevant agencies covered under it specifying prior permission and processes needed before interaction/contact between tobacco industry and respective government agency.

	6. Since the policy adoption, please share details if any SLCC meeting(s) have focused on monitoring and mitigating TIIs as an agenda point?<br>7. Since the policy adoption, please share if any SLCC meeting(s) have focused on implementation and/or compliance assessment of the policy, as an agenda point?<br>8. Since the policy adoption, please discuss and share any evidence if any state and/or district-level sensitisation workshop(s) have been organised or conducted by the government with respect to the FCTC Article 5.3 policy?<br>9. Since the policy adoption, please discuss and share any evidence if any state or district level sensitisation workshop(s) have been organised or conducted by any CSO/NGO with respect to the policy? **(For #2-#9: If yes, please provide details and share the document. If no, please specify any particular reasons/challenges)**
Challenges related to policy adoption and implementation	1. What challenges did you face before the 5.3 policy adoption?<br>2. What challenges did you face during the 5.3 policy adoption?<br>3. What challenges did you face after the 5.3 policy adoption?<br>4. What are some of the challenges you faced in implementing Article 5.3 policy. Can you give examples of the kind of challenges you faced?
Assessment of policy implementation and its effectiveness (here there ought to be a probe about the number of complaints received and resolved)	1. Which department (as per you) in your state/UT is more susceptible to TII?<br>2. What is the nature of the industry/agent involved in any kind of interactions with the government (trade body/lawyer/CEO etc.)?<br>3. Since the policy's adoption, is there a change in the occurrence of TII instances within your state? Please elaborate how. Explain the actions taken, if any.<br>4. Whether any complaint was received or any complaint taken up suo-moto under this policy? Number of complaints received, their nature and resolution details:<br>If yes, then:<br>a) What was the response generated by the departments/agencies in that regard?<br>b) What process was followed in resolving complaints?<br>c) whether a complainant is/was kept in communication about the process and outcomes?
Implementation mechanism	1. Other than the specified measures in your policy document, are there any specific strategies/best practices employed in your state for policy implementation (other than the enlisted measures in the policy document)?
Personal opinions and recommendations	1. Regarding the request of partnership and non-binding agreements with tobacco industries, will anyone (esp. government officials if involved) say no to big fat money donated by the tobacco industry in the garb of CSR?<br>2. Are there any notable conflicts of interest within your state for tobacco control? Please share in detail (any evidence related to the same)<br>3. What are your views on the bottom-up approach employed by India (in the absence of national policy on 5.3)? Please elaborate how.<br>4. In your opinion is the present policy on Article 5.3 in your state sufficient? What changes (policy content, implementation strategies) in your opinion in the existing policy will make it more robust?
