# Supplementary material for: Implementing the World Health Organization - Framework Convention on Tobacco Control Article 5.3: A qualitative study in 17 Indian states
Source: PLOS Glob Public Health. 2026 Jul 2;6(7):e0006522. doi: 10.1371/journal.pgph.0006522 (PMC13327259; doi:10.1371/journal.pgph.0006522)
Supplement: Attachment — Submitted filename: WHO FCTC Article 5.3_PGPH-D-25-02462_RG- Editor_20250919.pdf [file pgph.0006522.s003.pdf]

PLOS Global Public Health

Implementing the World Health Organization - Framework Convention on Tobacco Control Article 5.3: a qualitative study in 17 Indian states

--Manuscript Draft--

**Manuscript Number:**	PGPH-D-25-02462
**Article Type:**	Research Article
**Full Title:**	Implementing the World Health Organization - Framework Convention on Tobacco Control Article 5.3: a qualitative study in 17 Indian states
**Short Title:**	Implementing the WHO-FCTC
**Corresponding Author:**	Upendra Bhojani<br>Institute of Public Health Bengaluru<br>Bangalore, Karnataka INDIA
**Order of Authors:**	Upendra Bhojani, MPH, PhD
	Shivam Kapoor
	Amit Yadav
	Puneet Chahar
	Ashish K Pandey
	Rana J Singh
**Keywords:**	tobacco control; policy; implementation; tobacco industry interference; India; WHO FCTC; governance; conflict of interests
**Abstract:**	Introduction<br><br>Tobacco industry is known to influence tobacco-related public policies for commercial gains, often resulting in suboptimal tobacco control regulations and greater tobacco consumption rates. Recognizing this problem, the World Health Organization Framework Convention on Tobacco Control, a widely ratified United Nations treaty, in its Article 5.3 requires governments to protect their tobacco control policies from tobacco industry influence. Despite the global guidance on implementing Article 5.3, the national and sub-national implementation remains far from desired. India, one of the largest consumers and producers of tobacco in the world experiencing a high degree of tobacco industry interference, saw several states adopting policies in line with the Article 5.3. We study barriers and facilitators to implement these policies.<br><br>Methods<br><br>We studied 17 of the 19 states that had adopted 5.3 policies by 2023 that consented to be part of the study accounting for 64.3% (~999 million) of India's population. We started with a self-administered survey wherein a respondent from each state provided data about the nature and enforcement of 5.3 policies helping us to understand the context and identify relevant stakeholders for further in-depth study. We then conducted a focus group discussion in each state engaging purposively selected representatives from government departments and civil society. The inquiry focused on policy awareness/dissemination, barriers and facilitators in policy implementation, the perceived policy impact, and respondents' suggestions for improvements. 17 FGDs engaged a total of 258 participants. We used thematic content analysis to understand policy implementation processes.<br><br>Results<br><br>Major barriers to implementation of 5.3 policies included limited dissemination/awareness about 5.3 policies, suboptimal functioning of the committees constituted to oversee policy implementation, low priority accorded to these policies (by non-health departments) and interference by tobacco industry. Several government departments remain vulnerable to tobacco industry interference with the departments

Powered by Editorial Manager® and ProduXion Manager® from Aries Systems Corporation

	of education, health and municipal administration being the most vulnerable. A few states adopted practices that facilitated 5.3 policy implementation including display of policy signages and declarations, issuing of 5.3 policy orders by non-health departments, instituting channels to receive complaints about policy violations, and setting norms to not receive sponsorships by private entities in government departments. These policies were perceived to reduce visible interference from the tobacco industry and led some of the government agencies to refuse funding from and/or partnerships with the tobacco industry.<br><br>Conclusions<br><br>The sub-national 5.3 policies have a promise to reduce tobacco industry interference. There is need to address several systemic challenges and learn from a few positive practices from past to optimise implementation of these policies.
**Additional Information:**	
**Question**	**Response**
**Financial Disclosure**<br><br>Enter a financial disclosure statement that describes the sources of funding for the work included in this submission and the role the funder(s) played. This includes grants and any commercial funding of the work or authors.<br><br>This statement will be typeset if the manuscript is accepted for publication.<br><br>*Please review the submission guidelines and the instructions link below for detailed requirements and guidance.*	This work was not funded through any specific grants. Upendra Bhojani was supported for his time through the DBT/Wellcome Trust India Alliance (Clinical and Public Health) Senior fellowship [IA/CPHS/22/1/506533], and through an independent contract from the Vital Strategies (for data analysis and drafting of the report).
**Competing Interests**<br><br>On behalf of all authors, disclose any competing interests that could be perceived to bias this work.<br><br>This statement will be typeset if the manuscript is accepted for publication.<br><br>*Please review the instructions link below and PLOS Global Public Health's competing interests policy to determine what information must be disclosed at submission.*	Authors declare no competing interests

**Data Availability**<br><br>Before publication, Authors are required to make fully available and without restriction all data underlying their findings. Please see our PLOS Data Policy page for detailed information on this policy.<br><br>A **Data Availability Statement**, detailing where the data can be accessed, is required at first submission. Insert your Data Availability Statement in the box below.<br><br>Please see the data reporting section of our submission guidelines for instructions on what you need to include in your Data Availability Statement.<br><br>This statement will be typeset if the manuscript is accepted for publication.<br><br>*PLOS allows rare exemptions to address legal and ethical concerns. If you have legal or ethical restrictions, please use the box below to detail these in full sentences for the Journal team to consider.*	This work draws on data from a brief questionnaire-based survey and focus group discussions. The survey data are all available in the Table 1 and Table 2. The FGD transcripts have not been made available in public domain due to ethical concerns. FGD participants included nodal officers for tobacco control from health and other non-health departments from Indian states. Typically, each state had a very few staff manning the state tobacco control cell/committee with a single nodal officer for tobacco control at state. Also, typically, non-health departments identify officers from their side who engage with state tobacco control cell/committee. Hence, even mention of a designation or even name of the state carry the risk of identifying the respondents. Tobacco control has been a very sensitive area riddled with conflicting interest within governments, commercial interests and often tobacco control officers/advocates have faced repressive actions. Hence, we have not put the FGD transcripts in public domain. We can make anonymised summaries from FGDs available to Editors/Reviewers if that helps in reviewing this paper. Others can write to the Ethics Committee at the Institute of Public Health Bengaluru (membersecretary@iphindia.org) requesting such access with justification. For the same reason, we have anonymised the states while providing FGD excerpts and have rather used unique numbers for each state to help readers understand diversity of states being represented in the FGD excerpts reproduced in the result section of the manuscript.

Powered by Editorial Manager® and ProduXion Manager® from Aries Systems Corporation

Title

Implementing the World Health Organization - Framework Convention on Tobacco Control Article 5.3: a qualitative study in 17 Indian states

Short Title

Implementing the WHO-FCTC

Authors

Upendra Bhojani,[1]
Shivam Kapoor,[2]
Amit Yadav,[2]
Puneet Chahar,[2]
Ashish K. Pandey,[2]
Rana J Singh,[2]

[1] Centre for Commercial Determinants of Health, Institute of Public Health Bengaluru, Bengaluru, India.
[2] Tobacco Control Department, Vital Strategies, New Delhi, India

ABSTRACT

Introduction

Tobacco industry is known to influence tobacco-related public policies for commercial gains, often resulting in suboptimal tobacco control regulations and greater tobacco consumption rates. Recognizing this problem, the World Health Organization Framework Convention on Tobacco Control, a widely ratified United Nations treaty, in its Article 5.3 requires governments to protect their tobacco control policies from tobacco industry influence. Despite the global guidance on implementing Article 5.3, the national and sub-national implementation remains far from desired. India, one of the largest consumers and producers of tobacco in the world experiencing a high degree of tobacco industry interference, saw several states adopting policies in line with the Article 5.3. We study barriers and facilitators to implement these policies.

Methods

We studied 17 of the 19 states that had adopted 5.3 policies by 2023 that consented to be part of the study accounting for 64.3% (~999 million) of India's population. We started with a self-administered survey wherein a respondent from each state provided data about the nature and enforcement of 5.3 policies helping us to understand the context and identify relevant stakeholders for further in-depth study. We then conducted a focus group discussion in each state engaging purposively selected representatives from government departments and civil society. The inquiry focused on policy awareness/dissemination, barriers and facilitators in policy implementation, the perceived policy impact, and respondents' suggestions for improvements. 17 FGDs engaged a total of 258 participants. We used thematic content analysis to understand policy implementation processes.

Results

Major barriers to implementation of 5.3 policies included limited dissemination/awareness about 5.3 policies, suboptimal functioning of the committees constituted to oversee policy implementation, low priority accorded to these policies (by non-health departments) and interference by tobacco industry. Several government departments remain vulnerable to tobacco industry interference with the departments of education, health and municipal administration being the most vulnerable. A few states adopted practices that facilitated 5.3 policy implementation including display of policy signages and declarations, issuing of 5.3 policy orders by non-health departments, instituting channels to receive complaints about policy violations, and setting norms to not receive sponsorships by private entities in government departments. These policies were perceived to reduce visible interference from

the tobacco industry and led some of the government agencies to refuse funding from and/or partnerships with the tobacco industry.

Conclusions
The sub-national 5.3 policies have a promise to reduce tobacco industry interference. There is need to address several systemic challenges and learn from a few positive practices from past to optimise implementation of these policies.

INTRODUCTION

Tobacco industry is known to use a range of tactics to influence tobacco-related public policies for commercial gains from local to global level. In several countries, the high tobacco industry interference has been associated with suboptimal tobacco control measures and high adult smoking rates.(1) The World Health Organization Framework Convection on Tobacco Control (WHO-FCTC) recognizes such interference as problematic and, as part of its general obligations (Article 5.3), requires member governments to protect their public health policies related to tobacco control from commercial and vested interests of tobacco industry.(2) By the year 2020, about 88 countries had adopted one or more measures in line with the WHO-FCTC Article 5.3 implementation guidelines.(3)

While more countries ought to adopt policy measures to prevent tobacco industry interference, there remains a huge implementation gap in countries that have adopted such measures. The WHO-FCTC assessment, after a decade of its existence, demonstrated that while the convention was effective in reducing tobacco use prevalence when implemented, the Article 5.3 had remained one of the least implemented provisions of the treaty.(4) While there is no dearth of global guidance on implementing the WHO-FCTC Article 5.3 (3,5,6), the national and sub-national implementation remains far from desired. The Global Tobacco Industry Interference Index that assesses the implementation of the WHO-FCTC Article 5.3, in its latest report in the year 2023, revealed that none of the 80 countries surveyed were immune to tobacco industry interference.(7) In fact, more countries (n=43) showed deterioration in implementation of the WHO-FCTC Article 5.3 compared to countries (n=29) that showed improvement.(7)

We, therefore, turn the research gaze on implementing the WHO-FCTC Article 5.3. We focus on India that we believe is an ideal case for studying implementation, the lessons from which would be of relevance to many other countries. India is the second largest tobacco producer and exporter of unmanufactured tobacco with a huge domestic tobacco market. It is also home to the second largest number of tobacco consumers in the world after China. Further, reports shows that India experiences high degree of tobacco industry interference.(7–10)

Yet, in absence of a national policy in line with the WHO-FCTC Article 5.3, India championed a ‘bottom-up’ approach wherein starting with the Punjab in 2015, several states and at times the districts within states adopted policies to prevent tobacco industry interference.(11,12) By the year 2022 – the time this study was under conception – a total of 14 states and union territories (out of 36 such sub-national jurisdictions) in India adopted a policy in line with the WHO-FCTC Article 5.3 (now onward referred to as ‘5.3 policies’ in this paper).(13), Over time, more jurisdictions adopted such a policy, with 23 states by 2025.(14) Some improvements observed in implementation of the WHO-FCTC Article 5.3 in India over the recent years (2018-2021) have been in areas of minimizing unnecessary interactions between public officials and tobacco industry, avoidance of conflicts of interest for tobacco control among public agencies, and adoption of preventive measures – arguably, at least in part, linked to 5.3 policies at sub-national levels.(15)

Despite a decade since the first such sub-national policy got adopted in India, there have been a dearth of research about these 5.3 policies and the available studies have mainly focused on examining policy adoption, contents and compliance (15–20). In this paper, we aimed to study the facilitators and barriers at sub-national level in India during implementation of article 5.3 policies.

METHODS

Study approach

We conducted a qualitative study in 17 Indian states that have adopted 5.3 policy in some form using an initial self-administered survey followed by focus group discussions (FGD) with relevant stakeholders from governments and civil society. The purpose of the survey was to better understand the state level 5.3 policies and identify related stakeholders by seeking brief descriptions, more from a positivist perspective. The FGDs were meant to develop interpretivist accounts by the concerned

governments and civil society representatives and their experiences and perceptions with respect to implementing these policies at districts and state level.

Context

Based on the earlier documentation and our implicit knowledge, we identified 19 states and union territories in India having 5.3 policies in some form. Barring two of the states, where we could not secure administrative approval in time, we conducted the study in 17 states and union territories: (1) Rajasthan, (2) Uttarakhand, (3) Maharashtra, (4) Uttar Pradesh, (5) Bihar, (6) Kerala, (7) Karnataka, (8) Chhattisgarh, (9) Jammu & Kashmir, (10) Assam, (11) Punjab, (12) Nagaland, (13) Puducherry, (14) Jharkhand, (15) Meghalaya, (16) Tamil Nadu, (17) Madhya Pradesh. These states account for about 64.3% (about 909 million in the year 2025) of India's population. For the purpose of this paper we will use 'state' as a generic term to refer to each of these sub-national units even if a few of these (Puducherry, Jammu & Kashmir) are union territories at the time of the study i.e., administrative units governed directly by the central/national government.

Sampling, data collection, and analysis

While we describe each method separately, the tools (questionnaire, and the FGD guide) used for both were refined following (1) a validation exercise (22,23), wherein a panel of experts were asked to score/comment on face validity (importance of the questions) and content validity (relevance, clarity, and completeness of the questions); and (2) field pilots in two of the non-study states.

Survey

We sent out an online self-administered questionnaire as a Google Form. Respondents were representatives from civil society organizations working in tobacco control in study states. The questionnaire had two parts: one enquiring about the 5.3 policy (issuing authority, scope, formation and functioning of overseeing/implementing body, and reporting of violations) and the other about the complaints received and action taken (if any). Respondents filled in the questionnaire in close consultation with the officials from the respective state tobacco control cell with whom they had a close working relationship. This process was completed between May 23 (2023) and November 7 (2023). Data generated through questionnaire forms were downloaded in MS Excel format. We analyzed the data (with mostly yes/no or short descriptive responses) generating brief 5.3 policy descriptive profiles of each study states. These findings helped us understand the context and identify important stakeholders for FGDs.

FGDs

We conducted a total of 17 FGDs, one in each study states. The principal investigator (SK) and a few of co-investigators (PC, GB, AY) along with the facilitators (representatives of the TCD's partner organizations) in each state facilitated the conduct of FGDs. TCD's partner organization were trained/calibrated through an (online) workshop about the purpose of conducting FGDs, the FGD guide, and the process. These facilitators helped in purposive sampling of respondents ensuring representation from the state tobacco control cells of health departments, other health department divisions (e.g., food safety, mental health, non-communicable diseases, oral health) as well as representatives from the non-health departments of state governments (e.g., education, municipal corporations, tourism etc.) and selected civil society entities including non-government organizations (NGOs) and academic institutions that were active in tobacco control in the state. The participants were invited to an in-person FGD.

The investigators/facilitators briefed the participants about the tobacco industry interference and the WHO-FCTC Article 5.3. They summarized to them the survey findings (about 5.3 policy) for that state inviting feedback on any updates/corrections. The facilitators then initiated and moderated the discussion by bringing in lead questions from the FGD guide that were related to understanding awareness among stakeholders about the policy (scope, contents, dissemination, sensitization, and capacity-building activities), challenges faced in adopting and implementing the policy,

implementation mechanisms, perceived compliance with the policy and its impact, and respondents' suggestions for enhancing the implementation of these policies.

The FGDs lasted for an average of 90 minutes (40-130 minutes) and 10 of the 17 were audio recorded. For the rest, we took detailed notes as we either did not get the consent for audio-recording (n=2) or the venue was too large rendering the recording ineffective (n=5). The audio recordings and handwritten notes were transcribed and translated to English (where needed). These FGDs were completed between June 12 (2024) and October 13 (2024).

For FGD, MS Excel was used to organize the transcripts. The data was coded based on pre-determined inquiry probes (i.e., awareness, challenges in implementation, implementation mechanisms, and suggestions for improvements) and used thematic content analysis to identify major themes within these areas. Group dynamics and corroborated data was considered, where possible, from responses of participants across sectors (governments, civil society) and across positions within and across the government departments.

Ethics

The study was formally reviewed and approved by the Ethics Advisory Group at The Union (Ref: 07/2022) and the Institutional Ethics Committee at the Institute of Public Health Bengaluru (Ref: IPH/23-24/E/2). For both, the survey and the FGDs, written informed consents were sought from respondents after briefing them about the study and the rights of the respondents.

RESULTS

Characteristics of the survey respondents

The survey respondents, one each from 17 states, included 10 NGO representatives working closely with state governments, four state government representatives, and three consultants of the TCD seconded to the respective state governments. These included 12 men, and five women well versed with the 5.3 policies in their respective states.

Characteristics of the FGD participants

In the 17 FGDs, a total of 258 participants comprising 181 men (70.2%) and 77 women (29.8%) participated. Government representatives accounted for 67% (n=173) of respondents and came from 16 departments with a majority from health department followed by education department and a few respondents from a range of other departments (food & drug, police, youth development/affairs, municipal/urban development, tax/customs, labor, tourism, agriculture/horticulture, information & communication, transport, child protection, alcohol prohibition, law, legal metrology, and consumer affairs). Civil society representatives (n=85, 33%) mainly came from NGOs and academia (including medical/dental educational institutions and associated hospitals) with a very few journalists and an elected leader. See Fig 1 for sector- and gender-based distribution of FGD respondents. Typically, each state had a very few staff manning the state tobacco control cell/committee with a single nodal officer for tobacco control at state. Also, typically, non-health departments identify officers from their side who engage with state tobacco control cell/committee. Hence, naming the state while quoting the excerpts from FGDs carry the risk of identifying the respondents. Hence, we have anonymized the states while providing FGD excerpts and have rather used unique numbers for each state to help readers understand diversity of states being represented in the FGD excerpts reproduced in this section.

Fig 1 Characteristics of FGD participants

[Fig 1 provides distribution of FGD participants as per their gender and the sector (government, civil society) they worked in.]

About 5.3 Policies in Indian states

Punjab was the first state to adopt a 5.3 policy in July 2015 and Nagaland the latest within the study period notifying the 5.3 policy in June 2023. In all the states the policy was issued by a senior officer from the health department except in Chhattisgarh where the 5.3 policy was issued by the state nodal officer for tobacco control. See Table 1 for the details on attributes of the 5.3 policies issued by the states as gathered from the survey.

Table 1 Features of the 5.3 policies issued by the states in India

**States**	**Type of the (5.3 policy) document**	**Policy issue date (DD/MM/YYYY)**	**Policy issuing office**	**Is policy applicable to non-health departments?**	**Is there a provision in the policy for constituting a body to oversee implementation?**	**Whether such a body overswing the policy implementation constituted?**	**Whether such a body overseeing the policy implementation active or operational?**
Punjab	Notification	13/07/2015	Principal Secretary (Health Department)	Yes	Yes	Yes	Yes
Bihar	Notification	23/06/2017	Special Secretary (Health Department)	Yes	Yes	Yes	No
Jammu & Kashmir	Circular	27/07/2017	Additional Secretary (Health and Medical Education Department)	Yes	Yes	No response	No response
Tamil Nadu	Order	10/11/2017	Principal Secretary (Health Department)	Yes	Yes	Yes	No
Jharkhand	Notification	01/10/2018	Principal Secretary (Health, Medical Education & Family Welfare Department)	Yes	Yes	Yes	Yes
Karnataka	Notification	25/01/2019	Under Secretary (Health & Family Welfare Department)	Yes	Yes	Yes	Yes
Kerala	Order	30/04/2019	Additional Chief Secretary (Health & Family Welfare Department)	Yes	Yes	Yes	Yes
Meghalaya	Order	26/06/2019	Secretary (Health & Family Welfare Department)	Yes	Yes	Yes	Yes
Uttar Pradesh	Notification	16/09/2019	Secretary (Health & Family Welfare Department)	Yes	Yes	Yes	Yes
Chhattisgarh	Letter	02/02/2021	State Nodal Officer (National Tobacco Control Program)	No	No	Not Applicable	Not Applicable
Rajasthan	Circular	28/10/2021	Secretary (Medical Health & Family Welfare Department)	Yes	No	Not Applicable	Not Applicable
Puducherry	Order	27/12/2021	Chief Secretariat (Health Department)	Yes	Yes	Yes	No
Madhya Pradesh	Order	13/01/2022	Director (National Health Mission)	No	No	Not Applicable	Not Applicable
Uttarakhand	Order	05/05/2022	Secretary (Health & Medical Education Department)	Yes	Yes	Yes	No
Maharashtra	Notification	18/07/2022	Additional Secretary (Public Health Department)	Yes	No	Not Applicable	Not Applicable
Assam	Order	09/03/2023	Principal Secretary (Health & Family Welfare Department)	Yes	Yes	Yes	No
Nagaland	Notification	16/06/2023	Commissioner & Secretary (Health and Family Welfare Department)	Yes	Yes	Yes	Yes

Some of the design features within 5.3 policies limited their implementation. Six of the 17 states had no provision for creating a body that steers and/or oversees policy implementation (Table-1). While 10 out of the remaining 11 states had constituted an overseeing body (routinely called, an ‘empowered committee’), only six such bodies were functioning i.e., the committee had convened meetings, deliberated about 5.3 policy issues and/or taken actions. Furthermore, in two of the 17 states studied (Chhattisgarh, Madhya Pradesh), the 5.3 policies issued by health departments were mostly applicable within health departments (or excluded non-health departments) limiting their scope in addressing tobacco industry interference.

Some of the FGD respondents opined that ideally, a policy at the state level ought to be issued through a cabinet approval and published in the state gazette, or at least by a senior/top bureaucrat within the health department else it might not command authority/compliance from non-health departments.

“We faced challenges in convincing superiors for the need to issue a policy…So, we issued it, but we realized that at district level, district collectors are not giving any importance to the policy as it is not issued by the department head” (FGD-10)

“It (5.3 policy) isn’t a policy document… It is not issued through cabinet approval…it is an executive order at the state level.” (FGD-13)

Challenges in Policy Adoption

Not many respondents across states commented on challenges faced in the adoption of the 5.3 policy. This could also be partly because most of them were not involved in policymaking. Nonetheless, they highlighted two major challenges they faced in facilitating adoption of 5.3 policies.

Government representatives from at least seven states indicated that they found it difficult to convince the high-level officials and decision-makers of the importance of 5.3 policies, especially in contexts of its scope to include non-health departments and elected representatives.

“Challenge is to make policymakers (elected representatives) understand why it is important to have such a policy (5.3 policy). With efforts, they understand some of the components of a policy proposal but not all. Government officials seem more convinced and hence they have issued orders. It is seen as a very sensitive issue by policy makers…some (of the policymakers) feel that anyone can meet anyone in democracy (this is in reference to the policy preventing meetings between public agencies and tobacco industry representatives unless for regulatory reasons).” (FGD-13)

“I faced challenges in convicting my superiors for the need to issue a policy in line with the WHO FCTC Article 5.3. They kept saying you better issue (a letter regarding such a provision) at your level and raise awareness first, and we shall discuss about the notification later.” (FGD-10)

A few respondents believed that having conflicting interests in tobacco by governments and/or not having a substantive presence of the tobacco industry in their states (cultivation, manufacturing) made it even more difficult to convince decision-makers on the need for 5.3 policies.

“They often feel why such a policy when there is no cultivation of tobacco and tobacco industry in the state.” (FGD-12)

Respondents from at least four states indicated a delay of up to two to three years in moving the file (regarding the policy proposal) within various offices of the government.

“It took long time, nearly three years, with files shifting many tables before the policy got adopted.” (FGD-16)

Challenges in policy implementation

In this section, we describe the major themes defining challenges in implementing 5.3 policies in states.

Low awareness regarding 5.3 policy

On the whole awareness about 5.3 policies was limited among government representatives. In the nine of 17 states studied, the awareness about the presence of 5.3 policies was mainly limited to health department officials and at times including officers from education and food safety (and drug control) departments. In one of the states, a few health officers also did not know about the policy in their state.

"I am not aware…May be the circular was disseminated directly from health secretary to the district heads not keeping us in loop…" (FGD-11)

In the rest of the six states nearly all the respondents representing various government departments were aware of the presence of 5.3 policy in their state. However, even in these states, the detailed knowledge about various provisions in these policies was typically limited to health department officers.

Representatives of the NGOs that participated were aware of the 5.3 policy. However, many of the representatives from academia (mainly medical/dental colleges) were unaware of the 5.3 policy adoption by their respective states even though some of them were aware of Article 5.3 in the global treaty (i.e., the WHO FCTC). Exceptions included academics attached to cancer institutes/hospitals, who were actively engaged in tobacco control research/programs.

"It (5.3 policy) is not sent to us." "I have no idea of such a policy (5.3 policy) in Uttarakhand. I have not received a copy of such policy…It (5.3 policy) should be circulated to all the organizations, all the academic institutions." (FGD-14)

Limited dissemination of 5.3 policies within states

Typically, 5.3 policies issued at the state level were sent to the office of district administration (referred variably as district magistrate, commissioner, or collector) with an expectation that the district administration would further disseminate the policy to various department heads in respective districts. However, this was not always the case. For example, in one of the states, the 5.3 policy was not sent to districts as state level officers awaited the first meeting of the state level empowered committee to happen, which kept getting delayed.

"For now, the (5.3 policy) copy has been issued to all EC (Empowered Committee) members. After the first EC meeting, NTCP (national tobacco control program) will write letters to districts (regarding 5.3 policy)." (FGD-17)

"The order (5.3 policy) was sent to all the district magistrates for further action. But only five district magistrates sent it to other departments in their respective districts. This gap needs to be solved by sending the order again to all district magistrates and directing them to send to other departments." (FGD-7)

In four of the 17 states, the state level coordination committee on tobacco control (a body under the national tobacco control program) had discussed 5.3 policy in its agenda for one or more times focusing on its adoption, dissemination, and/or enforcement. In one of the states, the high-powered committee on tobacco control (chaired by the chief secretary) had discussed 5.3 policy as part of its agenda items. None of the states studied had issued a public notice or undertook measures (e.g., mass media use) to raise public awareness about the 5.3 policy.

Sub-optimal functioning of empowered committees

Typically, 5.3 policies prescribe the constitution of an empowered committee that oversees the implementation of these policies. Respondents from certain states specifically mentioned that these committees, while constituted in these states, have not met since their constitution. Such was likely a case for many more states. Some respondents believed that the lack of an active and functioning committee was a basic hurdle that hindered state tobacco control cells from taking further steps needed for implementing 5.3 policies.

"No specific meeting has happened of the WHO FCTC Article 5.3 state level empowered committee. If such review meetings happen regularly, senior officials will get sensitized about this policy." (FGD-6)

"The empowered committee under the policy has not met even once since its formation." (FGD-3)

Lack of clear guidance for implementation

Respondents from many states highlighted that 5.3 policies in their present shape lacked clear guidance on many operational aspects concerning implementation: (1) what constitutes the tobacco industry? Is it just manufacturers and vendors or others linked directly and indirectly to the tobacco industry? (2) whom to report violations of the policy provisions? (3) what steps are to be taken when a complaint is received, or a policy violation recognized? (4) what are the precise roles and responsibilities of empowered committees constituted under 5.3 policies?

"There is a lack of clarity…among the government officials as there are no clear guidelines what must be done. Also, we don't know what constitutes the tobacco industry, whether the manufacturers, or retailers or wholesalers." (FGD-8)

Low priority accorded to 5.3 policy implementation

Respondents highlighted that often non-health departments accorded low priority to implementing 5.3 policies as they either underappreciated the problematic nature of tobacco industry interference or were occupied with their department-specific priorities. The turnover of staff within other departments was also perceived as a barrier, especially as there was no system for periodic sensitization/training about 5.3 policy.

"There is a general lack of awareness about tobacco industry interference in other (non-health) departments…they give low priority to tobacco control as they all have their own priorities…There has been much movement of staff in police department, so those trained in tobacco control often moved to other departments." (FGD-12)

At times, 5.3 policy implementation agenda got delayed in face of the changes in high-level administrative and political leadership, and other pressing priorities (such as the COVID19 pandemic) for health departments.

"COVID19 pandemic that started after adopting the policy seem to have sidelines the policy and its implementation agenda." (FGD-7)

"The first EC (Empowered Committee) meeting was to be held right after the (issue of the) notification (5.3 policy). It got delayed due to change in leadership at Secretariat level." (FGD-17)

Tobacco industry interference within 5.3 policy implementation

Respondents from two states reported that while the adoption of the 5.3 policy in the state reduced and/or stopped the tobacco industry's overt outreach to senior health officials, the industry was now engaging directly with lower level (implementation) officials, at times in the disguise of non-government organizations or as the industry front groups.

"…for example, the industry representative approached municipal corporation officer who had recently taken charge (newly joined the position) and requested for a meeting with enforcement officers to discuss the point-of-sale advertisements." (FGD-5)

Respondents from one of the states flagged that tobacco industry representatives often approached political leaders to bypass senior department officials initiating new partnerships with government agencies.

"Tobacco Industry representatives are often approaching elected political leaders directly (bypassing the senior bureaucrats) about tobacco regulations." (FGD-6)

Respondents from 13 out of the 17 states studied mentioned specific government departments that they felt were particularly vulnerable to industry interference. Such risk perception was based on the incidence of the tobacco industry interference in the past as well as the potential that certain departments offered to the industry to reach children/youth as potential consumers through its so-called corporate social responsibility activities/partnerships. The education department was perceived to be the most vulnerable followed by the health and the municipal administration departments. Fig 2 depicts the perceived risk of the industry interreference about various government departments.

Fig 2 Perceived vulnerability of government agencies for tobacco industry interference
[Percentages in Fig 2 refer to the frequency of times that state was mentioned as vulnerable to tobacco industry interference by FGD respondents.]

Respondents from many states mentioned how the lack of real-time monitoring of tobacco industry interference in states makes it difficult to implement and evaluate 5.3 policies. Many within state tobacco control cells and civil society sense the tobacco industry interference but the lack of systematic monitoring and documentation hinders corrective actions.

Good practices in implementation of 5.3 policy
In this section, we highlight practices within Indian states that facilitated 5.3 policy implementation despite challenges described in the earlier section.

Display of 5.3 policy and related signages
A few states incorporated the provision of displaying signages (information boards) at government offices as part of the 5.3 policies and implemented this provision to varying degrees. This was perceived to be a useful practice in raising awareness about 5.3 policies within government departments and those visiting these offices. Respondents from at least three of the 17 states mentioned about display of such signages, mentioning the need for prior permission/procedure to be followed by the tobacco industry in meeting government officials, at some or many of the government officers at state and district level.

"The policy is disseminated through the advisory issued to all the district heads (of departments) for prominently displaying the FCTC (5.3 policy) signages in all government offices…the display boards are prominently put up at government offices and institutions." (FGD-5)

"Signage boards highlighting the (5.3) policy are present in government offices, which are visible to all and public." (FGD-9)

Though not mandated by 5.3 policies, some of the government and civil society representatives from two of the states reported to have displayed 5.3 policy and/or a declaration about them not partnering with or taking funds from the tobacco industry (this one specifically in case of NGOs and academic institutions) on their organizational websites.

"To uphold transparency, some of the organizations in the state have displayed a conflict of interest declaration on their websites. This visual representation underlines the commitment to transparency… setting clear expectations regarding the processes required before any interaction between the tobacco industry and government agencies." (FGD-16)

"No signage is being shared by any office, but it (5.3 policy) is displayed on government department website." "It (5.3 policy) is displayed on XXX (civil society organization) official website." (FGD-15)

Issue of 5.3 policy orders by non-health departments

In a few states, the issue of 5.3 policies by the health department followed the issue of similar orders/notifications by selecting non-health departments enhancing awareness about and compliance with these policies within these departments. Typically, departments in the education sector issued such orders/notifications as mentioned by respondents from two of the states.

"A comprehensive order (about 5.3 policy) has been issued by the Department of School Education and Literacy Development as well as the Department of Higher and Technical Education, to check tobacco industry interference in educational institutions in any form." (FGD-5)

"So, at state level, there have been two orders (about 5.3 policy). The Director NHM (National Health Mission) released an order… Secondly, XXX, who was additional commissioner in higher education released an order about 5.3 policy." (FGD-13)

Attempts at monitoring tobacco industry interference

While there seems to be a lack of systemic monitoring of tobacco industry interference by governments, three of the states reported some progress in this direction. One of the states designated state and district-level tobacco control committees to also serve as monitoring committees for the implementation of 5.3 policies. Two of the states reported the use of WhatsApp group among health department officials and a phone-based helpline (for seeking medical help) as sources of receiving complaints and/or intelligence about tobacco industry interference in these states respectively.

"Monitoring information (about tobacco industry interreference) often comes from district level officials and WhatsApp groups." (FGD-9)

"Complaints have been received under 5.3 (policy) through the medical helpline number (104) and actions are being taken." (FGD-4)

Normative measures in line with the WHO FCTC Article 5.3

The respondents highlighted certain normative measures taken by health and non-health departments in a few states that were in line with the WHO FCTC Article 5.3 and helped enhance compliance with 5.3 policy. In one of the states, health department officials set a norm (not formally provided for in the 5.3 policy) that tobacco industry representatives wanting to engage with the health department need to first engage with the high-level offices (typically, health secretary and/or director of the national health mission) necessarily. This arrangement was in anticipation of the focused and better implementation of 5.3 policies. In another state, in lieu of a long-pending meeting of the state-level coordination committee, the agenda related to the 5.3 policy was put to the high-powered committee on tobacco control chaired by the chief secretary established in the state. This helped overcome the status quo on 5.3 policy implementation. The police department in one of the states seem to have adopted rules preventing sponsorships from any private organizations (and hence, also from tobacco industry).

"The PS (Principal Secretary) and MD (Managing Director) of NHM (National Health Mission) are the designated points of communication for the tobacco industry. The representatives of the tobacco industry cannot meet with just anyone and must adhere to these specific channels." (FGD-9)

"We (police department) do not have 5.3 policy as such…However, a general awareness has been created among department officials on not to use tobacco, tobacco smoking or spitting. In the department, we have rules about not to take any sponsorship from private organizations (this response was in relation to the discussion about sponsorship to public agencies by tobacco industry)." (FGD-10)

Perceived Impact of 5.3 Policies

This section briefly outlines respondents' perceived impact of 5.3 policies on reducing tobacco industry interreference in states. Seven of the 19 states studied reported to have received complaints about violation of the 5.3 policy. These complaints were typically about tobacco industry entities partnering with, or providing funds to, government agencies, often as part of their so-called corporate social responsibility. These states received a very few – just one or two – complaints, except Tamil Nadu and Uttar Pradesh that received more complaints (eight to ten). Typically, the response from the state governments took the form of the state-level coordination committee for tobacco control issuing a letter to the concerned government agency requesting it to dissociate itself from tobacco industry. At times, the committee member/s or the officer from the state/district tobacco control cell visited the concerned government agency to explain the matter. Such actions typically resulted in termination of, or at least no further expansion of such tobacco industry partnership/funding. Table 2 provides a brief qualitative account of complaints (and hence, the nature of the violations under the 5.3 policy) and resolutions for each of these seven states.

Table 2 A brief account of complaints under 5.3 policies and their resolutions in select Indian states

States	Policy violation and/or action
Jammu & Kashmir	In a few of the districts, district administration and police received sanitizers and protective gears from the tobacco industry during the COVID-19 pandemic. This was publicized in print media. The state tobacco control cell wrote a letter to the Director (health department), who in turn wrote to all the districts (administration) advising against any such future funding/partnerships.
Maharashtra	The state received complaints about a medical college, a general hospital, and some schools receiving funding through the so-called CSR activities by the tobacco industry. State issued letters to respective authorities including the medical education department and concerned district nodal officers. At times, the nodal officers and/or district committee member/s made visits to the government agency in question. This resulted in termination of such funding/partnerships.
Assam	There was probably no formal complaint, but the state tobacco control cell took notice of the support being received by the social welfare department from the tobacco industry for a nutrition-related program. Subsequently, a letter was sent from the health department to the social welfare department highlighting the 5.3 policy. It was unclear what was the impact of such a letter on this specific funding/partnership.
Karnataka	There were several complaint-based and/or Suo-motu actions about government agencies receiving funds/partnerships from the tobacco industry (e.g., tobacco industry partnering with municipal agencies for solid waste management; schools receiving funds from the tobacco industry for amenities; tobacco industry partnering with watershed development department). Actions – mainly in the form of letters /notices from health and/or education departments – resulted in the termination of funding/partnerships in most, but not all, such instances. The state tobacco control cell alerted higher officials in the health department whom the tobacco industry was approaching with a proposal to build a hospital in public-private-partnership mode. Subsequently, such a proposal did not materialize.
Tamil Nadu	There were complaints received through the telephone-based medical helpline and the state reported to have taken actions. A specific example was of the tobacco industry supporting a 'spell-bee competition' among government schools. A letter to schools (by education department) stopped such partnerships.

Meghalaya	There was probably not a formal complaint, but the state tobacco control cell came to know that the tobacco industry was approaching a university to provide research support/funding. The state tobacco control cell reached out to that university and was able to prevent the funding/partnership.
Uttarakhand	The state received a complaint about the tobacco industry supporting an educational institution in the state. The state tobacco control cell intervened and stopped the funding/partnership.
Uttar Pradesh	The state received eight to ten complaints, all of which were related to government agencies receiving funds/partnerships/benefits/unnecessary interactions under the so-called CSR initiatives of tobacco industry. Except for two of these complaints, the rest were resolved. The actions were mainly in the form of letters /notices from the administration to the government agency in question and resulted in the ending/dilution (removal of industry logos) of funding/partnerships in most, but not all. The unresolved complaints included tobacco advertisements on public transport buses as the prevailing regulations did not provide clarity on the removal of such advertisements.

Apart from complaints-based actions, respondents articulated other forms of perceived impact of this policy. In one of the states, the civil society representative felt major tobacco companies stopped publicizing their so-called corporate social responsivity activities once the state adopted 5.3 policy. However, this may not necessarily mean that they stopped doing these activities.

"After the (5.3) policy, major cigarette companies in the state (names a couple of them) stopped publicizing their CSR activities." (FGD-11)

In another state, a civil society representative mentioned how some of the high-level government officials across departments (especially, health and police) had refused an offer of partnership by the tobacco industry (as part of its so-called CSR initiative) indicating a positive impact of the 5.3 policy. In one more state, a civil society representative working closely with the state tobacco control cell indicated that tobacco industry representatives used to visit the state tobacco control cell and other high-level officials from the health department on a regular basis. Such visible outreach by the tobacco industry to health officials stopped after the 5.3 policy adoption by the state.

"While it is difficult to say about the impact of the 5.3 orders in concrete terms but there have been instances where higher officials have refused CSR from tobacco companies, which is a positive indicator of the impact of the 5.3 orders." (FGD-13)

Importantly, many respondents across states mentioned how difficult it is to comment about the impact of the policy as there was no real-time monitoring of tobacco industry interference in states.

"As there is no active monitoring and awareness (about tobacco industry interference), no complaints are registered/received under this (5.3) policy. Other (non-health) departments are not implementing it, not prioritizing it." (FGD-12)

DISCUSSION

We studied implementation of 5.3 policies in 17 Indian states through a self-administered survey and FGDs involving concerned government and civil society representatives. We identified several barriers impending effective implementation of these policies including, limited dissemination of and awareness about these policies (especially at district level and among non-health department officials), suboptimal functioning of the empowered committees constituted to oversee policy implementation, relatively low priority accorded to these policies (especially by non-health departments) and interference by tobacco industry. The respondents identified several government departments that remain vulnerable to tobacco industry interference, with the departments of education, health and municipal administration being most vulnerable in that order. We also identified practices by a few states that are facilitating 5.3 policy implementation including public display of physical signages and web-based declarations related to 5.3 policy, issuing of 5.3 policy orders by

non-health departments, instituting channels (WhatsApp groups, telephone-based health helpline) to receive complaints about the 5.3 policy violations, gatekeeping of industry outreach to government agencies by senior officials, and adopting a norm to not take sponsorships by private entities in government departments.

The promise of bottom-up regulatory initiatives

Overall, our findings imply that despite some years (2-10 years) of having 5.3 policies in place, their implementation remains very limited. However, despite the limited implementation, the perceived positive impact of these policies in the form of visible reduction in industry-government interaction and some instances of refusals by government agencies to CSR-based partnership offers of tobacco industry indicate the importance of these policies. In fact, these bottom-up district- and state-level regulations, in absence of a national (all-of-the-government) level 5.3 policy, are not just important but also promising in terms of the incremental increase in their coverage over time. Bassi et al(18) had identified and analyzed 13 states that had adopted 5.3 policies by 2019. Our study could identify 19 states with state level 5.3 policy in some form by 2023. More recently, this number has gone up to 23 by the mid 2024.(24) This phenomenon is not isolated in Indian context as many of the tobacco control regulations in India were initiated at state level (a few even at district level) before they were enacted at national level (e.g., prohibition of electronic nicotine delivery systems(25)) or adopted by most or all of the sub-national jurisdictions across India (e.g., prohibition on certain forms of smokeless tobacco(26); prohibition on sale of single/loose cigarettes(27)). In a global tobacco control context, where the adoption of policies in line with the WHO FCTC Article 5.3 remains limited, the Indian experience indicates promise of bottom-up regulations in federal democracies.

We now discuss our findings in relation to a few relevant studies done in the past and highlight the major implications on enhancing the implementation of these policies in Indian states.

Plugging design gaps hindering implementation

The limited number of studies that have analyzed 5.3 policies in Indian states have mainly focused on their contents highlighting several gaps in these policies vis-à-vis the provisions in the implementation guidelines for the Article 5.3 of the WHO FCTC.(18,24) While these gaps represent weaknesses in design of these policies, our study especially points to the gaps – not defining tobacco industry, the role of empowered committee, the complaint and time-bound resolution procedures including punitive actions – are hindering the implementation of the prevailing 5.3 policies. Hence, either through amendments to the existing policies or through the notification of rules under the existing policies, there is an imminent need to address these gaps and make these policies enforceable.

Raising awareness about 5.3 policies

When it comes to implementing the existing 5.3 policies, the persistent challenge has been the very limited awareness of these policies among government officials and stakeholders, especially so among officials in non-health departments and at district/sub-district levels.(18) This is primarily explained by poor dissemination of policy from state to district levels and to various heads of non-health departments through established administrative channels. There is need to periodically issue circulars about 5.3 policies from state health department to district offices, ideally complemented by issue of such circulars by each non-health departments ensuring vertical dissemination within departments. What will especially help this endeavor is if such circulars require display of the signages about 5.3 policies at conspicuous places within government offices, display of 5.3 policy on departmental websites, and periodic sensitization events to raise awareness about these policies within government offices. Additional efforts at raising awareness among civil society organizations and public at large are also crucial to build demand for enforcement of these policies. This implies the need for public-facing awareness campaigns using mass media (including public notices in prominent news dailies) and sensitizing concerned civil society actors (especially academia, health-related NGOs, and journalists).

Advocating for implementation processes

Our study points to the need to turn the gaze on implementation processes for 5.3 policies in Indian states. There is need to push for the clarity on roles and functioning of the empowered committees as in most states, these committees have not met once since their constitution. In this context, some of the practices adopted by states in implementing 5.3 policies indicate the importance of tailoring to and institutionalizing 5.3 policy implementation within prevailing norms/practices of specific government departments rather than one-size-fits all approach e.g., issuing of notification/circulars by non-health departments about 5.3 policy; utilizing WhatsApp group of (tobacco control) program-specific staff to share intelligence about tobacco industry interference; or utilizing existing telephone-based health helpline or existing tobacco monitor apps on phones for receiving complaints regarding violations of 5.3 policies. Furthermore, having a national policy in line with the WHO FCTC Article 5.3 and integrating it into processes of the National Tobacco Control Program is likely to enhance implementation and monitoring/review of 5.3 policy implementation.

Monitoring tobacco industry interference

Many respondents in our study pointed to the lack of any functional institutional system for real-time and/or periodic monitoring of tobacco industry interference. Civil society organizations in India have produced the tobacco industry interference index at national level on an annual basis from 2018. While this exercise itself remains voluntary and ad hoc (the last index we could locate was for 2023), it indicates a high level of tobacco industry interference in tobacco control. In 2023, India was ranked at 40th place in a survey of 90 countries in terms of implementation of the WHO FCTC Article 5.3 with an index score of 58 out of 100 (higher the score, greater the industry influence). Such exercise is rare at state (sub-national) level.(28) A few such attempts at sub-national level indicates that it is feasible and important to monitor tobacco industry interference at state level as such influence remain high and variable across states.(9,19) More importantly, such exercise ought to be institutionalized to ensure its sustenance and linkage with policy/practice response. As attempted by one of the states under this study, such monitoring could be done through existing committees or their sub-groups (such as, state- or district-level coordination committees for tobacco control constituted under the National Tobacco Control Program) and ideally include experts from academia and civil society. Such a forum shall bring out periodic reports and provide intelligence to the empowered committee under the 5.3 policy for action.

Limitation

While the embeddedness of this study – wherein respondents included government officers across departments with leadership and/or professionally mandated roles for tobacco control – is its strength, it came at the cost of rigor in data collection for this study. It was not easy to convene the FGDs, especially getting the government representatives given their hectic work schedules and competing priorities. Hence, 12 of the 17 FGDs had to be conducted at the beginning or at the end of other meetings to ensure participation of the key government representatives, who had committed to participate in those meetings. This, at times, led to some participants who were not the primary target of our FGDs, to joining the FGD alongside their senior officials. They mostly remained silent participants. The hierarchical pattern of reporting and relationships within and across government departments impacted the free and candid expression by participants, especially when senior bureaucrats (secretaries, commissioners, directors) participated. The same could have been the case for a few of the civil society representatives, whose work demanded close working with and/or funding from state governments. Hence, there was a blurring of lines between a typical FGD generating in-depth engagement of a small closed group and a workshop engaging stakeholders without a pretense of non-hierarchical interaction within the group.(29)

CONCLUSIONS

The sub-national 5.3 policies in India are perceived to have reduced visible tobacco industry interference and in some instances partnerships between government agencies and tobacco industry. Despite the promise of these policies in reducing tobacco industry interference, their implementation remains far from effective. Major barriers to implementation include limited dissemination of, and awareness about, these policies, suboptimal functioning of the committees constituted to oversee policy implementation, low priority accorded to these policies (by non-health departments) and

interference by tobacco industry. A few states demonstrated practices that facilitated 5.3 policy implementation including display of policy signages and declarations, issuing of 5.3 policy orders by non-health departments, institutional channels to receive complaints about policy violations, and setting norm to not receive sponsorships by private entities in government departments. There is need to address several systemic challenges and learn from a few positive practices from past to optimise implementation of these policies.

DATA & SOFTWARE AVAILABILITY

Underlying data

This work draws on data from a brief questionnaire-based survey and focus group discussions. The survey data are all available in the Table 1 and Table 2. The FGD transcripts have not been made available in public domain due to ethical concerns. FGD participants included nodal officers for tobacco control from health and other non-health departments from Indian states. Typically, each state had a very few staff manning the state tobacco control cell/committee with a single nodal officer for tobacco control at state. Also, typically, non-health departments identify officers from their side who engage with state tobacco control cell/committee. Hence, even mention of a designation or even name of the state carry the risk of identifying the respondents. Tobacco control has been a very sensitive area riddled with conflicting interest within governments, commercial interests and often tobacco control officers/advocates have faced repressive actions. Hence, we have not put the FGD transcripts in public domain. We can make anonymised summaries from FGDs available to Editors/Reviewers if that helps in reviewing this paper. Others can write to the Ethics Committee at the Institute of Public Health Bengaluru (membersecretary@iphindia.org) requesting such access with justification.

Extended data

The survey questionnaire and the FGD guide are available here.

Software availability

LiberOffice is a free software that provides similar functions to the propriety software (MS Excel) used in this study.

COMPETING INTERESTS

Authors declare no competing interests.

FUNDING

This work was not funded through any specific grants. UB was supported for his time through the DBT/Wellcome Trust India Alliance fellowship [IA/CPHS/22/1/506533], and through an independent contract from the Vital Strategies (for data analysis and drafting of the report).

ACKNOLWEDGEMENTS

We greatly appreciate the support of the civil society partner teams, researchers, academic institutions, for their steadfast commitment and pivotal role in supporting the assessment exercise in the various states and union territories included in this work: Balajee Sewa Sansthan, Uttarakhand, E-Resource Centre for Tobacco Control (E-RCTC), Department of Community Medicine & School of Public Health, Postgraduate Institute of Medical Education and Research (PGIMER), Chandigarh; Generation Saviour Association (GSA), Punjab; Kerala Voluntary Health Services, Kerala; Mary Anne Charity Trust, Tamil Nadu; Madhya Pradesh Voluntary Health Association, Madhya Pradesh; Marathwada Gramin Vikas Sansthan, Maharashtra; Shikshit Rojgar Kendra Prabandhak Samiti (SRKPS), Jhunjhunu, Rajasthan; Socio Economic and Educational Development Society (SEEDS), Bihar and Jharkhand; and Uttar Pradesh Voluntary Health Association, Uttar Pradesh

We also thank officers from all the State Tobacco Control Cells, Department of Health, other government departments, and academia from all the states, who participated in this work. In addition,

we acknowledge the contributions provided by the staff, primarily Mr. Pranay Lal for guidance and support during the study proposal, Dr Garima Bhatt for supporting in the FGDs, Mr. Shammi Kumar, Mr. Keshav Garg, and Ms. Asha Tandon for logistics, financial and administrative support and coordination and Mr. Shravan Kumar for IT support.

REFERENCES

1. Lee Y, Kim S, Kim MK, Kawachi I, Oh J. Association between Tobacco Industry Interference Index (TIII) and MPOWER measures and adult daily smoking prevalence rate in 30 countries. Global Health. 2024;20(6) Available from: https://doi.org/10.1186/s12992-023-01003-x (cited 2025 Aug 20)
2. World Health Organization. WHO Framework Convention on Tobacco Control. Geneva: World Health Organization; 2003. Available from: http://www.ncbi.nlm.nih.gov/pubmed/20595357 (cited 2025 Aug 20)
3. Global Center for Good Governance in Tobacco Control. Handbook on the implementation of WHO FCTC Article 5.3: policies and practices that protect against tobacco industry interference. (third ed). Global Center for Good Governance in Tobacco Control; 2021. Available from: https://files.ggtc.world/uploads/2021-12-06/14-31-06-829409/Article%205.3%20Handbook%20GGTC%20Nov%202021.pdf (cited 2025 Aug 20)
4. Fong GT, Chung-Hall J, Craig L. Impact assessment of the WHO FCTC over its first decade: Methodology of the expert group. Tob Control. 2019;28:S84–8. Available from: https://tobaccocontrol.bmj.com/content/28/Suppl_2/s84 (cited 2025 Aug 20)
5. World Health Organization. WHO Framework Convention on Tobacco Control: guidelines for implementation Article 5.3; Article 8; Article 11; Article 13. Geneva: World Health Organization; 2009. Available from: http://whqlibdoc.who.int/publications/2009/9789241598224_eng.pdf (cited 2025 Aug 20)
6. International Union Against Tuberculosis and Lung Diseases. FCTC article 5.3 toolkit: guidance for governments on preventing tobacco industry intereference. Edinburgh: International Union Against Tuberculosis and Lung Diseases; 2012. Available from: http://www.seatca.org/dmdocuments/SEATCA-TII Index Report.pdf (cited 2025 Aug 20)
7. Assunta M. Global Tobacco Industry Interference Index 2023. Available from: www.globaltobaccoindex.org (cited 2025 Aug 20)
8. Goel S, Kar SS, Verma M, Sivanantham P, Naik BN, Gupta D. Evidence on article 5.3 of FCTC (tobacco industry interference in tobacco control activities) in India - a qualitative scoping study. BMC Public Health. 2021;21:1855. Available from: https://bmcpublichealth.biomedcentral.com/articles/10.1186/s12889-021-11773-x (cited 2025 Aug 20)
9. Kumar A, Bhojani U. Mapping tobacco industry interference in Indian states over the 3 decades: need for a nation-wide policy framework. In: 6th National Conference on Tobacco OR Health. New Delhi, India; 2024.
10. Rao N V, Bhojani U, Shekar P, Daddi S. Conflicts of interest in tobacco control in India: an exploratory study. Tob Control. 2016;25(6):715–8. Available from: https://tobaccocontrol.bmj.com/content/25/6/715.full (cited 2025 Aug 20)
11. Sharma R, Lal P, Pandey A, Tripathi G. Stopping Tobacco Industry Interference (TII): bottom-up approach to achieve national policy on FCTC Article 5.3 in India. Tob Induc Dis. 2018;16(1):20–2. Available from: https://www.tobaccoinduceddiseases.org/Stopping-Tobacco-Industry-Interference-TII-bottom-up-approach-to-achieve-national,84378,0,2.html (cited 2025 Aug 20)
12. Kapoor S, Yadav A, Lal P, Sharma R, RJ S, Pandey A. A “bottom-up approach” in devising national and sub-national policy framework in compliance with WHO FCTC Article 5.3 in India (EP-39-485). Int J Tuberc Lung Dis. 25(10):S212.
13. Bhojani U, Goel S, Kar S, Lal P, Chugh A. Tobacco industry interference. In: Swasticharan L, Arora M, Sinha P, Ray C, Munish V, Shrivastav R, editors. Report on Tobacco Control in India 2022 (vol 2). New Delhi: Ministry of Health and Family Welfare, Government of India;

2022. Available from: https://ntcp.mohfw.gov.in/assets/document/surveys-reports-publications/Report%20on%20Tobacco%20Control%20in%20India%202022_22%20April%202024.pdf (cited 2025 Aug 20)
14. Goel S, Rajkhowa P, Kapoor S, Pandey AK, Singh, RJ. Countering tobacco industry interference: a policy brief on strengthening the WHO FCTC Article 5.3 adoption in India. Indian J Com Med 49(2):S228-S233 Available from: https://journals.lww.com/ijcm/fulltext/2024/49002/countering_tobacco_industry_interference__a_policy.19.aspx (cited 2025 Aug 5)
15. Bassi S, Chopra M, Chugh A, Das S, Bhojani U, Nazar GP, et al. Trends in level of implementation of the WHO FCTC Article 5.3 in India. Tob Use Insights 2024;17 Available from: https://journals.sagepub.com/doi/full/10.1177/1179173X241271543 (cited 2025 Aug 20)
16. Chugh A, Bassi S, Nazar GP, Bhojani U, Alexander C, Lal P, et al. Tobacco Industry Interference Index: Implementation of the World Health Organization's Framework Convention on Tobacco Control Article 5.3 in India. Asia-Pacific J Public Heal. 2020;32(4):172–8.
17. Ralston R, Hirpa S, Bassi S, Male D, Kumar P, Barry RA, et al. Norms, rules and policy tools: understanding Article 5.3 as an instrument of tobacco control governance. Tob Control. 2022;31(1):S53–60. Available from: https://tobaccocontrol.bmj.com/content/31/Suppl_1/s53 (cited 2025 Aug 20)
18. Bassi S, Ralston R, Arora M, Chugh A, Nazar GP, Collin J. Understanding the dynamics of notification and implementation of Article 5.3 across India's states and union territories. Tob Control. 2022;31:s18–25. Available from: https://tobaccocontrol.bmj.com/content/31/Suppl_1/s18 (cited 2025 Aug 20)
19. Kumar P, Kamath V, Kamath A, Bhojani U, Chugh A, Bassi S, et al. Implementation of Article 5.3 of the World Health Organization Framework Convention on Tobacco Control: A Subnational Assessment. Indian J Community Med. 2022;47:531–5. Available from: https://journals.lww.com/ijcm/fulltext/2022/47040/implementation_of_article_5_3_of_the_world_health.14.aspx (cited 2025 Aug 20)
20. Bassi S, Mahajan R, Gupta PC, Arora M. Bridging the gap in the implementation of the World Health Organization's Framework Convention on Tobacco Control Article 5.3 in India: Insights from an opinion poll and stakeholder consultation. Int J Noncommunicable Dis. 2024;9(1):21–6. Available from: https://journals.lww.com/ijnc/fulltext/2024/09010/bridging_the_gap_in_the_implementation_of_the.4.aspx (cited 2025 Aug 20)
21. The Union. Tobacco control in India: the tobacco epidemic. 2023. Available from: https://theunion.org/our-work/tobacco-control/bloomberg-initiative-to-reduce-tobacco-use-grants-program/tobacco-control-in-india#:~:text=The Union works across 25,implementation of tobacco control policies. (cited 2025 Aug 20)
22. Lawshe CH. A quantitative approach to content validity. Pers Psychol. 1975;28:563–75.
23. Gilbert GE, Prion S. Making Sense of Methods and Measurement: Lawshe's Content Validity Index. Clin Simul Nurs 2016;12(12):530–1. Available from: http://dx.doi.org/10.1016/j.ecns.2016.08.002 (cited 2025 Aug 20)
24. Goel S, Rajkhova P, Kapoor S, Pandey AK, Singh RJ. Countering tobacco industry interference: a policy brief on strengthening the WHO FCTC Article 5.3 adoption in India. Indian J Community Med. 2024;49:S228-33. Available from: https://journals.lww.com/ijcm/fulltext/2024/49002/countering_tobacco_industry_interference__a_policy.19.aspx (cited 2025 Aug 20)
25. Sharma NC. 12 States Ban E-Cigarettes, Health Ministry Urges All To Follow. Mint;2019 Available from: https://www.livemint.com/news/india/12-states-ban-e-cigarettes-health-ministry-urges-all-to-follow-1552447122172.html (cited 2025 Aug 20)
26. ICMR National Institute of Cancer Prevention and Research & The Union. Orders & Notifications: Notifications on prohibition of gutkha and panmasala with nicotine in states of India. Available from: http://smokelesstobaccocontrolindia.com/orders-notifications/#! (cited 2025 Aug 20)
27. Kapoor S, Mehra R, Yadav A, Lal P, Singh RJ. Banning Loose Cigarettes and Other Tobacco

Products in India: A Policy Analysis. Asian Pacific J Cancer Prev. 2021;22(Supplement 2):51–7. Available from: https://journal.waocp.org/article_89829_5c1d86c9916c2f75f1c1c131edfc1ddc.pdf (cited 2025 Aug 20)

28. Global Center for Good Governance in Tobacco Control. Global Tobacco Industry Interference Index 2023: How deadly is tobacco industry influence in India? 2023. Available from: https://globaltobaccoindex.org/country/IN (cited 2025 Aug 20)

29. Caretta MA, Vacchelli E. Re-thinking the boundaries of the focus group: A reflexive analysis on the use and legitimacy of group methodologies in qualitative research. Sociol Res Online. 2015;20(4):1–13.

Figure 1

Click here to access/download;Figure;Fig 1_FGD participants.png

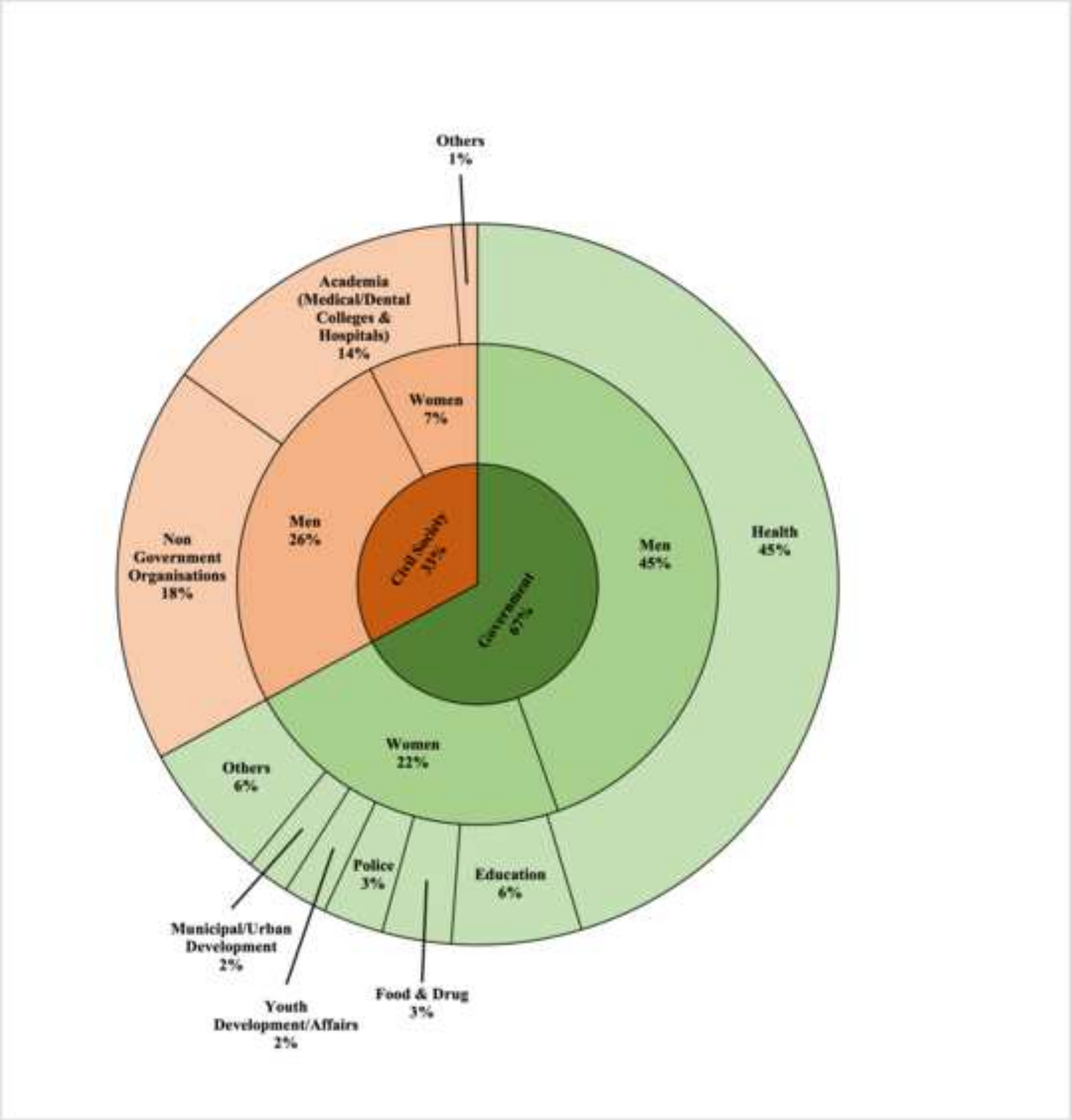

Figure 2

Click here to access/download;Figure;Fig 2_Vulnerability to TII.jpg

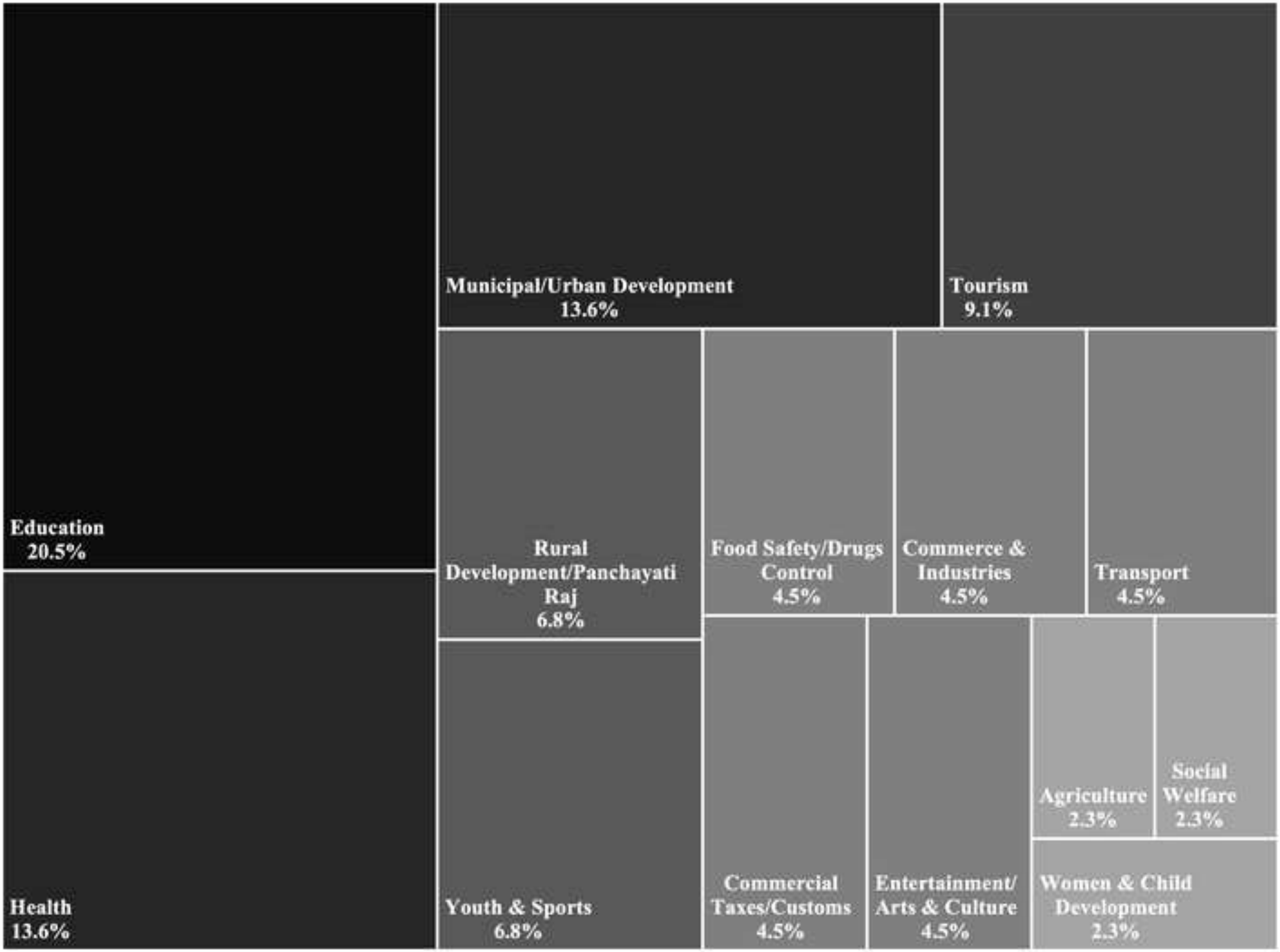

Supplementary File 1: Questionnaire

Click here to access/download
Supporting Information
Suppl File 1_Questionnaire.pdf

Supplementary File 1: Questionnaire

Click here to access/download
Supporting Information
Suppl File 2- FGD guide.pdf
