# Supplementary material for: Implementing the World Health Organization - Framework Convention on Tobacco Control Article 5.3: A qualitative study in 17 Indian states
Source: PLOS Glob Public Health. 2026 Jul 2;6(7):e0006522. doi: 10.1371/journal.pgph.0006522 (PMC13327259; doi:10.1371/journal.pgph.0006522)
Supplement: Attachment — Submitted filename: Response to Reviewers.docx [file pgph.0006522.s005.docx]

Dear Editor,

PLoS Global Public Health

Thank you for providing us with comments from the reviewers about our manuscript. We found these comments very useful in enhancing the manuscript. Please find in the appended Table, point-by-point response to all the comments by reviewers.

| **Reviewers’ comments** | **Authors’ responses** |
| --- | --- |
| It is a commendable study, well-structured and useful to get updated on the current status of the implementation of the Article 5.3 in Indian States. I compliment all co-authors for the their due diligence right from conceptualisation to this stage in publishing it. | Thank you! |
| The hesitancy or limitation of not mentioning the vital stakeholder- the Ministry of Health and Family Welfare who should be constantly promoting and supporting the State-based activities with a clear directive for its undertaking at the highest level- the State Cabinet is understood but I think that is where the research outcomes should mention it in a way that is acceptable to all. | We understand the reviewer’s push for mentioning names of the Indian states as well as the Ministry of Health and Family Welfare (national level) to enhance the advocacy value of the study findings.  We have mentioned states’ names in Table 1 and Table 2 but avoided them in the FGD excerpts/findings, as that carries a substantial risk of identifying respondents/participants of the study, who then face risks for the views they expressed under the condition of anonymity. We explain this constraint at the outset in the results section.  However, in line with the reviewer’s suggestion, we have now brought in the Ministry of Health and Family Welfare (national level) in the discussion, demanding their leadership in encouraging states (in adopting and implementing the 5.3 policies) as well as catalysing a nationwide 5.3 policy for India.  “Furthermore, having a national policy in line with the WHO FCTC Article 5.3 and integrating it into processes of the National Tobacco Control Program is likely to enhance implementation and monitoring/review of 5.3 policy implementation. In fact, the Ministry of Health and Family Welfare (Government of India) adopted a code of conduct to prevent tobacco industry interference in the year 2021, but its scope has been limited to the health institutions that are directly under the control of the ministry.(31) A nation-wide opinion poll of tobacco control stakeholders conducted in December 2021 revealed an overwhelming support (65% “strongly agreed”, 22.6% “agreed”) for a nation-wide 5.3 policy in India.(32) There have been repeated appeals for adopting such a policy in India.(33) Hence, the Ministry of Health and Family Welfare (Government of India) shall catalyze the development of a nationwide 5.3 policy for India as well as encourage states to adopt/implement 5.3 policies.”  Furthermore, within the results section, we have now mentioned names of the specific states while describing ‘good practices’, but not attributing excerpts from FGDs to specific state participant/s, as this part is less likely to impose adverse risks on participants in case of their potential identification. We hope this is acceptable to the reviewer. |
| The research is on an Important, policy relevant topic (state level implementation of WHO FCTC Article 5.3) and I thoroughly enjoyed reading it. The manuscript has real world utility and a solid mixed-methods backbone | Thank you! |
| The manuscript cites the same group of states as 64.3% of India’s population but uses different absolute numbers in different sections (e.g., ~999M vs ~909M). Please recompute using a single 2025 reference population and keep one absolute figure throughout.  Likewise, ensure a single anchor date for the policy counts (e.g., “as of July 2024” vs “by 2025”) and harmonize the narrative. | Thanks for flagging this mismatch. We have now updated these statistics based on India’s [projected population by the year 2025,](https://uidai.gov.in/images/AadhaarSaturationReport.pdf) wherein the selected study states account for 72.5% (~ 102.4 million) of the total population. We made it consistent across the two places.  We also harmonised the number of states (23), adopting the 5.3 policy by the year 2025 as per the following citation.  Goel S, Rajkhowa P, Kapoor S, Pandey AK, Singh, RJ. Countering tobacco industry interference: a policy brief on strengthening the WHO FCTC Article 5.3 adoption in India. Indian J Com Med 49(2):S228-S233 |
| “TCD’s partner organization(s)” appears without a prior definition in the main text; spell out Tobacco Control Department (TCD), Vital Strategies at first mention and clarify facilitators’ roles to avoid perceived influence. | We have now expanded the ‘TCD’ acronym. While the text in Methods (subsection: sampling, data collection and analysis) clarifies their role, we have now further added that these facilitators were employees of the partner organisations (and not that of Vital Strategies). |
| Expand Methods to include: Coder reliability: who coded; proportion double-coded; how disagreements were resolved; any intercoder agreement metrics or consensus procedures. | Unfortunately, we could not use independent coding of data by more than one researcher, mainly due to resource constraints. We have now clarified this in the revised manuscript.  “The data was coded based on pre-determined inquiry probes (i.e., awareness, challenges in implementation, implementation mechanisms, and suggestions for improvements) by UB, who has training in qualitative data analysis and has published qualitative studies. We could not use independent coding by more than one researcher, mainly due to resource constraints.” |
| Data completeness: only a subset of FGDs were audio-recorded; specify how non-recorded sessions were documented and how you minimized loss of nuance. | We have now clarified this further in the revised manuscript.  “The FGDs lasted for an average of 90 minutes (40-130 minutes), and 10 of the 17 were audio recorded. For the rest, we took detailed notes as we either did not get the consent for audio-recording (n=2) or the venue was too large, rendering the recording ineffective (n=5). These hand-written notes were made by assistants to the facilitators from the partner organizations and were later reviewed/edited by the facilitator for any missing/misinterpreted inputs.” |
| Add a note on how you manged hierarchical effects in mixed-seniority groups. The respondent mix appears weighted toward NGO/partner stakeholders, with relatively few direct government decision-makers. Add a paragraph on selection rationale, and potential biases. | We have included a paragraph in the Methods section on how facilitators went about purposive selection of FGD participants from tobacco control cells, other health department divisions, the relevant non-health departments, and civil society organizations. While, on the whole, government representatives (n=173) outnumbered civil society representatives (n=85), we agree that it would have been better to have greater participation from a variety of government agencies in the FGDs. Also, it wasn’t easy, and often impossible, to completely negate the hierarchical effect in the mixed-seniority groups.  We have now included a paragraph in the limitation section:  “It was not easy to convene the FGDs, especially getting adequate government representatives from non-health departments, given their hectic work schedules and competing priorities...The hierarchical pattern of reporting and relationships within and across government departments impacted the free and candid expression by participants, especially when senior bureaucrats (secretaries, commissioners, directors) participated, creating social desirability bias.(36) The same could have been the case for a few of the civil society representatives, whose work demanded close working with and/or funding from state governments. Hence, there was a blurring of lines between a typical FGD generating in-depth engagement of a small closed group and a workshop engaging stakeholders without a pretense of non-hierarchical interaction within the group.(37)” |
| Spellings to fix: “WHO Framework Convection on Tobacco Control” → Convention. “industry interreference” → interference (appears in Results/Impact section). “ACKNOLWEDGEMENTS” → ACKNOWLEDGEMENTS (section header). “LiberOffice” → LibreOffice; “propriety software” → proprietary. Table 1 column header “Whether such a body overswing …” → overseeing; | Many thanks for spotting these typos. We have now corrected these in the revised text. We have done careful copyediting of the revised manuscript. |
| This is a very interesting paper on the implementation on Art. 5.3 policies at the subnational level in India. I’ve enjoyed reading it and it will be a nice addition to the literature, based on a great dataset. | Thank you! |
| Abstract: You state in the methods: “The inquiry focused on policy awareness/dissemination, barriers and facilitators in policy implementation, the perceived policy impact, and respondents' suggestions for improvements.” But in the findings section you only report on barriers and facilitators. The conclusions remain unspecific (“several systemic challenges”… “a few positive practices from past”) – consider expanding on this (to save words, you could tighten the methods and phrasing elsewhere (e.g., “barriers and facilitators to implement these policies” ◊ “barriers and facilitators to policy implementation) | Thanks for this suggestion. We realised that the abstract in its earlier form was already exceeding the prescribed word limit (300 words). We have now revised it, making it shorter but also expanding on the conclusions section, making it more specific:  “The sub-national Article 5.3 policies have a promise to reduce industry interference. However, implementation of these policies ought to be strengthened by developing clear implementation guidance, mobilising overseeing committees, raising awareness among non-health departments, institutionalising complaint mechanisms, as well as periodic monitoring of industry interference in states.” |
| Introduction (Paragraphs 1 and 2): When you speak about “implementation gap” and “implementation” more generally, it remains unclear if you refer to the implementation of the FCTC (ie., adoption of national/subnational policies) or the implementation of the national/subnational policies. It would be very important in my eyes to make this explicit. | Thanks for your suggestion. We mean implementation of the WHO-FCTC Article 5.3. We have now clarified this by paraphrasing the respective sentences in the introduction section. |
| Introduction: The introduction currently does not cover the so what – i.e., so why does it matter? Consider adding a short paragraph at the end. | Thanks for the suggestion. We have now added sentences in this regard in the Introduction section:  “We believe that learnings from such a study will help enhance the implementation of 5.3 policies in these Indian states, safeguarding the prevailing effective tobacco control policy measures and allowing for the adoption and enforcement of stricter tobacco control measures in the future. Such lessons will also encourage and inform other Indian states that are considering adopting 5.3 policies, as well as other countries in conceiving and enforcing sub-national measures to prevent tobacco industry interference.” |
| Results/ rest: Based on methods/result, you only focused on barriers and not also on facilitators and you also cover challenges to policy adoption and implementation (and not just implementation). This seems inconsistent with abstract and intro – please ensure consistency. Please note that “good practice” are not the same as “facilitators”. | Thanks for pointing out this inconsistency in the framing. We have now revised the text in the concerned sections (abstract, introduction, results), stating that the study aimed at describing the state of 5.3 policies, the perceived challenges and good practices in implementation of these policies in states, as well as their perceived impact. This framing best suits the data we generated.  While the study was focused on the implementation of 5.3 policies, the FGD respondents occasionally dwelt on policy adoption challenges, and hence, we included that short section. We have now removed that from the results to avoid confusion – we now keep our focus on implementation and the perceived impact of 5.3 policies by stakeholders. |
| Results: It is important to make sure that it’s clear throughout the findings that you report what participants tell you and not as if these were facts. (see specific comments below) | We have revised the text, keeping this point in mind, and have addressed the subsequent comments by the reviewer in this regard. |
| Results: Words like “a few” or “many” states are not very precise – you might want to think about where you can report on numbers | As these findings are from the FGDs, wherein our data (transcripts) is largely qualitative in nature. Also, within a given FGD, at times, there were counter views on certain practices by the respective state. Hence, we preferred to focus on the thematic issues raised, but found it complex or difficult at best to arrive at specific numbers. |
| Discussion: the discussion is slightly repetitive and literature it bring is very limited and narrow. I recommend the authors look, for example, at the tobacco control policy implementation literature more broadly (for example, the barriers are pretty similar to what has been found elsewhere) | Thanks for pointing this out. We have now revised the discussion section, drawing on broader literature from India and globally situating our observations with respect to what we know from other studies. |
| Conclusion: I’m not sure this is here so much about repeating what you have done and found – it’d be good to strengthen the call for action and make it more specific. | Thanks for this suggestion. We agree. We have now revised the conclusion section (also the conclusion section in the abstract), making it more like a call for specific actions:  “Our study demonstrates the promise of sub-national 5.3 policies in visibly reducing tobacco industry interference in Indian states. However, there remain major challenges in the implementation of these policies. Building on the perceived/recognized challenges and demonstrated good practices in implementing 5.3 policies so far, our study points to the way forward in optimising these policies for safeguarding effective tobacco control measures in Indian states. Developing clear implementation guidance, mobilising the empowered committees into functional planning and overseeing bodies, raising awareness about tobacco industry interference and the 5.3 policies among tobacco control stakeholders (especially, non-health agencies), and institutionalising complaint/redressal mechanisms for 5.3 policy violations as well as periodic active monitoring of tobacco industry interference incidents in states are crucial measures to strengthen implementation of 5.3 policies in Indian states. Correcting some of the design gaps in the existing state-level 5.3 policies and adopting a nation-wide 5.3 policy would be crucial in preventing and/or reducing tobacco industry interference in tobacco-related public policies in India.” |
| Presentation: There are some issues with the writing. For example, articles are at times omitted or used where not needed, abbreviations are not always used consistently (e.g., CSR) and words are used repeatedly (e.g., 1st paragraph of the discussion uses “identified” 3 times). The writing can also be tightened and simplified without losing content in different places (e.g., “Respondents from one of the states” ◊ “Participants from one state”, “Despite a decade since the first such sub-national policy got adopted in India” ◊”A decade after the adoption of the first subnational policy on Art. 5.3 in India,…” I recommend the authors to have a close read through their paper | Thanks! We have taken a close look at the manuscript and addressed issues highlighted by the reviewer, including correcting grammar/syntax and bringing clarity of meaning. |
| - 25 and 112 – Consider starting with “The tobacco industry…” | Done! |
| - 35 – In the methods section of the abstract it sounds as if the survey was only used to inform the second stage and that the data wasn’t included in the final analysis. If this is not the case, please clarify. | This is not the case. We present the data from the survey in the Results section (Table 1) describing the features of the 5.3 policies in Indian states. We have now clarified this in the Methods section. |
| - 36 – Consider stating “adopted Article 5.3 policies” – please also correct where else this appears. | We corrected this throughout the abstract. In the main body of the manuscript, we have clarified the abbreviation in the introduction section at its first use. |
| - 36 – “that consented” – this sounds a bit odd as states cannot really give consent | This phrase is removed from the revised abstract. |
| - 37 – “self-administered survey” – “maybe an “online survey”? | Done! |
| - 61 – “from the past” instead of “from past”? | This phrase is removed from the revised abstract. |
| - 112 – What tactics? You could mention some examples – the journal audience is not just TC specialists. It would also be good to back this up with references | Thanks. We have now added a sentence explaining tobacco industry tactics and making an appropriate reference. |
| - 116 – not clear what the “such” refers to as it’s not described in the previous sentence. | Removed that word (“such”) and reframed the sentence. |
| - 117 – “member governments” – maybe better to just put “members” as it’s not just the governments that are members and as also the EU is a member. | Done! |
| - 118 – are there any later figures? 2020 seems quite a while ago | We updated this data for the year 2025.  “By the year 2025, about 102 countries reported implementing at least one measure aligned with the WHO-FCTC Article 5.3 implementation guidelines.(4)” |
| - 119 – you might want to explain what the implementation guidelines are and reference them (it would also be good to reference the WHO FCTC) | We have now added a line about the implementation guidelines and referenced both the WHO-FCTC and the WHO-FCTC Article 5.3 implementation guidelines. |
| - 122 – “huge implementation gap” – this either needs referencing or you need to end the sentence with “:”. | We added ‘:” at the end of the sentence, hence joining it with the next sentence that provides evidence of such an implementation gap. |
| - 124-5 – “the Article 5.3 had remained one of the least implemented provisions of the treaty.” – consider adding this to the previous paragraph and checking if you need the info before for the flow of your introduction | We prefer to keep this sentence in the given paragraph, as the focus of this paragraph is to highlight that implementation remains suboptimal globally and in India. |
| - 128 – considering adding the latest Index | We updated the data based on the latest 2025 Index. We also updated and clarified the comparison between the year 2023 and the year 2025 index reports.  “The Global Tobacco Industry Interference Index, which assesses the implementation of the WHO-FCTC Article 5.3, in its latest report in the year 2025, revealed that none of the 100 countries surveyed were immune to tobacco industry interference.(9) In fact, among the 90 countries that reported for the year 2023 as well as the year 2025, more than half of these countries (n=46) showed deterioration in implementation of the WHO-FCTC Article 5.3, while some (n=34) showed improvement over time.(9)” |
| - 129-31 – it would be good to clarify what the comparison here (compared to the 2021 report); I also think there were 90 countries in the report and I reckon you say 80 because 80 were in 2021 one. |  |
| 133 – this could be stronger as you haven’t clearly state the gap – is it that the there is little knowledge into Art. 5.3 policy implementation? | We edited the sentence to bring clarity:  “Given this suboptimal and worsening implementation of the WHO-FCTC Article 5.3, we turn the research gaze on the better understanding of implementation processes for the WHO-FCTC Article 5.3.” |
| - 135-7 – it would be good to back these two sentences up with references. | We have now added appropriate references to both these sentences. |
| - 137-8 – you could make this a bit more specific – e.g., where does India sit on the GTIII? | We have now edited this part, making it more specific. We added a new sentence:  “Further, reports show that India experiences the high degree of tobacco industry interference. With the Tobacco Industry Interference Index score of 59 out of 100 (the higher the score, the greater the interference), India was ranked at 48^th^ place among the 100 countries assessed in the year 2025.(9)” |
| - 146 – “in the implementation” instead of “in implementation”? | Done! |
| - 146-50 – This sentence is very long and quite hard to follow – can you simplify or split it into two? | Done! |
| - 155 – “Article” instead of “article” | The revised text does not have this usage. |
| - 163-4 – “more from a positivist perspective – what does this mean? I’m not sure you want to go into positivist vs interpretivist here as it raises questions about the knowledge generated here if there are different epistemological frames used | We have now dropped this phrase from this sentence. |
| - 169 – “implicit knowledge” sounds a bit odd – you could maybe just say “knowledge”? | Done! |
| - 169/70 – you might want to clarify here when you identified those. Earlier you said that today there are more so it could be good to be clear | Done!  “Based on the earlier documentation and our knowledge, we identified 19 states and union territories in India having 5.3 policies in some form by the year 2023.” |
| - 171 – can you say more about the administrative approval? From who did you need that and why? | We opted for approvals from the respective state departments of health and family welfare as the anchoring department for the WHO-FCTC Article 5.3 policies. It would have been difficult or impossible to engage government stakeholders in the FGDs without such permission. |
| - 174-5 – this needs a reference | We have now added a reference for this sentence. |
| - 175-8 – it would be good to mention this when you mention union territories for the first time – the reader may not be familiar with what they are. | Done! |
| - 181-4 – it’s good that you state this but it may not be ideal here since you have not yet described these tools. I recommend you mention this when you describe the survey and then refer to it again when it comes to the FGDs. | As this sentence about how we validated the tools applies to both the methods (survey, FGDs), we put it upfront to avoid repetition. If you insist, we shall change it as per your suggestion. |
| - 186 – why did you select the survey participants? This is quite important as you drew then on their information for the next part. And how do you know they filled it in close with the officials? Also, how many participants did you approach and how many completed it? | Thanks for pointing this out. We have now expanded on this, clarifying how we selected the respondents, their positionality as representatives of the civil society organizations working closely with the tobacco control cells of the respective states, and/or as project staff working at tobacco control cells of the state health departments. All 17 respondents consented and returned the completed questionnaires.  “We purposefully selected one adult respondent from each study state, who was actively working in tobacco control and had a close working relationship with the tobacco control cell (department of health) of the respective state governments. Respondents included a representative of the partner organizations of the Tobacco Control Division of the Vital Strategies from 12 states, one of the project staff working locally with state tobacco control cells in four of the states (i.e., Karnataka, Chhattisgarh, Jammu & Kashmir, Assam), and an official from the state tobacco control cell of Nagaland. All 17 respondents consented and returned the completed questionnaires… Respondents filled in the questionnaire in close consultation with the officials from the respective state tobacco control cell with whom they had a close working relationship.” |
| - 201 – What is TCD? | We expanded this now: Tobacco Control Department |
| - 201-2 – “facilitators” and “facilitating” in one sentence. I’m also not so clear what you mean here by “facilitating” as there are quite a few different actors – who organised it? Who invited participants? Who actually ran the focus group sessions? | We replaced “facilitated” with “conducted” the FGDs. In the subsequent sentences, we clarify the exact role (tasks) of facilitators. |
| - 204 – Here you state that the facilitators helped with the sampling, further up you and in the abstract you say that the surveys helped to identify relevant stakeholders – it would be good if you could clarify? Did the facilitators analyse the data from the survey, for example? | We edited this sentence, including changing the term “sampling” to “recruitment”. So, we purposefully sampled respondents based on the findings from the survey. However, the facilitators, who were tobacco control stakeholders from respective states, helped approach and recruit these potential participants for the FGDs.  “These facilitators, employees of the partner organizations, helped in recruiting potential participants. Participants were purposefully chosen based on findings from the survey and ensuring representation from the state tobacco control cells of health departments, other health department divisions (e.g., food safety, mental health, non-communicable diseases, oral health) as well as representatives from the non-health departments of state governments (e.g., education, municipal corporations, tourism etc.) and selected civil society entities including non-government organizations (NGOs) and academic institutions that were active in tobacco control in the state.” |
| - 209 – “Potential participants” instead of ”The participants” | Done! |
| - 210 – How many did you invite and how many did participate? | We invited a total of 275 participants and ended up having participation from 258 (participation rate of 93.8%). |
| - 217 – why did you ask about policy adoption given that your focus is on implementation? | We had a probe at the beginning of the FGD inquiry related to any challenges faced by participants in the adoption of these policies, as we assumed that any major issues during the adoption might have an impact on implementation (e.g., design gaps, industry resistance, conflicting interests within governments). However, the main focus (and hence, most questions/probes) was related to the implementation of the 5.3 policies. Now, based on another reviewer’s advice, we have removed the short section on “Challenges in Policy Adoption” from this paper, avoiding confusion for the readers. |
| - 218 – What do you mean by “implementation mechanisms”? | By implementation mechanisms, we meant the processes through which the implementation strategy operates, leading to the desired outcome (in this case, preventing tobacco industry interference). |
| - 218 – for FGDs, we usually say participants instead of respondents (also 238 and please check throughout the manuscript) | We have now ensured that the term ‘participant’ is used in place of ‘respondent’ in the context of the FGDs throughout the manuscript. |
| - 222 – who took notes? 1 person or more? | We have now clarified this in the revised text:  “These hand-written notes were made by assistants to the facilitators from the partner organizations or the state tobacco control cells and were later reviewed/edited by the facilitator for any missing/misinterpreted inputs.” |
| - 227 – “FGDs” instead of “FGD” | Done! |
| - 229 – can you describe what you mean by “thematic content analysis”? References could also help. | We used the term “thematic content analysis” as synonymous with “thematic analysis” and followed (now referenced in the manuscript) the six-step process (familiarization with data, generating initial codes, searching for themes, reviewing themes, defining and naming themes, and writing the report) described by Braun and Clarke.  Braun V, Clarke V. Thematic analysis. In: Cooper H, Camic PM, Long DL, Panter AT, Rindskopf D, Sher KJ, editors. APA handbook of research methods in psychology, Vol 2: Research designs: Quantitative, qualitative, neuropsychological, and biological. American Psychological Association; 2012. p. 57–71. |
| 243 – this is inconsistent with the methods where you say: “Respondents were  - 188 representatives from civil society organizations working in tobacco control in study states” | We have edited the text in the methods section, elaborating on sampling/selection of respondents and who these respondents were, removing the inconsistency noted by the reviewer. |
| - 248 – What was the average number of participants per FGDs? And what was the range? | On average, there were 15 participants per FGD, ranging from a minimum of 12 and a maximum of 20 participants. We have now added this to the Results section while describing participants’ characteristics. |
| - 248f - Your FGDs seem to have been quite large (and larger than standard FGDs) which raises some concerns, particularly if they only last 40 minutes given individuals very little time to speak, etc. It would be good to clarify why you thought the FGDs was the best approach (and not, for example, online survey) and why you decided to not split it up into more than one session? | We are combining these two comments as they refer to a similar concern. Indeed, as we reflected in the ‘limitation’ section, we often ended up with a large group of participants given the circumstances in government settings, resulting in inclusion of non-intended participants, as well as creating hierarchical voices within the groups.  As we admit in the limitation section, this is not what we intended, and this is not how FGDs are classically conducted. However, these group discussions were also not like workshops. Unlike classic workshops, these group discussions did not involve longer, in-depth, and hands-on engagement of all participants for creating solutions. In hindsight, we should have ideally conducted the FGDs separately for government and civil society representatives. Also, maybe individual interviews with government stakeholders (while time and resource-intensive) would have helped address hierarchical tension, possibly suppressing the expression of certain views in our FGDs. So, while we prefer to retain the term FGD (in the lack of another appropriate term), we have now revised the limitation section, bringing in these points. |
| - 705 – your reflection here made me wonder if calling what you have done a FGDs is actually the most appropriate word. Or was it maybe a workshop? Please reconsider your label. |  |
| - 248 – were the survey participants also part of the FGDs? It would be important to clarify. | Yes, in the revised manuscript, we described who these survey respondents were: they included representatives of the civil society partners of Vital Strategies, who also served as facilitators for the FGDs. |
| - 257 – Start a new paragraph with “Typical,…” | Done! |
| - 257-64 – Consider changing the structure of this (e.g., When presenting the results, we do not mention state names but have assigned each state a number. This is to avoid…) Please also consider if this is not rather about the methods” | Done! |
| - 278-9 – you could provide references here. | These data came from an analysis of the survey data, negating the need for external reference. |
| - 279-81 – three times “issued | We reframed the sentence, removing the repetition of this term. |
| Table 1 – “Was such” instead of “Whether such” for the last two questions | Done! |
| - 296-306 – I had thought that you wanted to keep this here descriptive, so consider if this doesn’t rather go under challenges? | Thanks! Yes, this would have been a better fit under the section “Challenges in Policy Adoption”. However, based on another reviewer’s suggestion, we have removed that short section to mainly focus on the implementation of 5.3 policies. As the issue highlighted here concerns policy adoption/design, we have retained it in this section that describes policies. |
| - 310-1 – For me, this sentence is discussion rather than your findings. | We have now removed this sub-section on “Challenges in Policy Adoption” from the revised manuscript. |
| - 311 – For how this section is written, it doesn’t become clear what these two are. Make it explicit in how you present the findings. |  |
| - 328 – “believed” might not be a good word as you ultimately don’t know what people believe, better use report, state or similar |  |
| - 345 – maybe clarify that this about awareness among participants? | Done!  “On the whole, awareness about 5.3 policies was limited among participants from government agencies.” |
| - 356-8 – “However, even in these states, the detailed knowledge about various provisions in these policies was typically limited to health department officers.” – you need to make clear that this what the participants said – e.g., participants reported/perceived… | Done!  “However, even in these states, the detailed knowledge about various provisions in these policies was reported mainly by participants from health departments.” |
| 370 – This seems to be closely tied to “Low awareness” as it is one explanation for it? So why are they separate? It’s also not so clear if dissemination is about dissemination within the public sector or to the public or all. | Yes! We kept it separate to spotlight this weakness within government channels. However, we have now added a couple of sentences at the start of this theme clarifying the reviewer’s concerns.  “This theme concerns the limited dissemination of 5.3 policies not only within government agencies but also to other tobacco control stakeholders and the public in general. This seems to be one of the reasons for limited awareness about 5.3 policies among stakeholders.’ |
| - 371-6 – Also here, you need to make clear that this is what participants said (same 387-92) | Done! |
| - 395 – I think you can be more specific here and say in our many states this was based on the survey data? | Done! (12 out of the 17 states) |
| - 397-8 – “Such was likely a case for many more states” – maybe rather “Participants suspected this could be the case…” or is this you suspicion? If it’s yours, then it’s more a discussion point | It was based on inputs from participants. We have now edited it as per the reviewer’s suggestion. |
| - 408 – is this really just about “implementation guidance”? Something like having a clear definition of the tobacco industry is maybe not just about guiding implementation? Point 2 and 3 are then more about enforcement and 4 about oversight, etc. | The participants brought these issues as challenges in implementing the 5.3 policies. We recognise that concepts like ‘implementation’, ‘enforcement’ could have different interpretations for different stakeholders. We, as authors, gave primacy to participants’ conception of what they saw as implementation challenges. In a way, this conception is in line with one of the frameworks^*^ for tobacco control law implementation that sees implementation as an outcome of three processes: awareness (about the law), enforcement (of the law), and review (or the oversight).  ^*^Richardson, Y. Implementation framework. Presented at International Legal Consortium seminar series-Seminar on legal strategies for implementing tobacco control laws, 23rd June 2014, Washington DC |
| - 427 – the quote talks about “general lack of awareness” is this not your first theme? | We wanted to highlight here the other two issues that come up in this quote, i.e., low priority for this policy amidst other priorities of non-health departments, and frequent turnover of staff in non-health (esp. police department). We have now edited the quote, removing the portion about ‘lack of awareness’ to bring clarity and focus on the issues we wanted to highlight from that FGD. |
| - 446 – can you clarify what you mean by front group? | By ‘front group’, we mean an entity/organisation that purports to represent one agenda while, in reality, it serves some other party or interest whose sponsorship/support is hidden or rarely mentioned. We now clarify this in the manuscript and provide a citation. |
| 459 – this paragraph interrupts the narrative here as it’s not really about implementation challenges but about perceived vulnerability | This perceived vulnerability is based on participants’ experiences of tobacco industry interference with specific government departments. As they also spoke at length about such examples, we felt it would be important to present a summary of these data, as it informs prioritization in protecting specific government departments from tobacco industry interference. |
| - 482-3 – Please clarify that this is about participants’ reporting | Done! |
| - 485 – “from at least 3 states” – can you not specify how many it was? | Sorry! This was an incorrect framing. We edited this. Participants from three of the FGDs mentioned the display board. |
| - 486-7 – “mentioning the need for prior permission/procedure to be followed by the tobacco industry in meeting government officials” – it’s not clear what the permission is for. | The permission was to be sought by the tobacco industry from (specified) senior officer from the health department to meet any government representatives. We have edited this sentence for clarity.  “Participants from three of the 17 states (Kerala, Jharkhand, Uttar Pradesh) mentioned about display of such signage at government offices at the state and district-level, mentioning the need for the tobacco industry to seek prior permission or follow a specific procedure if it intended to meet with government officials.” |
| - 497 – “Though not mandated by 5.3 policies” – so this is not really about implementation of the policy, so is this really in the right section? | We kept this example as one of the ‘good practices’ as it is aligned with raising awareness about the tobacco industry interference. |
| - 526 - “three of the states reported” – states cannot report (also 564) | We edited the sentence, stating “Participants from three of the states reported…” |
| - 526 – “groups” instead of “group” | Done! |
| - 538 – I’m not sure it’s very clear what you mean by normative measures and norms. You could for example, already bring this up in the introduction and define it there. Also here, please make sure it’s clear that this was reported by participants. | By normative measures, we meant measures beyond mandated provisions in the 5,3 policies that states took to enhance awareness and implementation of 5.3 policies. We have clarified this in the first sentence of this sub-section. |
| - 564 – here you mention 7 states, there are 8 in the table (heading “brief account of complaints” – and for Assam and Meghalaya it says: “There was probably no formal complaint” – make sure you are consistent and use the correct wording | Thanks for pointing out these inconsistencies. We edited the text now mentioning eight (and not seven) states. We framed the text/Table about violations (through external complaints or Suo motu cognizance by states) and resultant actions.  “Eight of the 19 states studied reported having received complaints about or taken Suo motu (on its own accord) cognizance of the violation of the 5.3 policy.” |
| - 591 – Here it’s CSR, elsewhere its corporate social responsibility – it’d be good to introduce the abbreviation the first time and then use it consistently. | Done! |
| - 627 – “implementation remains very limited” – can you explain what you mean by this? | We now reframed this sentence for clarity:  “Overall, our findings imply that despite some years (2-10 years) of having 5.3 policies in place, there remain several challenges in their optimal implementation.” |
| - 651 – What do you mean by “time-bound resolution procedures”? I don’t think this came up before. And why are complaints hindering the implementation of the policy? Is this about lack of definition as well? Then this needs to be clearer. | What we meant here as a gap, is the lack of clear guidance in 5.3 policies about how people can complaint about policy violations (whom and how to complaint) as well as lack of guidance on how the concerned committee/officers need to deal with any reported/recognised violations (through penal/non-penal provisions, time limit within which such violations are to be dealt with). We have now edited the sentence, bringing clarity of meaning.  “While these gaps represent weaknesses in the design of these policies, our study highlights how some of these gaps – not issuing the policy through a higher office/state gazette, not clearly defining the tobacco industry, not defining the role of the empowered committee, not defining the process of how complaints/violations can be reported and resolved – hinder the implementation of the prevailing 5.3 policies. |
| - 705 – You could connect the limitation you identified to the literature (e.g., social desirability bias). | We have done so now, including a reference to relevant literature.” |
| - 705 – While I understand that organising the sessions was challenging, I wonder if you did anything to navigate the power dynamics (e.g., did you invite people to contact you afterwards if they wanted to share anything else?) | We could not really address the power dynamics in this study. We, however, had encouraged participants to reach out to us for any further inputs. The participant information sheet contained the contact information to reach the PI. We have now added more lines in the limitation section reflecting what we could have done (in hindsight) to address power dynamics.  “In hindsight, we feel it would have been better to conduct separate FGDs with government and civil society representatives. Also, individual interviews with government representatives (though time and resource-intensive) instead of FGDs would have helped avoid the hierarchy of positions/views that we observed in FGDs.” |
| - Figure 1: The text is quite blurry and hard to read | We have now revised the figure, enhancing the readability of the text within it. |
