# Supplementary material for: Implementing the World Health Organization - Framework Convention on Tobacco Control Article 5.3: A qualitative study in 17 Indian states
Source: PLOS Glob Public Health. 2026 Jul 2;6(7):e0006522. doi: 10.1371/journal.pgph.0006522 (PMC13327259; doi:10.1371/journal.pgph.0006522)
Supplement: Attachment — Submitted filename: response to peer review comments_April 11.docx [file pgph.0006522.s006.docx]

Dear Editor,

PLoS Global Public Health

Thank you for providing us with further comments from the reviewer-3 about our revised manuscript. We found these comments very useful in enhancing the manuscript. We have now revised the manuscript by addressing all the comments. The appended Table provides point-by-point response to all the comments by the reviewer.

Yours sincerely,

Upendra Bhojani

| Sr. No. | Reviewer-3 comments | Authors’ responses |
| --- | --- | --- |
| 1 | Thanks for the opportunity to re-review this paper. I thank the authors for engaging carefully with the previous comments and making substantial changes to the manuscript. The paper has improved; however, I believe further work – primarily focusing on presentation and clarity – is still needed to maximise its potential. | Thanks!  We have now revised the manuscript further addressing all the specific comments by the reviewer. |
| 2 | I recommend tightening the manuscript overall, particularly the Results section, to enhance readability. For instance, the first paragraph of the Findings could be shortened (e.g., “we first describe participants’ characteristics before presenting…”). Similarly, the survey sample description could be streamlined (“17 individuals (1 from each state) filled the survey…”). Since familiarity with Article 5.3 is part of the inclusion criteria, some of this information may be redundant. There are also several instances where phrases and repetitions could be simplified (e.g., “some of the participants” → “some participants”; “impeding effective implementation of these policies, including limited dissemination and awareness about these policies” → “impeding effective policy implementation, including limited dissemination and awareness”). These edits would improve conciseness. | We have carefully edited the manuscript including the sections specifically highlighted the reviewer, making it concise and enhancing readability. |
| 3 | Results (use of quotes): At present, the qualitative quotes are not integrated effectively. In some paragraphs, multiple aspects are discussed first, and then quotes are added afterwards, which interrupts the flow and creates a sense of jumping back and forth. I recommend embedding quotes directly into the relevant discussion to support or illustrate specific points. This will also help avoid instances where the quote merely repeats what the text has already stated – the quotes should add value rather than simply confirm. | Thanks for this suggestion. We have now carefully edited the Results section. We rearranged the quotes, placing them at most appropriate places, enhancing the flow of the text. We also edited the text so as to avoid repeating the messages that are apparent from the FGD excerpts. |
| 4 | Discussion (engagement with literature): Please ensure stronger engagement with the wider literature, including work beyond India. The discussion currently draws heavily on the Tobacco Control Special Issue, while there is broader relevant literature that could strengthen the paper’s contribution.  For example, in the “design gaps” section, there is limited engagement with research on whether policy design commonly hinders effectiveness in tobacco control. Similarly, the “raising awareness” section begins like a literature review but then shifts quickly back to findings without integrating the two. The monitoring section also presents opportunities to incorporate wider scholarship on monitoring and implementation. | We have now carefully edited the Discussion section. We strengthened this section, including the specific sub-sections highlighted by the reviewer, by bringing in relevant literature from and outside of India. |
| 5 | Two consecutive sentences begin with “India” (“India experiences a high degree of tobacco industry interference. India saw several states…”). Consider revising for variation. | Done! See lines 24-28:  “India experiences a high degree of tobacco industry interference…Several states in India adopted such policies in the last decade.” |
| 6 | The sentence about industry interference interrupts the flow and may work better earlier in the section. | Done! We moved it at the beginning of the Introduction section, enhancing the flow of the text. See Line 24-27:  “India experiences a high degree of tobacco industry interference. The World Health Organization Framework Convention on Tobacco Control, in its Article 5.3, requires members to protect their policies from tobacco industry influence.” |
| 7 | “policy implementation the perceived policy impacts” appears to be missing a word. | Done! See lines 34-35:  “The inquiry focused on understanding the prevailing policies, perceived challenges, and good practices in policy implementation as well as the perceived policy impacts.” |
| 8 | It would be helpful to describe the sample at the beginning of the findings section in the abstract. | Done! See lines 39-40:  “There were 17 respondents for the survey, and a total of 258 participants in 17 focus group discussions across states. |
| 9 | Ensure that results are clearly framed as participant perceptions (e.g., “being the most vulnerable” → “perceived as the most vulnerable”). | Done! Lines 43-45:  “Major perceived challenges in the implementation of Article 5.3 policies included … Several government departments were perceived vulnerable to industry interference, with the departments of education, health, and municipal administration perceived as the most vulnerable.” |
| 10 | Paragraph 1: Please consider referencing the implementation guidelines. | Done! Added the reference to the WHO Article 5.3 Implementation Guidelines. |
| 11 | Paragraph 2: The first few lines (until “Article 5.3 had remained…”) interrupt the flow and may be removable. | Done! |
| 12 | Paragraph 2: The GTI should be explained more clearly, as not all readers will be familiar with it. | Done! See lines 84-89:  “The Global Tobacco Industry Interference Index is an annual global survey wherein civil society actors use publicly available information to capture the nature and extent of tobacco industry interference in participating countries as well as the measures taken by these countries to protect tobacco control policies from the industry influence.(9) The Index assigns a score (0-100) to each country wherein the bigger score implies greater degree of tobacco industry interference in that country.(9)” |
| 13 | Paragraph 3: It would be useful to briefly explain why findings from India are relevant to other contexts (transferability). | Done! See lines 103-108:  “Given the overall paucity of evidence around implementing the WHO FCTC Article 5.3 and given that several countries, especially in South Asia but also others, share one or more features of the Indian context (federal democracy, heterogenous tobacco industry, diversity of tobacco products beyond cigarettes, large population of Indian states often similar to, or more than individual countries), we believe that lessons from this study will have relevance beyond India.” |
| 14 | Paragraph 4: Consider dropping “Yet,” at the beginning. | Done! |
|  | There seems to be a discrepancy between the abstract (17/19 states) and the introduction (14 states with policies at the time of study conception). | We see that such confusion arise because we used the phrase “By the year 2022 – the time this study was under conception…”. It took a while before executing this study. By the time we did data collection in the year 2023, the number of states adopting the 5.3 policies went up from 14 to 17. To avoid such confusion, we have now removed the phrase “the time this study was under conception” from that sentence. |
| 15 | “self-administered survey” → clarify whether this was an online survey. | Done! See lines 153-154:  “We conducted a qualitative study in 17 Indian states that have adopted 5.3 policies in some form, using an initial online self-administered survey followed by focus group discussions (FGD) with relevant stakeholders from governments and civil society.” |
| 16 | “These states account…” → consider “Taken together, the 17 states included in the study account…” | Done! See lines 170-172:  “Taken together, the 17 states included in the study account for about 72.5% (about 102.4 million in the year 2025) of India’s population.” |
| 17 | “of the Vital Strategies” → “of Vital Strategies” | Done! |
| 18 | Consider starting a new paragraph with “The questionnaire had…” | Done! |
| 19 | “Enquiring about the 5.3 policy” → “enquiring about the Article 5.3 policy” (and consider using “Article” consistently throughout). | Done! We effected this change throughout the manuscript. |
| 20 | Could you clarify why FGDs were led by facilitators rather than the researchers? | As clarified in Methods section (lines 206-209), the PI or one of the CoIs (researchers) along with facilitators (many of whom were also researchers) jointly conducted the FGDs. Facilitators helped in recruitment of participants and were very useful in co-conducting the FGDs as they were the ‘known faces’ in those states having rapport with many of the tobacco control stakeholders in that state. |
| 21 | “Thematic content analysis”: Since you cite Braun and Clarke, do you mean reflexive thematic analysis? If not, you may need additional methodological references. Note that edits here may require updating the abstract. | Thanks for pointing this out! Yes, we meant reflexive thematic analysis – so, also clarifying researchers’ positionality elsewhere in the manuscript. Use of the generic ‘thematic analysis’ was unintentional. We have updated the term in Methods as well as Abstract section. |
| 22 | “had a very few” appears to be a typo. | Yes! Corrected now! See lines 282-284:  “This is to prevent the identification of participants because each state had a very few staff members manning the state tobacco control cell/committee with a single nodal officer for tobacco control. |
| 23 | Figure 1: Adding two variables makes the figure confusing, and gender is already reported in the text. The figure also appears blurry and difficult to read. | Given the disparity in gender-based participation in FGDs, we included that layer in the Figure-1. If it’s acceptable, we prefer to retain that. We are reproducing the Figure-1 in better resolution at the end of this document for ease of reading it. |
| 24 | About 5.3 Policies in Indian states”: Consider starting with “According to survey participants…” to make the data source explicit. | Done! |
| 25 | Some of the FGD participants opined…” → “Some FGD participants argued…” (tighten phrasing). | Done! |
| 26 | “authority/compliance”: These are quite distinct concepts and may need unpacking. I am also unsure whether the second quote adds much. | We now unpacked this and reorganised the quotes/text for clarity of reading. See lines 377-390:  Some FGD participants argued that ideally, a policy at the state level ought to be issued through a cabinet approval and published in the state gazette. Such policy will be seen with a greater legitimacy compared to an order issued by an executive.  *“It (Article 5.3 policy) isn’t a policy document… It is not issued through cabinet approval…it is an executive order at the state level.”* (FGD-13)  In absence of a cabinet approved gazette policy, an order issued by a senior bureaucrat within the health department (typically a secretary, or a commissioner) evokes better legitimacy compared to the one issued by lower-level officer.  *“We faced challenges in convincing superiors of the need to issue a policy…So, we issued it, but we realized that at the district level, district collectors are not giving any importance to the policy as it is not issued by the department head”* (FGD-10) |
| 27 | Awareness: Some quotes may fit better under “Limited dissemination.” | On a careful reading, we felt it is best to integrate both the themes as limited dissemination was the main reason for stakeholders’ low awareness about Article 5.3 policies. We have now done that. |
| 28 | Awareness: The last quote mentions Uttarakhand, although I understood that states would not be named. | Thanks for pointing this out. We have now removed this identifier from the quote. |
| 29 | Dissemination: The section opening is long-winded. Consider starting more directly (e.g., “Participants reported/suspected that Article 5.3 policies were sent…”). | We merged the two themes about low awareness and limited dissemination. We edited the text making it concise and enhancing the readability. |
| 30 | Empowered Committees: “from certain states specifically” is unclear wording - please revise. | Done! See lines 500-501:  “Participants from some study states highlighted that these committees, while constituted in these states, have not met since their constitution.” |
| 31 | Perceived impact: Under “impact,” I expected more explicit discussion of whether the policy reduced public-sector engagement with the tobacco industry. Complaints and actions seem more related to implementation/enforcement than impact. | We feel that both, due to our qualitative approach (esp. through FGDs) as well as due to lack of advanced level of enforcement of these Article 5.3 policies, we are not having more ‘concrete’ impacts to report. However, specific actions like severing existing ties with tobacco industry in some of the states imply some reduction in public-sector engagement with tobacco industry. |
| 32 | Bottom-up initiatives: Given the title, I expected more discussion of the “promise” of bottom-up initiatives, why they may be more prominent in India, and whether similar patterns exist elsewhere (e.g., the US). | We have now added text to strengthen the discussion on this aspect. See lines 962-978:  “This phenomenon is not isolated in Indian context as many of the tobacco control regulations in India were initiated at state level (a few even at district level) before they were enacted at national level (e.g., prohibition of electronic nicotine delivery systems(25)) or adopted by most or all of the sub-national jurisdictions across India (e.g., prohibition on certain forms of smokeless tobacco(26); prohibition on sale of single/loose cigarettes(27)). The Indian constitution divides legislative power across national and state governments.(28) While tobacco industry (especially the levy of excise) comes under the legislative power of national government, health (especially, “Public health and sanitation; hospitals and dispensaries”) comes under the legislative power of state governments.(28) It is this mandate of protecting and promoting health that has prompted state governments to champion tobacco control policies in India.  There are a very few other examples of sub-national level policies in line with the Article 5.3. For example, while the United States of America has not signed the WHO-FCTC, the California Tobacco Control Program endorses guiding principles of the WHO-FCTC Article 5.3.(29) It prohibits the grantee to have any conflicts of interests with commercial tobacco industry. In a global tobacco control context, where the adoption of policies in line with the WHO FCTC Article 5.3 remains limited, the Indian experience indicates the promise of bottom-up regulations in federal democracies.” |
| 33 | Under “bottom-up initiatives,” Batti is mentioned mainly for the number of states, which adds little given Table 1. | We prefer to retain this reference (Bassi et al) to highlight incrementality in the number of Indian states adopting Article 5.3 policies over time. I hope that is acceptable. |
|  | Limitations: While I appreciate the revisions, the section currently undermines the study quite strongly by stating what should have been done differently. Consider striking a better balance. | Thanks for your suggestion! We have now toned down this section striking a better balance. See lines 1092-1105:  “While the embeddedness of this study – wherein participants included government officers across departments with leadership and/or professionally mandated roles for tobacco control – is the core strength of this study, it affected the rigor of data collection process. It was difficult to get adequate participation from non-health departments, given their hectic work schedules and competing priorities. The hierarchical pattern of reporting and relationships within and across government departments impacted the free and candid expression by participants, especially when senior bureaucrats (secretaries, commissioners, directors) participated. This would have potentially created some degree of a social desirability bias.(38) The same could have been the case for a few of the civil society representatives, whose work demanded close working with and/or funding from state governments. Hence, there was a blurring of lines between a typical FGD generating in-depth engagement of a small closed group and a workshop engaging stakeholders without a pretense of non-hierarchical interaction within the group.(39) However, we believe that a relatively large participation across 17 FGDs in diverse state contexts would have ensured that we did not miss any major perceived challenges/facilitators in Article 5.3 policy implementation.” |
| 34 | Discussion/conclusion: It may be worth having a separate implications section, as including all implications in the conclusion makes it quite busy." | We converted ‘Conclusions’ into ‘Conclusions & the Way Forward’, and divided the text into two paragraphs to make it easier to read. I hope that is acceptable. |

**Fig 1 Characteristics of FGD participants**

[Fig 1 provides the distribution of FGD participants as per their gender and the sector (government, civil society) they worked in.]
