# Supplementary material for: Implementing the World Health Organization - Framework Convention on Tobacco Control Article 5.3: A qualitative study in 17 Indian states
Source: PLOS Glob Public Health. 2026 Jul 2;6(7):e0006522. doi: 10.1371/journal.pgph.0006522 (PMC13327259; doi:10.1371/journal.pgph.0006522)
Supplement: Attachment — Submitted filename: Response to Reviewer Comments_24 April 2026.docx [file pgph.0006522.s007.docx]

Dear Editor,

PLoS Global Public Health

Greetings from India!

Thank you very much for your timely revert providing editorial as well as review comments. We found these comments very useful. We have now revised the manuscript by addressing all the comments. The appended Table provides point-by-point response to all the review and editorial comments.

Yours sincerely,

Upendra Bhojani

| **Sr. No.** | **Reviewer-3 comments** | **Authors’ responses** |
| --- | --- | --- |
| 1 | I’m glad to see that the authors found the feedback helpful and they have done a great job responding to all comments. I only have a few minor comments to add. | Thanks!  We have now revised the manuscript further addressing all the specific comments by the reviewer. |
| 2 | Abstract: Introduction – “their policies/ “this policy” – is a bit confusing for readers. It’d be good to clarify in the second sentence what you mean by “this policy”. | We now edited this part for the clarity. See Lines 24-29:  “The World Health Organization Framework Convention on Tobacco Control, in its Article 5.3, requires members to protect their policies from tobacco industry influence. Despite the global guidance, the implementation of the Article 5.3 of the convention remains far from desired. Several states in India adopted policies in line with the Article 5.3 of the convention in the last decade. We study implementation processes concerning these policies.” |
| 3 | Abstract: Methods – “Thematic content analysis” is usual – was a thematic or content analysis? It also appears again in the methods where you also refer to Braun and Clarke, I reckon you did (reflexive) thematic analysis? | That’s correct! We did reflexive thematic analysis as described by the Braun and Clarke. We have now edited the text in Abstract as well as Main Text for clarity:  See lines 36-37:  “We used reflexive thematic analysis to understand policy implementation processes.”  See limes 217-218:  “We used reflexive thematic analysis following the six-step process described by Braun and Clarke(22)…” |
| 4 | Abstract: Results – consider using “remained” instead of “remain” for consistency. | We do not see use of the term “remained” in the Result section of the Abstract. We assume that the reviewer is referring to the Introduction section of the Main Text here. We edited that line for consistency:  See lines: 77-78:  “The WHO-FCTC assessment, after a decade of its existence, demonstrated that the Article 5.3 remain one of the least implemented provisions of the treaty.(6)” |
| 5 | Introduction: “about 102” – If it is a 102, you can remove “about”. | Done!  See lines: 74-75:  “By the year 2025, 102 countries reported implementing at least one measure aligned with the WHO-FCTC Article 5.3 implementation guidelines.(4,5)” |
| 6 | Introduction: “in countries that have already adopted” – do you mean “ratified”? | We carefully checked the Introduction section and the entire manuscript but could not locate this phrase. We ensured that in context of the WHO-FCTC, the term ‘ratification’ and not ‘adoption’ is used. |
| 7 | Methods: “are union territories at the time of the study” – “were union…”? | Done!  See lines 148-150:  “For this paper, we will use ‘state’ as a generic term to refer to each of these sub-national units, even if a few of these (Puducherry, Jammu & Kashmir) were union territories at the time of the study.” |
| 8 | Methods: “These states account…” – I suggest you say “These 17 states” as in the previous sentence you refer to three so the reader may wonder what “these states” refer to. | I think the reviewer have probably referred to an older version of the manuscript as the latest version submitted does not have this phrase – It was already corrected for clarity e.g., “Taken together, the 17 states included in the study account for about 72.5% (about 102.4 million in the year 2025) of India’s population.” |
| 9 | Methods: “of the Vital Strategies” – is it not just “of Vital Strategies”? It’s mentioned a few times. | Done! This is corrected in Methods and the Funding Statement. |
| 10 | Methods: “These partner organizations were trained/calibrated through…” The next sentence also starts with “These”. I suggest you revise the phrasing. | This part if now edited for clarity.  See lines 185-190:  “The principal investigator (SK) and a few of the co-investigators (PC, GB, AY), along with the facilitators (representatives of the partner organizations of the Tobacco Control Department of Vital Strategies) in each state, conducted the FGDs. These facilitators, employees of the partner organizations, helped in recruiting potential participants. Facilitators were trained through an online workshop about the purpose of conducting FGDs, the FGD guide, and the process.” |
| 11 | Results: “Fig 1 Characteristics of FGD participants” – consider moving this up a paragraph so that the figure is located after the paragraph where it is mentioned. | Done! |
| 12 | Results: “In the rest of the six states” – is not very clear, maybe “In the other six states”? | Done!  See lines 310-311:  “In the other six states, nearly all the participants representing various government departments were aware of the Article 5.3 policy in their state.” |
| 13 | Discussion: “for 5.3 of the WHO FCTC” – consider inserting “Article” | Done! Also corrected at the other few places in the Main Text. |
| 14 | Conclusion – You might want to mention the WHO FCTC in the first sentence. | Done!  See lines: 725-726:  “Our study demonstrates the promise of sub-national policies in line with the Article 5.3 of WHO-FCTC in visibly reducing tobacco industry interference in Indian states.” |
| 15 | I suggest the authors closely reread the manuscript and check for issues similar to those I picked up (e.g., connections, clear reference points) | Thanks! We have done a careful and close reading of the manuscript and made additional minor edits at a few places to bring clarity for readers. |
|  | **Editorial Comments** | **Authors’ response** |
| 15 | You have stated a qualitative study design in your title; however, the methodology section mentions the use of an online survey, which may confuse readers because surveys are typically associated with quantitative research. Please clarify this by explaining that the online survey was used solely to collect qualitative data, and that it included open‑ended questions based on the interview guide or checklist. This clarification will help readers understand that the survey functioned as an alternative mode for gathering narrative, qualitative responses rather than quantitative data. | Thanks for flagging this. We now edited the text in Methods section bringing this clarity for readers.  See lines: 135-137:  “The survey contained questions intended at generating qualitative descriptions of the Article 5.3 policies and their functioning, as well as identifying relevant stakeholders in study states.”  See lines: 174-176:  “The questionnaire had two parts: one enquiring about the Article 5.3 policy (issuing authority, scope, formation and functioning of overseeing/implementing body, and reporting of violations) and the other about the complaints received and action taken (if any).” |
