# Supplementary material for: Implementing the World Health Organization - Framework Convention on Tobacco Control Article 5.3: A qualitative study in 17 Indian states
Source: PLOS Glob Public Health. 2026 Jul 2;6(7):e0006522. doi: 10.1371/journal.pgph.0006522 (PMC13327259; doi:10.1371/journal.pgph.0006522)
Supplement: Peer review history

# Peer review: Decision Letter 0

**DOI:** 10.1371/journal.pgph.0006522.r001

**Authors:** Ilse Bloom

12 Dec 2025

PGPH-D-25-02462

Implementing the World Health Organization - Framework Convention on Tobacco Control Article 5.3: a qualitative study in 17 Indian states

PLOS Global Public Health

Dear Dr. Bhojani,

Thank you for submitting your manuscript to PLOS Global Public Health. After careful consideration, we feel that it has merit but does not fully meet PLOS Global Public Health’s publication criteria as it currently stands. Therefore, we invite you to submit a revised version of the manuscript that addresses the points raised during the review process.

The manuscript has been evaluated by three reviewers, and their comments are available below.

The reviewers have raised a number of major concerns. Reviewers have highlighted issues related to the background for the study, its focus as well as the discussion and interpretation of findings.

Could you please carefully revise the manuscript to address all comments raised?

Please submit your revised manuscript by Jan 25 2026 11:59PM. If you will need more time than this to complete your revisions, please reply to this message or contact the journal office at globalpubhealth@plos.org. When you're ready to submit your revision, log on to https://www.editorialmanager.com/pgph/ and select the 'Submissions Needing Revision' folder to locate your manuscript file.

Please include the following items when submitting your revised manuscript:

- A rebuttal letter that responds to each point raised by the editor and reviewer(s). You should upload this letter as a separate file labeled 'Response to Reviewers'.
- A marked-up copy of your manuscript that highlights changes made to the original version. You should upload this as a separate file labeled 'Revised Manuscript with Track Changes'.
- An unmarked version of your revised paper without tracked changes. You should upload this as a separate file labeled 'Manuscript'.

Guidelines for resubmitting your figure files are available below the reviewer comments at the end of this letter.

We look forward to receiving your revised manuscript.

Kind regards,

Ilse Bloom

Staff Editor

PLOS Global Public Health

Journal Requirements:

1.  Please amend your detailed Financial Disclosure statement. This is published with the article. It must therefore be completed in full sentences and contain the exact wording you wish to be published.

1. Please clarify all sources of funding (financial or material support) for your study. List the grants (with grant number) or organizations (with url) that supported your study, including funding received from your institution.

2. State the initials, alongside each funding source, of each author to receive each grant.

3. State what role the funders took in the study. If the funders had no role in your study, please state: “The funders had no role in study design, data collection and analysis, decision to publish, or preparation of the manuscript.”

4. If any authors received a salary from any of your funders, please state which authors and which funders.

2. Please provide separate figure files in .tif or .eps format.

For more information about figure files please see our guidelines:

https://journals.plos.org/globalpublichealth/s/figures

https://journals.plos.org/globalpublichealth/s/figures#loc-file-requirements

3. We have noticed that you have uploaded Supporting Information files, but you have not included a list of legends. Please add a full list of legends for your Supporting Information files after the references list.

4. If the reviewer comments include a recommendation to cite specific previously published works, please review and evaluate these publications to determine whether they are relevant and should be cited. There is no requirement to cite these works unless the editor has indicated otherwise.

[Note: HTML markup is below. Please do not edit.]

Reviewers' comments:

Reviewer's Responses to Questions

**Comments to the Author**

1. Does this manuscript meet PLOS Global Public Health’s publication criteria? Is the manuscript technically sound, and do the data support the conclusions? The manuscript must describe methodologically and ethically rigorous research with conclusions that are appropriately drawn based on the data presented.

Reviewer #1: Yes

Reviewer #2: Yes

Reviewer #3: Yes

**********

2. Has the statistical analysis been performed appropriately and rigorously?

Reviewer #1: Yes

Reviewer #2: Yes

Reviewer #3: N/A

**********

3. Have the authors made all data underlying the findings in their manuscript fully available (please refer to the Data Availability Statement at the start of the manuscript PDF file)?

The PLOS Data policy requires authors to make all data underlying the findings described in their manuscript fully available without restriction, with rare exception. The data should be provided as part of the manuscript or its supporting information, or deposited to a public repository. For example, in addition to summary statistics, the data points behind means, medians and variance measures should be available. If there are restrictions on publicly sharing data—e.g. participant privacy or use of data from a third party—those must be specified.

Reviewer #1: Yes

Reviewer #2: Yes

Reviewer #3: Yes

**********

4. Is the manuscript presented in an intelligible fashion and written in standard English?

PLOS Global Public Health does not copyedit accepted manuscripts, so the language in submitted articles must be clear, correct, and unambiguous. Any typographical or grammatical errors should be corrected at revision, so please note any specific errors here.

Reviewer #1: Yes

Reviewer #2: Yes

Reviewer #3: No

**********

5. Review Comments to the Author

Please use the space provided to explain your answers to the questions above. You may also include additional comments for the author, including concerns about dual publication, research ethics, or publication ethics. (Please upload your review as an attachment if it exceeds 20,000 characters)

Reviewer #1: It is a commendable study, well structured and useful to get updated on the current status of the implementation of the Article 5.3 in Indian States. I compliment all co-authors for the their due diligence right from conceptualisation to this stage in publishing it.

My comments relate to the mention of the names of the States and their nos. as pointed out initially. In my view it looses the advocacy appeal and impact value otherwise.

The hesitancy or limitation of not mentioning the vital stakeholder- the Ministry of Health and Family Welfare who should be constantly promoting and supporting the State-based activities with a clear directive for its undertaking at the highest level- the State Cabinet is understood but I think that is where

the research outcomes should mention it in a way that is acceptable to all.

Please consider working on this point.

Reviewer #2: The research is on an Important, policy relevant topic (state level implementation of WHO FCTC Article 5.3) and I thoroughly enjoyed reading it. The manuscript has real world utility and a solid mixed-methods backbone.

The manuscript cites the same group of states as 64.3% of India’s population but uses different absolute numbers in different sections (e.g., ~999M vs ~909M). Please recompute using a single 2025 reference population and keep one absolute figure throughout. Likewise, ensure a single anchor date for the policy counts (e.g., “as of July 2024” vs “by 2025”) and harmonize the narrative.

“TCD’s partner organization(s)” appears without a prior definition in the main text; spell out Tobacco Control Department (TCD), Vital Strategies at first mention and clarify facilitators’ roles to avoid perceived influence.

Expand Methods to include:Coder reliability: who coded; proportion double-coded; how disagreements were resolved; any intercoder agreement metrics or consensus procedures.

Data completeness: only a subset of FGDs were audio-recorded; specify how non-recorded sessions were documented and how you minimized loss of nuance.

Add a note on how you manged hierarchical effects in mixed-seniority groups.

The respondent mix appears weighted toward NGO/partner stakeholders, with relatively few direct government decision-makers. Add a paragraph on selection rationale, and potential biases

Spellings to fix:

“WHO Framework Convection on Tobacco Control” → Convention.

“industry interreference” → interference (appears in Results/Impact section).

“ACKNOLWEDGEMENTS” → ACKNOWLEDGEMENTS (section header).

“LiberOffice” → LibreOffice; “propriety software” → proprietary.

Table 1 column header “Whether such a body overswing …” → overseeing;

Reviewer #3: This is a very interesting paper on the implementation on Art. 5.3 policies at the subnational level in India. I’ve enjoyed reading it and it will be a nice addition to the literature, based on a great dataset. To make sure that it has the impact that it deserves, I make several suggestions that the authors may wish to conside,

1) Abstract: You state in the methods: “The inquiry focused on policy awareness/dissemination, barriers and facilitators in policy implementation, the perceived policy impact, and respondents' suggestions for improvements.” But in the findings section you only report on barriers and facilitators. The conclusions remain unspecific (“several systemic challenges”… “a few positive practices from past”) – consider expanding on this (to save words, you could tighten the methods and phrasing elsewhere (e.g., “barriers and facilitators to implement these policies” • “barriers and facilitators to policy implementation)

2) Introduction (Paragraphs 1 and 2): When you speak about “implementation gap” and “implementation” more generally, it remains unclear if you refer to the implementation of the FCTC (ie., adoption of national/subnational policies) or the implementation of the national/subnational policies. It would be very important in my eyes to make this explicit.

3) Introduction: The introduction currently does not cover the so what – i.e., so why does it matter? Consider adding a short paragraph at the end.

4) Results/ rest: Based on methods/result, you only focused on barriers and not also on facilitators and you also cover challenges to policy adoption and implementation (and not just implementation). This seems inconsistent with abstract and intro – please ensure consistency. Please note that “good practice” are not the same as “facilitators”.

5) Results: It is important to make sure that it’s clear throughout the findings that you report what participants tell you and not as if these were facts. (see specific comments below)

6) Results: Words like “a few” or “many” states are not very precise – you might want to think about where you can report on numbers

7) Discussion: the discussion is slightly repetitive and literature it bring is very limited and narrow. I recommend the authors look, for example, at the tobacco control policy implementation literature more broadly (for example, the barriers are pretty similar to what has been found elsewhere)

8) Conclusion: I’m not sure this is here so much about repeating what you have done and found – it’d be good to strengthen the call for action and make it more specific.

9) Presentation: There are some issues with the writing. For example, articles are at times omitted or used where not needed, abbreviations are not always used consistently (e.g., CSR) and words are used repeatedly (e.g., 1st paragraph of the discussion uses “identified” 3 times). The writing can also be tightened and simplified without losing content in different places (e.g., “Respondents from one of the states” • “Participants from one state”, “Despite a decade since the first such sub-national policy got adopted in India” •”A decade after the adoption of the first subnational policy on Art. 5.3 in India,…” I recommend the authors to have a close read through their paper

Minor points:

- 25 and 112 – Consider starting with “The tobacco industry…”

- 35 – In the methods section of the abstract it sounds as if the survey was only used to inform the second stage and that the data wasn’t included in the final analysis. If this is not the case, please clarify.

- 36 – Consider stating “adopted Article 5.3 policies” – please also correct where else this appears.

- 36 – “that consented” – this sounds a bit odd as states cannot really give consent

- 37 – “self-administered survey” – “maybe an “online survey”?

- 61 – “from the past” instead of “from past”?

- 112 – What tactics? You could mention some examples – the journal audience is not just TC specialists. It would also be good to back this up with references.

- 116 – not clear what the “such” refers to as it’s not described in the previous sentence.

- 117 – “member governments” – maybe better to just put “members” as it’s not just the governments that are members and as also the EU is a member.

- 118 – are there any later figures? 2020 seems quite a while ago

- 119 – you might want to explain what the implementation guidelines are and reference them (it would also be good to reference the WHO FCTC)

- 122 – “huge implementation gap” – this either needs referencing or you need to end the sentence with “:”.

- 124-5 – “the Article 5.3 had remained one of the least implemented provisions of the treaty.” – consider adding this to the previous paragraph and checking if you need the info before for the flow of your introduction.

- 128 – considering adding the latest Index

- 129-31 – it would be good to clarify what the comparison here (compared to the 2021 report); I also think there were 90 countries in the report and I reckon you say 80 because 80 were in 2021 one.

- 133 – this could be stronger as you haven’t clearly state the gap – is it that the there is little knowledge into Art. 5.3 policy implementation?

- 135-7 – it would be good to back these two sentences up with references.

- 137-8 – you could make this a bit more specific – e.g., where does India sit on the GTIII?

- 146 – “in the implementation” instead of “in implementation”?

- 146-50 – This sentence is very long and quite hard to follow – can you simplify or split it into two?

- 155 – “Article” instead of “article”

- 163-4 – “more from a positivist perspective – what does this mean? I’m not sure you want to go into positivist vs interpretivist here as it raises questions about the knowledge generated here if there are different epistemological frames used

- 169 – “implicit knowledge” sounds a bit odd – you could maybe just say “knowledge”?

- 169/70 – you might want to clarify here when you identified those. Earlier you said that today there are more so it could be good to be clear

- 171 – can you say more about the administrative approval? From who did you need that and why?

- 174-5 – this needs a reference

- 175-8 – it would be good to mention this when you mention union territories for the first time – the reader may not be familiar with what they are.

- 181-4 – it’s good that you state this but it may not be ideal here since you have not yet described these tools. I recommend you mention this when you describe the survey and then refer to it again when it comes to the FGDs.

- 184 – what are “field pilots” and why where they done in non-study states? Does this then mean that these didn’t have such policies?

- 186 – why did you select the survey participants? This is quite important as you drew then on their information for the next part. And how do you know they filled it in close with the officials? Also, how many participants did you approach and how many completed it?

- 201 – What is TCD?

- 201-2 – “facilitators” and “facilitating” in one sentence. I’m also not so clear what you mean here by “facilitating” as there are quite a few different actors – who organised it? Who invited participants? Who actually ran the focus group sessions?

- 204 – Here you state that the facilitators helped with the sampling, further up you and in the abstract you say that the surveys helped to identify relevant stakeholders – it would be good if you could clarify? Did the facilitators analyse the data from the survey, for example?

- 209 – “Potential participants” instead of ”The participants”

- 210 – How many did you invite and how many did participate?

- 217 – why did you ask about policy adoption given that your focus is on implementation?

- 218 – What do you mean by “implementation mechanisms”?

- 218 – for FGDs, we usually say participants instead of respondents (also 238 and please check throughout the manuscript)

- 222 – who took notes? 1 person or more?

- 227 – “FGDs” instead of “FGD”

- 229 – can you describe what you mean by “thematic content analysis”? References could also help.

- 243 – this is inconsistent with the methods where you say: “Respondents were

- 188 representatives from civil society organizations working in tobacco control in study states”

- 248 – What was the average number of participants per FGDs? And what was the range?

- 248f - Your FGDs seem to have been quite large (and larger than standard FGDs) which raises some concerns, particularly if they only last 40 minutes given individuals very little time to speak, etc. It would be good to clarify why you thought the FGDs was the best approach (and not, for example, online survey) and why you decided to not split it up into more than one session?

- 248 – were the survey participants also part of the FGDs? It would be important to clarify.

- 257 – Start a new paragraph with “Typical,…”

- 257-64 – Consider changing the structure of this (e.g., When presenting the results, we do not mention state names but have assigned each state a number. This is to avoid…) Please also consider if this is not rather about the methods

- 278-9 – you could provide references here.

- 279-81 – three times “issued”

- Table 1 – “Was such” instead of “Whether such” for the last two questions

- 296-306 – I had thought that you wanted to keep this here descriptive, so consider if this doesn’t rather go under challenges?

- 310-1 – For me, this sentence is discussion rather than your findings.

- 311 – For how this section is written, it doesn’t become clear what these two are. Make it explicit in how you present the findings.

- 328 – “believed” might not be a good word as you ultimately don’t know what people believe, better use report, state or similar

- 345 – maybe clarify that this about awareness among participants?

- 356-8 – “However, even in these states, the detailed knowledge about various provisions in these policies was typically limited to health department officers.” – you need to make clear that this what the participants said – e.g., participants reported/perceived…

- 370 – This seems to be closely tied to “Low awareness” as it is one explanation for it? So why are they separate? It’s also not so clear if dissemination is about dissemination within the public sector or to the public or all.

- 371-6 – Also here, you need to make clear that this is what participants said (same 387-92)

- 395 – I think you can be more specific here and say in our many states this was based on the survey data?

- 397-8 – “Such was likely a case for many more states” – maybe rather “Participants suspected this could be the case…” or is this you suspicion? If it’s yours, then it’s more a discussion point

- 408 – is this really just about “implementation guidance”? Something like having a clear definition of the tobacco industry is maybe not just about guiding implementation? Point 2 and 3 are then more about enforcement and 4 about oversight, etc.

- 427 – the quote talks about “general lack of awareness” is this not your first theme?

- 446 – can you clarify what you mean by front group?

- 459 – this paragraph interrupts the narrative here as it’s not really about implementation challenges but about perceived vulnerability

- 482-3 – Please clarify that this is about participants’ reporting

- 485 – “from at least 3 states” – can you not specify how many it was?

- 486-7 – “mentioning the need for prior permission/procedure to be followed by the tobacco industry in meeting government officials” – it’s not clear what the permission is for.

- 497 – “Though not mandated by 5.3 policies” – so this is not really about implementation of the policy, so is this really in the right section?

- 526 - “three of the states reported” – states cannot report (also 564)

- 526 – “groups” instead of “group”

- 538 – I’m not sure it’s very clear what you mean by normative measures and norms. You could for example, already bring this up in the introduction and define it there. Also here, please make sure it’s clear that this was reported by participants.

- 564 – here you mention 7 states, there are 8 in the table (heading “brief account of complaints” – and for Assam and Meghalaya it says: “There was probably no formal complaint” – make sure you are consistent and use the correct wording

- 591 – Here it’s CSR, elsewhere its corporate social responsibility – it’d be good to introduce the abbreviation the first time and then use it consistently.

- 627 – “implementation remains very limited” – can you explain what you mean by this?

- 651 – What do you mean by “time-bound resolution procedures”? I don’t think this came up before. And why are complaints hindering the implementation of the policy? Is this about lack of definition as well? Then this needs to be clearer.

- 705 – You could connect the limitation you identified to the literature (e.g., social desirability bias).

- 705 – While I understand that organising the sessions was challenging, I wonder if you did anything to navigate the power dynamics (e.g., did you invite people to contact you afterwards if they wanted to share anything else?)

- 705 – your reflection here made me wonder if calling what you have done a FGDs is actually the most appropriate word. Or was it maybe a workshop? Please reconsider your label. .

- Figure 1: The text is quite blurry and hard to read

**********

6. PLOS authors have the option to publish the peer review history of their article (what does this mean?). If published, this will include your full peer review and any attached files.

**Do you want your identity to be public for this peer review?**  If you choose “no”, your identity will remain anonymous but your review may still be made public.

For information about this choice, including consent withdrawal, please see our Privacy Policy.

Reviewer #1: **Yes:**  Dr. Rakesh Gupta

Reviewer #2: No

Reviewer #3: No

**********

[NOTE: If reviewer comments were submitted as an attachment file, they will be attached to this email and accessible via the submission site. Please log into your account, locate the manuscript record, and check for the action link "View Attachments". If this link does not appear, there are no attachment files.]

-->Figure Resubmissions:

While revising your submission, we strongly recommend that you use PLOS’s NAAS tool (https://ngplosjournals.pagemajik.ai/artanalysis) to test your figure files. NAAS can convert your figure files to the TIFF file type and meet basic requirements (such as print size, resolution), or provide you with a report on issues that do not meet our requirements and that NAAS cannot fix.

After uploading your figures to PLOS’s NAAS tool - https://ngplosjournals.pagemajik.ai/artanalysis, NAAS will process the files provided and display the results in the "Uploaded Files" section of the page as the processing is complete. If the uploaded figures meet our requirements (or NAAS is able to fix the files to meet our requirements), the figure will be marked as "fixed" above. If NAAS is unable to fix the files, a red "failed" label will appear above. When NAAS has confirmed that the figure files meet our requirements, please download the file via the download option, and include these NAAS processed figure files when submitting your revised manuscript.

Attachment Submitted filename: WHO FCTC Article 5.3_PGPH-D-25-02462_RG- Editor_20250919.pdf

# Author comment: Author response to Decision Letter 1

**DOI:** 10.1371/journal.pgph.0006522.r002

30 Dec 2025

Attachment Submitted filename: Response to Reviewers.docx

# Peer review: Decision Letter 1

**DOI:** 10.1371/journal.pgph.0006522.r003

**Authors:** Ilse Bloom, Katrien Janin

26 Feb 2026

PGPH-D-25-02462R1

Implementing the World Health Organization - Framework Convention on Tobacco Control Article 5.3: a qualitative study in 17 Indian states

PLOS Global Public Health

Dear Dr. Bhojani,

Thank you for submitting your manuscript to PLOS Global Public Health. After careful consideration, we feel that it has merit but does not fully meet PLOS Global Public Health’s publication criteria as it currently stands. Therefore, we invite you to submit a revised version of the manuscript that addresses the points raised during the review process.

The manuscript has been evaluated by one reviewer, and their comments are available below. The reviewer have raised a number of concerns that need attention. They recommend tightening the manuscript overall, particularly the Results section, and for the qualitative quotes to be integrated more effectively.

Could you please revise the manuscript to carefully address the concerns raised?

Please submit your revised manuscript by Apr 11 2026 11:59PM. If you will need more time than this to complete your revisions, please reply to this message or contact the journal office at globalpubhealth@plos.org. When you're ready to submit your revision, log on to https://www.editorialmanager.com/pgph/ and select the 'Submissions Needing Revision' folder to locate your manuscript file.

Please include the following items when submitting your revised manuscript:

- A letter that responds to each point raised by the editor and reviewer(s). You should upload this letter as a separate file labeled 'Response to Reviewers'.
- A marked-up copy of your manuscript that highlights changes made to the original version. You should upload this as a separate file labeled 'Revised Manuscript with Track Changes'.
- An unmarked version of your revised paper without tracked changes. You should upload this as a separate file labeled 'Manuscript'.

Guidelines for resubmitting your figure files are available below the reviewer comments at the end of this letter.

We look forward to receiving your revised manuscript.

Kind regards,

Katrien G. Janin, PhD

Staff Editor

PLOS Global Public Health

Journal Requirements:

If the reviewer comments include a recommendation to cite specific previously published works, please review and evaluate these publications to determine whether they are relevant and should be cited. There is no requirement to cite these works unless the editor has indicated otherwise.

Additional Editor Comments (if provided):

[Note: HTML markup is below. Please do not edit.]

Reviewers' comments:

Reviewer's Responses to Questions

**Comments to the Author**

1. If the authors have adequately addressed your comments raised in a previous round of review and you feel that this manuscript is now acceptable for publication, you may indicate that here to bypass the “Comments to the Author” section, enter your conflict of interest statement in the “Confidential to Editor” section, and submit your "Accept" recommendation.

Reviewer #3: All comments have been addressed

**********

2. Does this manuscript meet PLOS Global Public Health’s publication criteria? Is the manuscript technically sound, and do the data support the conclusions? The manuscript must describe methodologically and ethically rigorous research with conclusions that are appropriately drawn based on the data presented.

Reviewer #3: Yes

**********

3. Has the statistical analysis been performed appropriately and rigorously?

Reviewer #3: N/A

**********

4. Have the authors made all data underlying the findings in their manuscript fully available (please refer to the Data Availability Statement at the start of the manuscript PDF file)?

The PLOS Data policy requires authors to make all data underlying the findings described in their manuscript fully available without restriction, with rare exception. The data should be provided as part of the manuscript or its supporting information, or deposited to a public repository. For example, in addition to summary statistics, the data points behind means, medians and variance measures should be available. If there are restrictions on publicly sharing data—e.g. participant privacy or use of data from a third party—those must be specified.

Reviewer #3: Yes

**********

5. Is the manuscript presented in an intelligible fashion and written in standard English?

PLOS Global Public Health does not copyedit accepted manuscripts, so the language in submitted articles must be clear, correct, and unambiguous. Any typographical or grammatical errors should be corrected at revision, so please note any specific errors here.

Reviewer #3: No

**********

6. Review Comments to the Author

Please use the space provided to explain your answers to the questions above. You may also include additional comments for the author, including concerns about dual publication, research ethics, or publication ethics. (Please upload your review as an attachment if it exceeds 20,000 characters)

Reviewer #3: (No Response)

**********

7. PLOS authors have the option to publish the peer review history of their article (what does this mean?). If published, this will include your full peer review and any attached files.

**Do you want your identity to be public for this peer review?**  If you choose “no”, your identity will remain anonymous but your review may still be made public.

For information about this choice, including consent withdrawal, please see our Privacy Policy.

Reviewer #3: No

**********

[NOTE: If reviewer comments were submitted as an attachment file, they will be attached to this email and accessible via the submission site. Please log into your account, locate the manuscript record, and check for the action link "View Attachments". If this link does not appear, there are no attachment files.]

-->Figure Resubmissions:

While revising your submission, we strongly recommend that you use PLOS’s NAAS tool (https://ngplosjournals.pagemajik.ai/artanalysis) to test your figure files. NAAS can convert your figure files to the TIFF file type and meet basic requirements (such as print size, resolution), or provide you with a report on issues that do not meet our requirements and that NAAS cannot fix.

After uploading your figures to PLOS’s NAAS tool - https://ngplosjournals.pagemajik.ai/artanalysis, NAAS will process the files provided and display the results in the "Uploaded Files" section of the page as the processing is complete. If the uploaded figures meet our requirements (or NAAS is able to fix the files to meet our requirements), the figure will be marked as "fixed" above. If NAAS is unable to fix the files, a red "failed" label will appear above. When NAAS has confirmed that the figure files meet our requirements, please download the file via the download option, and include these NAAS processed figure files when submitting your revised manuscript.

# Author comment: Author response to Decision Letter 2

**DOI:** 10.1371/journal.pgph.0006522.r004

11 Apr 2026

Attachment Submitted filename: response to peer review comments_April 11.docx

# Peer review: Decision Letter 2

**DOI:** 10.1371/journal.pgph.0006522.r005

**Authors:** Ilse Bloom, Katrien Janin, Meghnath Dhimal

20 Apr 2026

PGPH-D-25-02462R2

Implementing the World Health Organization - Framework Convention on Tobacco Control Article 5.3: a qualitative study in 17 Indian states

PLOS Global Public Health

Dear Dr. Bhojani,

Thank you for submitting your manuscript to PLOS Global Public Health. After careful consideration, we feel that it has merit but does not fully meet PLOS Global Public Health’s publication criteria as it currently stands. Therefore, we invite you to submit a revised version of the manuscript that addresses the points raised during the review process.

Please submit your revised manuscript by May 20 2026 11:59PM. If you will need more time than this to complete your revisions, please reply to this message or contact the journal office at globalpubhealth@plos.org. When you're ready to submit your revision, log on to https://www.editorialmanager.com/pgph/ and select the 'Submissions Needing Revision' folder to locate your manuscript file.

Please include the following items when submitting your revised manuscript:

- A letter that responds to each point raised by the editor and reviewer(s). You should upload this letter as a separate file labeled 'Response to Reviewers'.
- A marked-up copy of your manuscript that highlights changes made to the original version. You should upload this as a separate file labeled 'Revised Manuscript with Track Changes'.
- An unmarked version of your revised paper without tracked changes. You should upload this as a separate file labeled 'Manuscript'.

Guidelines for resubmitting your figure files are available below the reviewer comments at the end of this letter.

As the corresponding author, your ORCID iD is verified in the submission system and will appear in the published article. PLOS supports the use of ORCID, and we encourage all coauthors to register for an ORCID iD and use it as well. Please encourage your coauthors to verify their ORCID iD within the submission system before final acceptance, as unverified ORCID iDs will not appear in the published article. *Only*  the individual author can complete the verification step; PLOS staff *cannot*  verify ORCID iDs on behalf of authors.

We look forward to receiving your revised manuscript.

Kind regards,

Meghnath Dhimal, Ph.D.

Academic Editor

PLOS Global Public Health

Journal Requirements:

If the reviewer comments include a recommendation to cite specific previously published works, please review and evaluate these publications to determine whether they are relevant and should be cited. There is no requirement to cite these works unless the editor has indicated otherwise.

Please review your reference list to ensure that it is complete and correct. If you have cited papers that have been retracted, please include the rationale for doing so in the manuscript text, or remove these references and replace them with relevant current references. Any changes to the reference list should be mentioned in the rebuttal letter that accompanies your revised manuscript. If you need to cite a retracted article, indicate the article’s retracted status in the References list and also include a citation and full reference for the retraction notice.

Additional Editor Comments (if provided):

You have stated a *qualitative study design*  in your title; however, the methodology section mentions the use of an *online survey* , which may confuse readers because surveys are typically associated with quantitative research. Please clarify this by explaining that the online survey was used solely to collect qualitative data, and that it included open‑ended questions based on the interview guide or checklist. This clarification will help readers understand that the survey functioned as an alternative mode for gathering narrative, qualitative responses rather than quantitative data.

[Note: HTML markup is below. Please do not edit.]

Reviewers' comments:

Reviewer's Responses to Questions

**Comments to the Author**

1. If the authors have adequately addressed your comments raised in a previous round of review and you feel that this manuscript is now acceptable for publication, you may indicate that here to bypass the “Comments to the Author” section, enter your conflict of interest statement in the “Confidential to Editor” section, and submit your "Accept" recommendation.

Reviewer #3: All comments have been addressed

**********

2. Does this manuscript meet PLOS Global Public Health’s publication criteria? Is the manuscript technically sound, and do the data support the conclusions? The manuscript must describe methodologically and ethically rigorous research with conclusions that are appropriately drawn based on the data presented.

Reviewer #3: Yes

**********

3. Has the statistical analysis been performed appropriately and rigorously?

Reviewer #3: N/A

**********

4. Have the authors made all data underlying the findings in their manuscript fully available (please refer to the Data Availability Statement at the start of the manuscript PDF file)?

The PLOS Data policy requires authors to make all data underlying the findings described in their manuscript fully available without restriction, with rare exception. The data should be provided as part of the manuscript or its supporting information, or deposited to a public repository. For example, in addition to summary statistics, the data points behind means, medians and variance measures should be available. If there are restrictions on publicly sharing data—e.g. participant privacy or use of data from a third party—those must be specified.

Reviewer #3: Yes

**********

5. Is the manuscript presented in an intelligible fashion and written in standard English?

PLOS Global Public Health does not copyedit accepted manuscripts, so the language in submitted articles must be clear, correct, and unambiguous. Any typographical or grammatical errors should be corrected at revision, so please note any specific errors here.

Reviewer #3: Yes

**********

6. Review Comments to the Author

Please use the space provided to explain your answers to the questions above. You may also include additional comments for the author, including concerns about dual publication, research ethics, or publication ethics. (Please upload your review as an attachment if it exceeds 20,000 characters)

Reviewer #3: Thank you for the opportunity to look at this manuscript again. I’m glad to see that the authors found the feedback helpful and they have done a great job responding to all comments. I only have a few minor comments to add:

- Abstract: Introduction – “their policies/ “this policy” – is a bit confusing for readers. It’d be good to clarify in the second sentence what you mean by “this policy”.

- Abstract: Methods – “Thematic content analysis” is usual – was a thematic or content analysis? It also appears again in the methods where you also refer to Braun and Clarke, I reckon you did (reflexive) thematic analysis?

- Abstract: Results – consider using “remained” instead of “remain” for consistency.

- Introduction: “about 102” – If it is a 102, you can remove “about”.

- Introduction: “in countries that have already adopted” – do you mean “ratified”?

- Methods: “are union territories at the time of the study” – “were union…”?

- Methods: “These states account…” – I suggest you say “These 17 states” as in the previous sentence you refer to three so the reader may wonder what “these states” refer to.

- Methods: “of the Vital Strategies” – is it not just “of Vital Strategies”? It’s mentioned a few times.

- Methods: “These partner organizations were trained/calibrated through…” The next sentence also starts with “These”. I suggest you revise the phrasing.

- Results: “Fig 1 Characteristics of FGD participants” – consider moving this up a paragraph so that the figure is located after the paragraph where it is mentioned.

- Results: “In the rest of the six states” – is not very clear, maybe “In the other six states”?

- Discussion: “for 5.3 of the WHO FCTC” – consider inserting “Article”

- Conclusion – You might want to mention the WHO FCTC in the first sentence

- I suggest the authors closely reread the manuscript and check for issues similar to those I picked up (e.g., connections, clear reference points)

**********

7. PLOS authors have the option to publish the peer review history of their article (what does this mean?). If published, this will include your full peer review and any attached files.

**Do you want your identity to be public for this peer review?**  If you choose “no”, your identity will remain anonymous but your review may still be made public.

For information about this choice, including consent withdrawal, please see our Privacy Policy.

Reviewer #3: No

**********

[NOTE: If reviewer comments were submitted as an attachment file, they will be attached to this email and accessible via the submission site. Please log into your account, locate the manuscript record, and check for the action link "View Attachments". If this link does not appear, there are no attachment files.]

Figure Resubmissions:

While revising your submission, we strongly recommend that you use PLOS’s NAAS tool (https://ngplosjournals.pagemajik.ai/artanalysis) to test your figure files. NAAS can convert your figure files to the TIFF file type and meet basic requirements (such as print size, resolution), or provide you with a report on issues that do not meet our requirements and that NAAS cannot fix.

After uploading your figures to PLOS’s NAAS tool - https://ngplosjournals.pagemajik.ai/artanalysis, NAAS will process the files provided and display the results in the "Uploaded Files" section of the page as the processing is complete. If the uploaded figures meet our requirements (or NAAS is able to fix the files to meet our requirements), the figure will be marked as "fixed" above. If NAAS is unable to fix the files, a red "failed" label will appear above. When NAAS has confirmed that the figure files meet our requirements, please download the file via the download option, and include these NAAS processed figure files when submitting your revised manuscript.

# Author comment: Author response to Decision Letter 3

**DOI:** 10.1371/journal.pgph.0006522.r006

27 Apr 2026

Attachment Submitted filename: Response to Reviewer Comments_24 April 2026.docx

# Editor report: Decision Letter 3

**DOI:** 10.1371/journal.pgph.0006522.r007

**Authors:** Ilse Bloom, Katrien Janin, Meghnath Dhimal, Meghnath Dhimal

5 May 2026

Implementing the World Health Organization - Framework Convention on Tobacco Control Article 5.3: a qualitative study in 17 Indian states

PGPH-D-25-02462R3

Dear Dr. Bhojani,

We are pleased to inform you that your manuscript 'Implementing the World Health Organization - Framework Convention on Tobacco Control Article 5.3: a qualitative study in 17 Indian states' has been provisionally accepted for publication in PLOS Global Public Health.

Before your manuscript can be formally accepted you will need to complete some formatting changes, which you will receive in a follow up email. A member of our team will be in touch with a set of requests.

Please note that your manuscript will not be scheduled for publication until you have made the required changes, so a swift response is appreciated.

IMPORTANT: The editorial review process is now complete. PLOS will only permit corrections to spelling, formatting or significant scientific errors from this point onwards. Requests for major changes, or any which affect the scientific understanding of your work, will cause delays to the publication date of your manuscript.

If your institution or institutions have a press office, please notify them about your upcoming paper to help maximize its impact. If they'll be preparing press materials, please inform our press team as soon as possible -- no later than 48 hours after receiving the formal acceptance. Your manuscript will remain under strict press embargo until 2 pm Eastern Time on the date of publication. For more information, please contact globalpubhealth@plos.org.

Thank you again for supporting Open Access publishing; we are looking forward to publishing your work in PLOS Global Public Health.

Best regards,

Meghnath Dhimal, Ph.D.

Academic Editor

PLOS Global Public Health

***********************************************************

Reviewer Comments (if any, and for reference):
